# EPMA-World Congress 2015: Bonn, Germany. 3-5 September 2015

**DOI:** 10.1186/s13167-016-0054-6

**Published:** 2016-05-09

**Authors:** Jella-Andrea Abraham, Olga Golubnitschaja, Ildar Akhmetov, Russell J. Andrews, Leonidas Quintana, Russell J. Andrews, Babak Baban, Jun Yao Liu, Xu Qin, Tailing Wang, Mahmood S. Mozaffari, Viktoriia V. Bati, Tamara V. Meleshko, Olga B. Levchuk, Nadiya V. Boyko, Joanna Bauer, Ewa Boerner, Halina Podbielska, Alojz Bomba, Viktor O. Petrov, Volodymyr G. Drobnych, Rostyslav V. Bubnov, Oksana M. Bykova, Nadiya V. Boyko, Hans-Peter Brunner-La Rocca, Lutz Fleischhacker, Olga Golubnitschaja, Frank Heemskerk, Thomas Helms, Tiny Jaarsma, Judita Kinkorová, Jan Ramaekers, Peter Ruff, Ivana Schnur, Emilio Vanoli, Jose Verdu, Hans-Peter Brunner-La Rocca, Rostyslav V. Bubnov, Sergiy A. Grabovetskyi, Olena M. Mykhalchenko, Natalia O. Tymoshok, Oleksandr B. Shcherbakov, Igor P. Semeniv, Mykola Y. Spivak, Rostyslav V. Bubnov, Tetyana V. Ostapenko, Rostyslav V. Bubnov, Nazarii M. Kobyliak, Nadiya M. Zholobak, Mykola Ya. Spivak, John Paul Cauchi, Dmitrii Cherepakhin, Marina Bakay, Artem Borovikov, Sergey Suchkov, Barbara Cieślik, Agnieszka Migasiewicz, Maria-Luiza Podbielska, Markus Pelleter, Agnieszka Giemza, Halina Podbielska, Sebahattin Cirak, Marzia Del Re, Paola Bordi, Valentina Citi, Marta Palombi, Carmine Pinto, Marcello Tiseo, Romano Danesi, Lukas Einhorn, Judit Fazekas, Martina Muhr, Alexandra Schoos, Lucia Panakova, Ina Herrmann, Krisztina Manzano-Szalai, Kumiko Oida, Edda Fiebiger, Josef Singer, Erika Jensen-Jarolim, Arpiné A. Elnar, Nadia Ouamara, Nadiya Boyko, Xavier Coumoul, Jean-Philippe Antignac, Bruno Le Bizec, Gauthier Eppe, Jenny Renaut, Torsten Bonn, Cédric Guignard, Margherita Ferrante, Maria Liusa Chiusano, Salvatore Cuzzocrea, Gerard O’Keeffe, John Cryan, Michelle Bisson, Amina Barakat, Ihsane Hmamouchi, Nasser Zawia, Anumantha Kanthasamy, Glen E. Kisby, Rui Alves, Oscar Villacañas Pérez, Kim Burgard, Peter Spencer, Norbert Bomba, Martin Haranta, Nina Zaitseva, Irina May, Stéphanie Grojean, Mathilde Body-Malapel, Florencia Harari, Raul Harari, Kristina Yeghiazaryan, Olga Golubnitschaja, Vittorio Calabrese, Christophe Nemos, Rachid Soulimani, Maria E. Evsevyeva, Elena A. Mishenko, Zurida V. Kumukova, Evgeniy V. Chudnovsky, Tatyana A. Smirnova, Maria E. Evsevyeva, Ludmila V. Ivanova, Michail V. Eremin, Maria V. Rostovtseva, Maria E. Evsevyeva, Michail V. Eremin, Vladimir I. Koshel, Oksana V. Sergeeva, Nadesgda M. Konovalova, Shantanu Girotra, Olga Golubnitschaja, Olga Golubnitschaja, Manuel Debald, Walther Kuhn, Kristina Yeghiazaryan, Rostyslav V. Bubnov, Vadym M. Goncharenko, Ulyana Lushchyk, Godfrey Grech, Katarzyna Konieczka, Olga Golubnitschaja, Jan Jaap Erwich, Vincenzo Costigliola, Kristina Yeghiazaryan, Ulrich Gembruch, Vadym M. Goncharenko, Vasyl O. Beniuk, Olga V. Kalenska, Rostyslav V. Bubnov, Vadym M. Goncharenko, Vasyl O. Beniuk, Rostyslav V. Bubnov, Olga Melnychuk, Irina A. Gorbacheva, Lyudmila Y. Orekhova, Vadim V. Tachalov, Olena I. Grechanyk, Rizvan Ya. Abdullaiev, Rostyslav V. Bubnov, Suzanne Hagan, Eilidh Martin, Ian Pearce, Katherine Oliver, Cenk Haytac, Fariz Salimov, Servin Yoksul, Anatoly A. Kunin, Natalia S. Moiseeva, Bernardo Herrera-Imbroda, Sergio del Río-González, Maria Fernanda Lara, Antonia Angulo, Francisco Javier Machuca Santa-Cruz, Bernardo Herrera-Imbroda, Sergio del Río-González, Maria Fernanda Lara, John Ionescu, Alfiya Z. Isamulaeva, Anatoly A. Kunin, Shamil Sh. Magomedov, Aida I. Isamulaeva, Tatjana Josifova, Marko Kapalla, Juraj Kubáň, Olga Golubnitschaja, Vincenzo Costigliola, Vincenzo Costigliola, Marko Kapalla, Juraj Kubáň, Olga Golubnitschaja, Anthony Kent, Tom Fisher, Tilak Dias, Judita Kinkorová, Ondřej Topolčan, Matthias Kohl, Anatoly A. Kunin, Natalia S. Moiseeva, Andrii I. Kurchenko, Vasyl A. Beniuk, Vadym M. Goncharenko, Rostyslav V. Bubnov, Nadiya V. Boyko, Andriy M. Strokan, Julia Kzhyshkowska, Alexandru Gudima, Ksenia S. Stankevich, Victor D. Filimonov, Harald Klüter, Evgeniya M. Mamontova, Sergei I. Tverdokhlebov, Ulyana B. Lushchyk, Viktor V. Novytskyy, Igor P. Babii, Nadiya G. Lushchyk, Lyudmyla S. Riabets, Ivanna I. Legka, Mira Marcus-Kalish, Alexis Mitelpunkt, Tal Galili, Neta Shachar, Yoav Benjamini, Agnieszka Migasiewicz, Markus Pelleter, Joanna Bauer, Ewelina Dereń, Halina Podbielska, Natalia S. Moiseeva, Anatoly A. Kunin, Dmitry A. Kunin, Natalia S. Moiseeva, Yury A. Ippolitov, Dmitry A. Kunin, Alexei N. Morozov, Natalia V. Chirkova, Nakhid T. Aliev, Mahmood S. Mozaffari, Jun Yao Liu, Babak Baban, Mahmood S. Mozaffari, Jun Yao Liu, Rafik Abdelsayed, Xing-Ming Shi, Babak Baban, Jaroslav Novák, Milan Štork, Václav Zeman, Wytze P. Oosterhuis, Elvar Theodorsson, Lyudmila Y. Orekhova, Tatyana V. Kudryavtseva, Elena R. Isaeva, Vadim V. Tachalov, Ekaterina S. Loboda, Mario Pazzagli, Francesca Malentacchi, Irene Mancini, Ivan Brandslund, Pieter Vermeersch, Matthias Schwab, Janja Marc, Ron H. N. van Schaik, Gerard Siest, Elvar Theodorsson, Chiara Di Resta, Matus Pleva, Jozef Juhar, Matus Pleva, Jozef Juhar, Jiří Polívka jr., Filip Janků, Martin Pešta, Jan Doležal, Milena Králíčková, Jiří Polívka, Jiří Polívka, Alena Lukešová, Nina Müllerová, Petr Ševčík, Vladimír Rohan, Kneginja Richter, Lence Miloseva, Günter Niklewski, Kneginja Richter, Jens Acker, Guenter Niklewski, Olga Safonicheva, Vincenzo Costigliola, Olga Safonicheva, Maxim Sautin, Janna Sinelnikova, Sergey Suchkov, Songül Secer, Stephan von Bandemer, Niva Shapira, Aleksandr Shcherbakov, Anatoly A. Kunin, Natalia S. Moiseeva, Bogdan R. Shumilovich, Zhanna Lipkind, Yulia Vorobieva, Dmitry A. Kunin, Anastasiia V. Sudareva, Ivica Smokovski, Tatjana Milenkovic, Arturo Solís-Herrera, María del Carmen Arias-Esparza, Sergey Suchkov, Krishna Chander Sridhar, Olga Golubnitschaja, Maria Studneva, Sihong Song, James Creeden, Мark Мandrik, Sergey Suchkov, Elvar Theodorsson, EFLM, Syed A. M. Tofail, Ondřej Topolčan, Judita Kinkorová, Ondřej Fiala, Marie Karlíková, Šárka Svobodová, Radek Kučera, Radka Fuchsová, Vladislav Třeška, Václav Šimánek, Ladislav Pecen, Jan Šoupal, Štěpán Svačina, Evgeniya Tretyak, Maria Studneva, Sergey Suchkov, Francesca M. Trovato, Giuseppe Fabio Martines, Daniela Brischetto, Daniela Catalano, Giuseppe Musumeci, Guglielmo M. Trovato, George Th. Tsangaris, Athanasios K. Anagnostopoulos, George Th. Tsangaris, Athanasios K. Anagnostopoulos, José Verdú, German Gutiérrez, Jordi Rovira, Marta Martinez, Lutz Fleischhacker, Donna Green, Arthur Garson, Elena Tamburini, Stefano Cuomo, Juan Martinez-Leon, Teresa Abrisqueta, Hans-Peter Brunner-La Rocca, Tiny Jaarsma, Teresa Arredondo, Cecilia Vera, Giuseppe Fico, Olga Golubnitschaja, Fernando Arribas, Martina Onderco, Isabel Vara, on behalf of ZENITH consortium, José Verdú, Francesco Sambo, Barbara Di Camillo, Claudio Cobelli, Andrea Facchinetti, Giuseppe Fico, Riccardo Bellazzi, Lucia Sacchi, Arianna Dagliati, Daniele Segnani, Valentina Tibollo, Manuel Ottaviano, Rafael Gabriel, Leif Groop, Jacqueline Postma, Antonio Martinez, Liisa Hakaste, Tiinamaija Tuomi, Konstantia Zarkogianni, on behalf of MOSAIC consortium, Igor Volchek, Nina Pototskaya, Andrey Petrov, Igor Volchek, Nadezhda Pototskaya, Andrey Petrov, Ülle Voog-Oras, Oksana Jagur, Edvitar Leibur, Priit Niibo, Triin Jagomägi, Minh Son Nguyen, Chris Pruunsild, Dagmar Piikov, Mare Saag, Wei Wang, Wei Wang, Andreas Weinhäusel, Walter Pulverer, Matthias Wielscher, Manuela Hofner, Christa Noehammer, Regina Soldo, Peter Hettegger, Istvan Gyurjan, Ronald Kulovics, Silvia Schönthaler, Gabriel Beikircher, Albert Kriegner, Stephan Pabinger, Klemens Vierlinger, Ayşe Yüzbaşıoğlu, Meral Özgüç

**Affiliations:** Department of Radiology, University of Bonn, Sigmund-Freud-Str. 25, 53105, Bonn, Germany; Market Access at Unicorn, P.O.B. 91, Zhytomyr, 10020 Ukraine; Nanotechnology & Smart Systems, NASA Ames Research Center, Moffett Field, CA USA; Department of Neurosurgery, Valparaiso University Medical School, Valparaiso, Chile; Nanotechnology & Smart Systems, NASA Ames Research Center, Moffett Field, CA USA; Department of Oral Biology, College of Dental Medicine, Georgia Regents University, Augusta, Georgia 30912 USA; Research, Developing and Training Centre of Molecular Microbiology and Mucosal Immunology, Uzhhorod National University, Uzhhorod, Ukraine; “Astra-DIA” Medical Diagnostic Centre, Uzhhorod, Ukraine; Cassovia Life Science, Kysucké Nové Mesto, Slovakia; Department of Biomedical Engineering, Wroclaw University of Technology, Wroclaw, Poland; Faculty of Physiotherapy, Wrocław University School of Physical Education, Wroclaw, Poland; Pavol Jozef Šafárik University, Košice, Slovakia; Cassovia Life Science, Kysucké Nové Mesto, Slovakia; Uzhhorod National University, Uzhhorod, Ukraine; Clinical Hospital “Pheophania” of State Affairs Department, The Centre of Ultrasound Diagnostics and Interventional Sonography, Kyiv, Ukraine; Association “Health and Wealth”, Kyiv, Ukraine; TSBUA “IT Solutions”, Kyiv, Ukraine; Maastricht University Medical Centre, Maastricht, The Netherlands; Fleischhacker GmbH, Schwerte, Germany; The European Association for Predictive, Preventive and Personalised Medicine, Brussels, Belgium; RIMS bvba, Overijse, Belgium; German Foundation For the Chronically Ill, Fürth, Germany; Linköping University, Norrköping, Sweden; Medical Faculty Pilsen, Pilsen, Czech Republic; Sananet Care BV, Sittard, The Netherlands; Exploris AG, Zürich, Switzerland; sense.ly, San Francisco, USA; Mulimedica SPA, Milano, Italy; Medtronic Iberica SA, Madrid, Spain; Maastricht University Medical Centre, Maastricht, The Netherlands; Clinical Hospital “Pheophania” of State Affairs Department, Zabolotny Str., 21, Kyiv, 03680 Ukraine; Zabolotny Institute of Microbiology and Virology, National Academy of Sciences of Ukraine, 154, Zabolotny st., Kyiv, 03680 Ukraine; Centre of Ultrasound Clinical Hospital “Pheophania” of State Affairs Department, Zabolotny Str., 21, Kyiv, 03680 Ukraine; Zabolotny Institute of Microbiology and Virology, National Academy of Sciences of Ukraine, 154, Zabolotny st., Kyiv, 03680 Ukraine; Endocrinology centre, Clinical Hospital “Pheophania” of State Affairs Department, Zabolotny Str., 21, Kyiv, 03680 Ukraine; Zabolotny Institute of Microbiology and Virology, National Academy of Sciences of Ukraine, Zabolotny Str., 154, Kyiv, 03680 Ukraine; Clinical Hospital ‘Pheophania’ of State Management of Affairs Department, Zabolotny Str., 21, Kyiv, 03680 Ukraine; Bogomolets National Medical University, T. Shevchenko boulevard, 13, Kyiv, 01601 Ukraine; Department of Physiology and Biochemistry, University of Malta, Msida, Malta; First Moscow State Medical University, Moscow, Russia; The Children’s Hospital of Philadelphia, Philadelphia, USA; Faculty of Physiotherapy, Wroclaw University School of Physical Education, Wroclaw, Poland; Inwestasekur, Wroclaw, Poland; VIANESSE AG, Research and Development, Engelberg, Switzerland; Department of Biomedical Engineering, Wroclaw University of Technology, Wroclaw, Poland; Center for Molecular Medicine, University Hospital Cologne, Cologne, Germany; Institut für Humangenetik, Universitätsklinikum Köln, Köln, Germany; Klinik und Poliklinik für Kinder- und Jugendmedizin, Universitätsklinikum Köln, Köln, Germany; Clinical Pharmacology and Pharmacogenetics Unit, Department of Clinical and Experimental Medicine, University of Pisa, Pisa, Italy; Medical Oncology Unit, University Hospital of Parma, Parma, Italy; Comparative Medicine, Messerli Research Institute of the University of Veterinary Medicine Vienna, Medical University Vienna and University Vienna, Vienna, Austria; Comparative Immunology and Oncology, Institute for Pathophysiology and Allergy Research, Center of Pathophysiology, Infectiology and Immunology, Medical University of Vienna, Vienna, Austria; Department for Companion Animals and Horses, University of Veterinary Medicine Vienna, Vienna, Austria; Laboratory of Veterinary Molecular Pathology and Therapeutics, Tokyo University of Agriculture and Technology, Tokyo, Japan; Division of Gastroenterology and Nutrition, Boston Children’s Hospital and Department of Pediatrics, Harvard Medical School, Boston, USA; Université de Lorraine, Metz, France; CHR Metz-Thionville, Pôle mère-enfant, Metz, France; Cassovia Life Sciences, Research and Innovation, Košice, Slovakia; Université Paris Descartes, Paris, France; Ecole Nationale Vétérinaire, Agroalimentaire et de l’Alimentation, Nantes, France; Université de Liège, Liège, Belgium; Luxembourg Institute of Sciences and Technology, Belvaux, Luxembourg; University of Catania, Catania, Italy; University Federico II of Naples, Naples, Italy; University of Messina, Messina, Italy; University College Cork, Cork, Ireland; Institut National de l’Environnement Industriel et des Risques, Paris, France; University Hospital, Rabat, Morocco; University of Rhodes Island, Rhodes Island, USA; Iowa State University, Ames, IA USA; Western University of Health Sciences, Oregon, USA; Biomedical Research Institute of Lleida, Lleida, Catalunya Spain; CEO - Owner at Intelligent Pharma, Barcelona, Spain; Laboratoires Réunis, Luxembourg, Luxembourg; Oregon Health & Science University, Oregon, USA; PAMIDA International, Kysucké Nové Mesto, Slovakia; University of Perm, Perm, Russia; European Drug Development Hub, Nancy, France; Université de Lille II Droit et Santé, Lille, France; Institute for Production and Work Environment Development, Quito, Ecuador; European Association for Predictive and Personalised Medicine, Brussels, Belgium; Stavropol State Medical University, Stavropol, Russia; Stavropol State Medical University, Stavropol, Russian Federation; Stavropol State Medical University, Stavropol, Russian Federation; Department of Radiology, University of Bonn, Bonn, Germany; Breast Cancer Research Centre, University of Bonn, Bonn, Germany; Clinical hospital “Pheophania” of State Affairs Department, Kyiv, Ukraine; University of Malta, Msida, Malta; Department of Ophthalmology, University of Basel, Basel, Switzerland; Department of Radiology, University of Bonn, Bonn, Germany; European Association for Predictive, Preventive and Personalised Medicine (EPMA), Brussels, Belgium; University Medical Center Groningen, Groningen, The Netherlands; European Medical Association (EMA), Brussels, Belgium; Department of Gynaecology and Obstetrics, University of Bonn, Bonn, Germany; Clinical Hospital ‘Pheophania’ of State Affairs Department, Zabolotny str., 21, Kyiv, 03680 Ukraine; Bogomolets National Medical University, Kyiv, 01601 Ukraine; Bogomolets National Medical University, Kyiv, 01601 Ukraine; Clinical Hospital ‘Pheophania’ of State Affairs Department, Zabolotny str., 21, Kyiv, 03680 Ukraine; Zabolotny Institute of Microbiology and Virology, National Academy of Sciences of Ukraine, Zabolotny Str., 154, Kyiv, 03680 Ukraine; St. Petersburg Periodontal Center “PAKS”, Pavlov First Saint Petersburg State Medical University, Saint Petersburg, Russia; Main Ukrainian Military Clinical Hospital #17 in Kyiv, Gospitalna str., 18, Kyiv, Ukraine; Kharkiv Medical Academy of Postgraduate Education, Kharkiv, Ukraine; Clinical Hospital “Pheophania” of State Affairs Department, Zabolotny Str., 21, Kyiv, 03680 Ukraine; Vision Sciences, School of Health and Life Sciences, Glasgow Caledonian University, Glasgow, Scotland UK; Cukurova University, Faculty of Dentistry, Adana, Turkey; Voronezh N.N. Burdenko State Medical University, Voronezh, Russia; Intercenter Urology Unit, Virgen de la Victoria University Hospital, Málaga, Spain; Myriad Genetics España S.L.U, Madrid, Spain; Intercenter Urology Unit, Virgen de la Victoria University Hospital, Málaga, Spain; Spezialklinik Neukirchen, Neukirchen beim Heiligen Blut, Germany; Donau University Krems, Krems, Austria; Department of Therapeutic Dentistry Medical University “Astrakhan State Medical Academy” Russian Ministry of Health, 121 Bakinskaya St., Astrakhan, 414000 Russia; Faculty of Dentistry Medical University “Voronezh State Medical Academy” of the Ministry of Health of the Russian Federation n.a. N.N. Burdenko, Institute of Dentistry at VGMA n.a. N.N. Burdenko, 14 pr. Revolyutsii, Voronezh, 394000 Russia; Department of Therapeutic Stomatology, Medical University “Astrakhan State Medical University” the Ministry of Health of Russia, 121 Bakinskaya St., Astrakhan, 414000 Russia; Faculty of Dentistry Medical University “Astrakhan State Medical Academy”, the Ministry of Health of Russia, Russia, 121 Bakinskaya St., Astrakhan, 414000 Russia; Augenzentrum Fankhauser, Bern, Switzerland; European Association for Predictive, Preventive and Personalised Medicine (EPMA), Brussels, Belgium; European Association for Predictive, Preventive and Personalised Medicine (EPMA), Brussels, Belgium; School of Art and Design, Nottingham Trent University, Nottingham, UK; Faculty Hospital in Pilsen, Charles University Medical Faculty in Pilsen, Pilsen, Czech Republic; Department of Medical and Life Sciences, Furtwangen University, Villingen-Schwenningen, Germany; Voronezh N.N. Burdenko State Medical University, Voronezh, Russia; Bogomolets National Medical University, Kyiv, 01601 Ukraine; Clinical Hospital ‘Pheophania’ of State Affairs Department, Zabolotny str., 21, Kyiv, 03680 Ukraine; Zabolotny Institute of Microbiology and Virology, National Academy of Sciences of Ukraine, Zabolotny Str., 154, Kyiv, 03680 Ukraine; Cassovia Life Science, Komenského 1337, 02401 Kysucké Nové Mesto, Slovakia; Institute of Transfusion Medicine and Immunology, Medical Faculty Mannheim, Heidelberg University, Mannheim, Germany; German Red Cross Blood Service Baden-Württemberg – Hessen, Mannheim, Germany; Laboratory for translational cellular and molecular biomedicine, Tomsk State University, Tomsk, Russia; National Research Tomsk Polytechnic University, Tomsk, Russia; Istyna-Veritas Research Center, Kyiv, Ukraine; Institute of Mathematics of NAS of Ukraine, Kyiv, Ukraine; Clinic of Healthy Vessels, Kyiv, Ukraine; Medical center “Ukrainian medical innovations”, Ternopil, Ukraine; Veritas IT Med Center for Innovative Medical Technologies, 4 Williams Str., Kyiv, 03191 Ukraine; Specialized clinic “Vessels, brain, neurorehabilitation”, Muscat, Oman; Department of Statistics and Operations Research, the Sackler Faculty of Exact Sciences, the Sagol School of Neurosciences, Tel Aviv University, Tel Aviv, Israel; Wroclaw University School of Physical Education, Faculty of Physiotherapy, Wroclaw, Poland; VIANESSE AG, Research and Development, Engelberg, Switzerland; Wroclaw University of Technology, Department of Biomedical Engineering, Wroclaw, Poland; Voronezh N.N. Burdenko State Medical University, Voronezh, Russia; Voronezh N.N. Burdenko State Medical University, Voronezh, Russia; Department of Oral Biology, Georgia Regents University, Augusta, Georgia 30912 USA; Department of Oral Biology, College of Dental Medicine, Georgia Regents University, Augusta, Georgia 30912 USA; Institute of Sports Medicine, Medical Faculty of Charles University, Plzeň, Czech Republic; Faculty of Applied Electronics, Western-Bohemian University, Plzeň, Czech Republic; Atrium-Orbis, Department of Clinical Chemistry and Haematology, Heerlen, The Netherlands; Department of Clinical Chemistry, University Hospital, SE-581 85 Linköping, Sweden; Therapeutic Dentistry Department, Pavlov First Saint Petersburg State Medical University of the Ministry of Health of the Russian Federation, Saint Petersburg, Russia; General and Clinical Psychology Department, Pavlov First Saint Petersburg State Medical University of the Ministry of Health of the Russian Federation, Saint Petersburg, Russia; Department of Clinical and Experimental Biomedical Sciences, University of Florence, Viale G. Pieraccini, 6, 50139 Florence, Italy; Department of Biochemistry Faculty of Health Sciences, University of Southern Denmark, Vejle Hospital, Vejle, Denmark; Clinical Department of Laboratory Medicine, University Hospitals Leuven, Leuven, Belgium; Department of Clinical Pharmacology, University Hospital Tuebingen, Tuebingen, Germany; Dr. Margarete Fischer-Bosch Institute of Clinical Pharmacology, Stuttgart, Germany; Department of Clinical Biochemistry, Faculty of Pharmacy, University of Ljubljana, Ljubljana, Slovenia; Department of Clinical Chemistry, Erasmus University Medical Centre, Rotterdam, The Netherlands; University of Lorraine, UMR ISERM U1122; IGE-PCV, Genetique Cardiovasculaire, Nancy, France; Division of Microbiology and Molecular Medicine, Department of Clinical, Experimental Medicine, Faculty of Health Sciences, Linköping University, Linköping, Sweden; Department of Clinical Chemistry, Center for Diagnostics, County Council of Östergötland, Linköping, Sweden; Genomic Unit for the Diagnosis of Human Pathologies, Vita-Salute San Raffaele University, Milan, Italy; Department of Electronics and Multimedia Communications, Faculty of Electrical Engineering and Informatics, Technical university of Kosice, Letná 9, Košice, Slovakia; Department of Electronics and Multimedia Communications, Faculty of Electrical Engineering and Informatics, Technical university of Kosice, Letná 9, Košice, Slovakia; Department of Histology and Embryology in Plzen, Charles University in Prague, Plzen, Czech Republic; Biomedical Centre, Faculty of Medicine in Plzen, Charles University in Prague, Plzen, Czech Republic; Department of Neurology, Faculty of Medicine in Plzen, Charles University in Prague and Faculty Hospital Plzen, Plzen, Czech Republic; Department of Investigational Cancer Therapeutics, The University of Texas MD Anderson Cancer Center, Houston, USA; Department of Biology, Faculty of Medicine in Plzen, Charles University in Prague and Faculty Hospital Plzen, Plzen, Czech Republic; Department of Surgery, Faculty of Medicine in Plzen, Charles University in Prague and Faculty Hospital Plzen, Plzen, Czech Republic; University Hospital Plzen and Faculty of Medicine in Plzen, Charles University in Prague, Department of Neurology, Plzen, Czech Republic; University Hospital Plzen, Department of Quality Assessment, Plzen, Czech Republic; University Clinic for Psychiatry and Psychotherapy, Paracelsus Private Medical University, Nuremberg, Germany; Georg Simon Ohm University for applied sciences, Nuremberg, Germany; Faculty for Medical Sciences, University Goce Delcev, Stip, Macedonia; Univesity Clinic for Psychiatry and Psychotherapy, Paracelsus Private Medical University, Nuremberg, Germany; Georg Simon Ohm University for applied sciences, Faculty for Social Sciences, Nuremberg, Germany; Faculty for Medical Sciences, University Goce Delcev, Stip, Macedonia; Clinic for Sleep Medicine, Bad Zurzcach, Schwitzerland; First Moscow State Medical University named by Sechenov, Moscow, Russia; European Association for Predictive, Preventive and Personalised Medicine, Brussels, Belgium; First Moscow State Medical University by I.M. Sechenov, Moscow, Russia; European Clinic of Sports Traumatology and Orthopaedics, Moscow, Russia; A.I.Evdokimov Moscow State University of Medicine & Dentistry, Moscow, Russia; Westdeutsches Zentrum für angewandte Telemedizin, Duisburg, Germany; Institute for Work and Technology, Gelsenkirchen, Germany; Institute for Nutrition Research, Beilinson Hospital, Rabin Medical Center, Petah Tikva, Israel; Ashkelon Academic College, Ashkelon, Israel; Voronezh N.N. Burdenko State Medical University, Voronezh, Russia; Voronezh State Medical University named after N.N. Burdenko, Postgraduate Dentistry Department, Voronezh, Russia; National Diabetes Committee and University Clinic of Emergency Internal Medicine and Toxicology, Skopje, Republic of Macedonia; National Diabetes Committee and University Clinic of Endocrinology, Diabetes and Metabolic Disorders, Skopje, Republic of Macedonia; Human Photosynthesis Study Center, Av. Aguascalientes Norte 607, Pulgas Pandas Sur, Aguascalientes México; I.M. Sechenov Firts Moscow State Medical University and A.I. Konstantinov Moscow State Medical & Dental University, Moscow, Russia; Department of Radiology, Univeristy of Bonn, Bonn, Germany; I.M. Sechenov First Moscow State Medical University (PMGMU), Moscow, Russia; A.I. Evdokimov Moscow State University of Medicine & Dentistry (MGMSU), Moscow, Russia; European Association for Predictive, Preventive and Personalised Medicine (EPMA), Brussels, Belgium; Department of Pharmaceutics, University of Florida College of Pharmacy, Gainesville, FL USA; Roche Diagnostics Ltd., Rotkreuz, Switzerland, and Roche Diagnostics Division, Basel, Switzerland; UCC (United Cultural Convention), Cambridge, UK; AMEE, Dundee, UK; New York Academy of Sciences, NY, USA; IKE/Klinisk kemi, Lab1, Hus 420, Plan 11, Universitetssjukhuset, SE-581 85, Linköping, Sweden; Department of Physics and Energy, and Materials and Surface Science Institute (MSSI), University of Limerick, ᅟ, Ireland; Faculty Hospital in Pilsen and Faculty of Medice in Pilsen, Charles University Prague, Pilsen, Czech Republic; General Faculty Hospital and 1st Faculty of Medice in Prague, Charles University Prague, Prague, Czech Republic; Clinical Hospital 86, Federal Medical Biological Agency of Russia, Moscow, Russia; I.M. Sechenov First Moscow State Medical University, Trubetskaya Str. 8-2, Moscow, 119991 Russia; A.I. Evdokimov Moscow State University of Medicine and Dentistry, Delegatskaya Str. 20/1, Moscow, 127473 Russia; Department of Clinical and Experimental Medicine, University Hospital of Catania, Catania, Italy; Department of Bio-Medical Sciences, Human Anatomy and Histology Section, School of Medicine, University of Catania, Catania, Italy; Proteomarkers Biotech P.C., Athens, Greece; Proteomics Research Unit, Biomedical research Foundation of the Academy of Athens, Athens, Greece; Proteomarkers Biotech P.C., Athens, Greece; Proteomics Research Unit, Biomedical research Foundation of the Academy of Athens, Athens, Greece; Medtronic Iberica S.A., Madrid, Spain; Telefonica, Madrid, Spain; Fleischhacker, Schwerte, Germany; Grand-Aides, Houston, Texas USA; I+ SRL, Firenze, Italy; Hospital G.U. Valencia, Valencia, Spain; Maastricht U. Medical Centre, Maastricht, The Netherlands; Linkopings Universitet, Linkopings, Sweden; Universidad Politecnica de Madrid, Madrid, Spain; European Association for Predictive, Preventive and Personalised Medicine, Brussels, Belgium; Hospital U. 12 de Octubre, Madrid, Spain; Elsevier, Amsterdam, The Netherlands; Medtronic Iberica S.A., Madrid, Spain; Universita Degli Studi di Padova, Padova, Italy; Universidad Politecnica de Madrid, Madrid, Spain; Universita Degli Studi di Pavia, Pavia, Italy; Fondazione Salvatore Maugeri Clinica del Lavoro e Della Riabilitazione, Pavia, Italy; Asociacion Espanola para el desarrollo de La Epidemiologia Clinica, Madrid, Spain; Lunds Universitet, Lund, Sweden; Soluciones Tecnologicas para la Salud y el Bienestar (TSB), Valencia, Spain; Samfundet Folkhalsan I Svenska Finland RF, Helsingfors, Finland; National Technical University of Athens, Athens, Greece; DiscoveryMed Ltd, St. Petersburg, Russia; Belarus State Medical University, Minsk, Belarus; DiscoveryMed Ltd, St. Petersburg, Russia; Belarus State Medical University, Minsk, Belarus; University of Tartu, Department of Stomatology, Tartu, Estonia; Clinic of Dentistry, Department of Maxillofacial Surgery, Tartu, Estonia; University of Tartu, Department of Internal Medicine, Tartu, Estonia; University of Tartu, Department of Pediatrics, Tartu, Estonia; School of Medical Science, Edith Cowan University, Perth, Australia; Municipal Key Laboratory of Clinical Epidemiology, Capital Medical University, Beijing, China; Global Health Epidemiology Reference Group (GHERG), Edinburgh, UK; School of Medical Science, Edith Cowan University, Perth, Australia; Municipal Key Laboratory of Clinical Epidemiology, Capital Medical University, Beijing, China; Global Health Epidemiology Reference Group (GHERG), Edinburgh, UK; Molecular Diagnostics, AIT- Austrian Institute of Technology GmbH, Vienna, Austria; Hacettepe University Center for Biobanking and Genomics, DNA/Cell Bank for Rare Diseases, Ankara, Turkey; Department of Medical Biology, Hacettepe University, Ankara, Turkey

## Abstract

A1 Predictive and prognostic biomarker panel for targeted application of radioembolisation improving individual outcomes in hepatocellular carcinoma

Jella-Andrea Abraham, Olga Golubnitschaja

A2 Integrated market access approach amplifying value of “Rx-CDx”

Ildar Akhmetov

A3 Disaster response: an opportunity to improve global healthcare

Russell J. Andrews, Leonidas Quintana

A4 USA PPPM: proscriptive, profligate, profiteering medicine-good for 1 % wealthy, not for 99 % unhealthy

Russell J. Andrews

A5 The role of IDO in a murine model of gingivitis: predictive and therapeutic potentials

Babak Baban, Jun Yao Liu, Xu Qin, Tailing Wang, Mahmood S. Mozaffari

A6 Specific diets for personalised treatment of diabetes type 2

Viktoriia V. Bati, Tamara V. Meleshko, Olga B. Levchuk, Nadiya V. Boyko

A7 Towards personalized physiotherapeutic approach

Joanna Bauer, Ewa Boerner, Halina Podbielska

A8 Cells, animal, SHIME and in silico models for detection and verification of specific biomarkers of non-communicable chronic diseases

Alojz Bomba, Viktor O. Petrov, Volodymyr G. Drobnych, Rostyslav V. Bubnov, Oksana M. Bykova, Nadiya V. Boyko

A9 INTERACT-chronic care model: Self-treatment by patients with decision support e-Health solution

Hans-Peter Brunner-La Rocca, Lutz Fleischhacker, Olga Golubnitschaja, Frank Heemskerk, Thomas Helms, Tiny Jaarsma, Judita Kinkorova, Jan Ramaekers, Peter Ruff, Ivana Schnur, Emilio Vanoli, Jose Verdu

A10 PPPM in cardiovascular medicine in 2015

Hans-Peter Brunner-La Rocca

A11 Magnetic resonance imaging of nanoparticles in mice, potential for theranostic and contrast media development – pilot results

Rostyslav V. Bubnov, Sergiy A. Grabovetskyi, Olena M. Mykhalchenko, Natalia O. Tymoshok, Oleksandr B. Shcherbakov, Igor P. Semeniv, Mykola Y. Spivak

A12 Ultrasound diagnosis for diabetic neuropathy - comparative study

Rostyslav V. Bubnov, Tetyana V. Ostapenko

A13 Ultrasound for stratification patients with diabetic foot ulcers for prevention and personalized treatment - pilot results

Rostyslav V. Bubnov, Nazarii M. Kobyliak, Nadiya M. Zholobak, Mykola Ya. Spivak

A14 Project ImaGenX – designing and executing a questionnaire on environment and lifestyle risk of breast cancer

John Paul Cauchi

A15 Genomics – a new structural brand of predictive, preventive and personalized medicine or the new driver as well?

Dmitrii Cherepakhin, Marina Bakay, Artem Borovikov, Sergey Suchkov

A16 Survey of questionnaires for evaluation of the quality of life in various medical fields

Barbara Cieślik, Agnieszka Migasiewicz, Maria-Luiza Podbielska, Markus Pelleter, Agnieszka Giemza, Halina Podbielska

A17 Personalized molecular treatment for muscular dystrophies

Sebahattin Cirak

A18 Secondary mutations in circulating tumour DNA for acquired drug resistance in patients with advanced ALK + NSCLC

Marzia Del Re, Paola Bordi, Valentina Citi, Marta Palombi, Carmine Pinto, Marcello Tiseo, Romano Danesi

A19 Recombinant species-specific FcεRI alpha proteins for diagnosis of IgE-mediated allergies in dogs, cats and horses

Lukas Einhorn, Judit Fazekas, Martina Muhr, Alexandra Schoos, Lucia Panakova, Ina Herrmann, Krisztina Manzano-Szalai, Kumiko Oida, Edda Fiebiger, Josef Singer, Erika Jensen-Jarolim

A20 Global methodology for developmental neurotoxicity testing in humans and animals early and chronically exposed to chemical contaminants

Arpiné A. Elnar, Nadia Ouamara, Nadiya Boyko, Xavier Coumoul, Jean-Philippe Antignac, Bruno Le Bizec, Gauthier Eppe, Jenny Renaut, Torsten Bonn, Cédric Guignard, Margherita Ferrante, Maria Liusa Chiusano, Salvatore Cuzzocrea, Gerard O'Keeffe, John Cryan, Michelle Bisson, Amina Barakat, Ihsane Hmamouchi, Nasser Zawia, Anumantha Kanthasamy, Glen E. Kisby, Rui Alves, Oscar Villacañas Pérez, Kim Burgard, Peter Spencer, Norbert Bomba, Martin Haranta, Nina Zaitseva, Irina May, Stéphanie Grojean, Mathilde Body-Malapel, Florencia Harari, Raul Harari, Kristina Yeghiazaryan, Olga Golubnitschaja, Vittorio Calabrese, Christophe Nemos, Rachid Soulimani

A21 Mental indicators at young people with attributes hypertension and pre-hypertension

Maria E. Evsevyeva, Elena A. Mishenko, Zurida V. Kumukova, Evgeniy V. Chudnovsky, Tatyana A. Smirnova

A22 On the approaches to the early diagnosis of stress-induced hypertension in young employees of State law enforcement agencies

Maria E. Evsevyeva, Ludmila V. Ivanova, Michail V. Eremin, Maria V. Rostovtseva

A23 Сentral aortic pressure and indexes of augmentation in young persons in view of risk factors

Maria E. Evsevyeva, Michail V. Eremin, Vladimir I. Koshel, Oksana V. Sergeeva, Nadesgda M. Konovalova

A24 Breast cancer prediction and prevention: Are reliable biomarkers in horizon?

Shantanu Girotra, Olga Golubnitschaja

A25 Flammer Syndrome and potential formation of pre-metastatic niches: A multi-centred study on phenotyping, patient stratification, prediction and potential prevention of aggressive breast cancer and metastatic disease

Olga Golubnitschaja, Manuel Debald, Walther Kuhn, Kristina Yeghiazaryan, Rostyslav V. Bubnov, Vadym M. Goncharenko, Ulyana Lushchyk, Godfrey Grech, Katarzyna Konieczka

A26 Innovative tools for prenatal diagnostics and monitoring: improving individual pregnancy outcomes and health-economy in EU

Olga Golubnitschaja, Jan Jaap Erwich, Vincenzo Costigliola, Kristina Yeghiazaryan, Ulrich Gembruch

A27 Immunohistochemical assessment of APUD cells in endometriosis

Vadym M. Goncharenko, Vasyl O. Beniuk, Olga V. Kalenska, Rostyslav V. Bubnov

A28 Updating personalized management algorithm of endometrial hyperplasia in pre-menopause women

Vadym M. Goncharenko, Vasyl O. Beniuk, Rostyslav V. Bubnov, Olga Melnychuk

A29 The personified treatment approach of polimorbid patients with periodontal inflammatory diseases

Irina A. Gorbacheva, Lyudmila Y. Orekhova, Vadim V. Tachalov

A30 Ukrainian experience in hybrid war – the challenge to update algorithms for personalized care and early prevention of different military injuries

Olena I. Grechanyk, Rizvan Ya. Abdullaiev, Rostyslav V. Bubnov

A31 Tear fluid biomarkers: a comparison of tear fluid sampling and storage protocols

Suzanne Hagan, Eilidh Martin, Ian Pearce, Katherine Oliver

A32 The correlation of dietary habits with gingival problems during menstruation

Cenk Haytac, Fariz Salimov, Servin Yoksul, Anatoly A. Kunin, Natalia S. Moiseeva

A33 Genomic medicine in a contemporary Spanish population of prostate cancer: our experience

Bernardo Herrera-Imbroda, Sergio del Río-González, Maria Fernanda Lara, Antonia Angulo, Francisco Javier Machuca Santa-Cruz

A34 Challenges, opportunities and collaborations for personalized medicine applicability in uro-oncological disease

Bernardo Herrera-Imbroda, Sergio del Río-González, Maria Fernanda Lara

A35 Metabolic hallmarks of cancer as targets for a personalized therapy

John Ionescu

A36 Influence of genetic polymorphism as a predictor of the development of periodontal disease in patients with gastric ulcer and 12 duodenal ulcer

Alfiya Z. Isamulaeva, Anatoly A. Kunin, Shamil Sh. Magomedov, Aida I. Isamulaeva

A37 Challenges in diabetic macular edema

Tatjana Josifova

A38 Overview of the EPMA strategies in laboratory medicine relevant for PPPM

Marko Kapalla, Juraj Kubáň, Olga Golubnitschaja, Vincenzo Costigliola

A39 EPMA initiative for effective organization of medical travel: European concepts and criteria

Vincenzo Costigliola, Marko Kapalla, Juraj Kubáň, Olga Golubnitschaja

A40 Design and innovation in e-textiles: implications for PPPM

Anthony Kent, Tom Fisher, Tilak Dias

A41 Biobank in Pilsen as a member of national node BBMRI_CZ

Judita Kinkorová, Ondřej Topolčan

A42 Big data in personalized medicine: hype and hope

Matthias Kohl

A43 The 3P approach as the platform of the European Dentistry Department (DPPPD)

Anatoly A. Kunin, Natalia S. Moiseeva

A44 The endometrium cytokine patterns for predictive diagnosis of proliferation severity and cancer prevention

Andrii I. Kurchenko, Vasyl A. Beniuk, Vadym M. Goncharenko, Rostyslav V. Bubnov, Nadiya V. Boyko, Andriy M. Strokan

A45 A monocyte-based in-vitro system for testing individual responses to the implanted material: future for personalized implant construction

Julia Kzhyshkowska, Alexandru Gudima, Ksenia S. Stankevich, Victor D. Filimonov^4^, Harald Klüter, Evgeniya M. Mamontova, Sergei I. Tverdokhlebov

A46 Prediction and prevention of adverse health effects by meteorological factors: Biomarker patterns and creation of a device for self-monitoring and integrated care

Ulyana B. Lushchyk, Viktor V. Novytskyy, Igor P. Babii, Nadiya G. Lushchyk, Lyudmyla S. Riabets, Ivanna I. Legka

A47 Targeting "disease signatures" towards personalized healthcare

Mira Marcus-Kalish, Alexis Mitelpunkt, Tal Galili, Neta Shachar, Yoav Benjamini

A48 Influence of the skin imperfection on the personal quality of life and possible tools for objective diagnosis

Agnieszka Migasiewicz, Markus Pelleter, Joanna Bauer, Ewelina Dereń, Halina Podbielska

A49 The new direction in caries prevention based on the ultrastructure of dental hard tissues and filling materials

Natalia S. Moiseeva, Anatoly A. Kunin, Dmitry A. Kunin

A50 The use of LED radiation in prevention of dental diseases

Natalia S. Moiseeva, Yury A. Ippolitov, Dmitry A. Kunin, Alexei N. Morozov, Natalia V. Chirkova, Nakhid T. Aliev

A51 Status of endothelial progenitor cells in diabetic nephropathy: predictive and preventive potentials

Mahmood S. Mozaffari, Jun Yao Liu, Babak Baban

A52 The status of glucocorticoid-induced leucine zipper protein in salivary gland in Sjögren’s syndrome: predictive and personalized treatment potentials

Mahmood S. Mozaffari, Jun Yao Liu, Rafik Abdelsayed, Xing-Ming Shi, Babak Baban

A53 Maximal aerobic capacity - important quality marker of health

Jaroslav Novák, Milan Štork, Václav Zeman

A54 The EMPOWER project: laboratory medicine and Horizon 2020

Wytze P. Oosterhuis, Elvar Theodorsson

A55 Personality profile manifestations in patient’s attitude to oral care and adherence to doctor’s prescriptions

Lyudmila Y. Orekhova, Tatyana V. Kudryavtseva, Elena R. Isaeva, Vadim V. Tachalov, Ekaterina S. Loboda

A56 Results of an European survey on personalized medicine addressed to directions of laboratory medicine

Mario Pazzagli, Francesca Malentacchi, Irene Mancini, Ivan Brandslund, Pieter Vermeersch, Matthias Schwab, Janja Marc, Ron H.N. van Schaik, Gerard Siest, Elvar Theodorsson, Chiara Di Resta

A57 MCI or early dementia predictive speech based diagnosis techniques

Matus Pleva, Jozef Juhar

A58 Personalized speech based mobile application for eHealth

Matus Pleva, Jozef Juhar

A59 Circulating tumor cell-free DNA as the biomarker in the management of cancer patients

Jiří Polívka jr., Filip Janků, Martin Pešta, Jan Doležal, Milena Králíčková, Jiří Polívka

A60 Complex stroke care – educational programme in Stroke Centre University Hospital Plzen

Jiří Polívka, Alena Lukešová, Nina Müllerová, Petr Ševčík, Vladimír Rohan

A61 Sleep apnea and sleep fragmentation contribute to brain aging

Kneginja Richter, Lence Miloseva, Günter Niklewski

A62 Personalised approach for sleep disturbances in shift workers

Kneginja Richter, Jens Acker, Guenter Niklewski

A63 Medical travel and innovative PPPM clusters: new concept of integration

Olga Safonicheva, Vincenzo Costigliola

A64 Medical travel and women health

Olga Safonicheva

A65 Continuity of generations in the training of specialists in the field of reconstructive microsurgery

Maxim Sautin, Janna Sinelnikova, Sergey Suchkov

A66 Telemonitoring of stroke patients – empirical evidence of individual risk management results from an observational study in Germany

Songül Secer, Stephan von Bandemer

A67 Women’s increasing breast cancer risk with n-6 fatty acid intake explained by estrogen-fatty acid interactive effect on DNA damage: implications for gender-specific nutrition within personalized medicine

Niva Shapira

A68 Cytobacterioscopy of the gingival crevicular fluid as a method for preventive diagnosis of periodontal diseases

Aleksandr Shcherbakov, Anatoly A. Kunin, Natalia S. Moiseeva

A69 Use of specially treated composites in dentistry to avoid violations of aesthetics

Bogdan R. Shumilovich, Zhanna Lipkind, Yulia Vorobieva, Dmitry A. Kunin, Anastasiia V. Sudareva

A70 National eHealth system – platform for preventive, predictive and personalized diabetes care

Ivica Smokovski, Tatjana Milenkovic

A72 The common energy levels of Prof. Szent-Györgyi, the intrinsic chemistry of melanin, and the muscle physiopathology. Implications in the context of Preventive, Predictive, and Personalized Medicine

Arturo Solís-Herrera, María del Carmen Arias-Esparza, Sergey Suchkov

A73 Plurality and individuality of hepatocellular carcinoma: PPPM perspectives

Krishna Chander Sridhar, Olga Golubnitschaja

A74 Strategic aspects of higher medical education reforms to secure newer educational platforms for getting biopharma professionals matures

Maria Studneva, Sihong Song, James Creeden, Мark Мandrik, Sergey Suchkov

A75 Overview of the strategies and activities of the European Federation of Clinical Chemistry and Laboratory Medicine, (EFLM)

Elvar Theodorsson, EFLM

A76 New spectroscopic techniques for point of care label free diagnostics

Syed A. M. Tofail

A77 Tumor markers for personalized medicine and oncology - the role of Laboratory Medicine

Ondřej Topolčan, Judita Kinkorová, Ondřej Fiala, Marie Karlíková, Šárka Svobodová, Radek Kučera, Radka Fuchsová, Vladislav Třeška, Václav Šimánek, Ladislav Pecen, Jan Šoupal, Štěpán Svačina^2^

A78 Modern medical terminology (MMT) as a driver of the global educational reforms

Evgeniya Tretyak, Maria Studneva, Sergey Suchkov

A79 Juvenile hypertension; the relevance of novel predictive, preventive and personalized assessment of its determinants

Francesca M. Trovato, G. Fabio Martines, Daniela Brischetto, Daniela Catalano, Giuseppe Musumeci, Guglielmo M. Trovato

A80 Proteomarkers Biotech

George Th. Tsangaris, Athanasios K. Anagnostopoulos

A81 Proteomics and mass spectrometry based non-invasive prenatal testing of fetal health and pregnancy complications

George Th. Tsangaris, Athanasios K. Anagnostopoulos

A82 Integrated Ecosystem for an Integrated Care model for Heart Failure (HF) patients including related comorbidities (ZENITH)

José Verdú, German Gutiérrez, Jordi Rovira, Marta Martinez, Lutz Fleischhacker, Donna Green, Arthur Garson, Elena Tamburini, Stefano Cuomo, Juan Martinez-Leon, Teresa Abrisqueta, Hans-Peter Brunner-La Rocca, Tiny Jaarsma, Teresa Arredondo, Cecilia Vera, Giuseppe Fico, Olga Golubnitschaja, Fernando Arribas, Martina Onderco, Isabel Vara, on behalf of ZENITH consortium

A83 Predictive, preventive and personalized medicine in diabetes onset and complication (MOSAIC project)

José Verdú, Francesco Sambo, Barbara Di Camillo, Claudio Cobelli, Andrea Facchinetti, Giuseppe Fico, Riccardo Bellazzi, Lucia Sacchi, Arianna Dagliati, Daniele Segnani, Valentina Tibollo, Manuel Ottaviano, Rafael Gabriel, Leif Groop, Jacqueline Postma, Antonio Martinez, Liisa Hakaste, Tiinamaija Tuomi, Konstantia Zarkogianni, on behalf of MOSAIC consortium

A84 Possibilities for personalized therapy of diabetes using *in vitro* screening of insulin and oral hypoglycemic agents

Igor Volchek, Nina Pototskaya, Andrey Petrov

A85 The innovative technology for personalized therapy of human diseases based on *in vitro* drug screening

Igor Volchek, Nadezhda Pototskaya, Andrey Petrov

A86 Bone destruction and temporomandibular joint: predictive markers, pathogenetic aspects and quality of life

Ülle Voog-Oras, Oksana Jagur, Edvitar Leibur, Priit Niibo, Triin Jagomägi, Minh Son Nguyen, Chris Pruunsild, Dagmar Piikov, Mare Saag

A87 Sub-optimal health management – global vision for concepts in medical travel

Wei Wang

A88 Sub-optimal health management: synergic PPPM-TCAM approach

Wei Wang

A89 Innovative technologies for minimal invasive diagnostics

Andreas Weinhäusel, Walter Pulverer, Matthias Wielscher, Manuela Hofner, Christa Noehammer, Regina Soldo, Peter Hettegger, Istvan Gyurjan, Ronald Kulovics, Silvia Schönthaler, Gabriel Beikircher, Albert Kriegner, Stephan Pabinger, Klemens Vierlinger

A90 Rare disease diobanks for personalized medicine

Ayşe Yüzbaşıoğlu, Meral Özgüç, Member of EuroBioBank - European Network of DNA, Cell and Tissue Banks for Rare Diseases

## A1 Predictive and prognostic biomarker panel for targeted application of radioembolisation improving individual outcomes in hepatocellular carcinoma

### Jella-Andrea Abraham, Olga Golubnitschaja

#### Department of Radiology, University of Bonn, Sigmund-Freud-Str. 25, 53105 Bonn, Germany

##### **Correspondence:** Olga Golubnitschaja (olga.golubnitschaja@ukb.uni-bonn.de) – Department of Radiology, University of Bonn, Sigmund-Freud-Str. 25, 53105 Bonn, Germany

**Keywords:** Liver tumour, Hepatocellular carcinoma, Radioembolisation, Radiosensitivity, Patient stratification, Individual patient profiles, Biomarker panel, Multilevel diagnostics, Multi-omics, Predictive preventive personalized medicine

Liver cancer is the fifth most common form of cancer worldwide [1], with an incidence rate almost equals the mortality rate and ranks 3^rd^ among causes of cancer related death [2]. The coexistence of two life threatening conditions, cancer and liver cirrhosis makes the staging challenging. However, there are some staging systems, e.g. the Barcelona staging system for Hepatocellular carcinoma (HCC) [3], that suggest treatment options and management. Whereas diagnosis in early stages gives hope for a curative outcome, the treatment regime for around 80 % [2] of the patients classified as severe stages only gears towards palliation [4]. An intra-arterial radiation approach, radioembolisation (RE) is ubiquitously applied as one of palliative approaches. Although, in general RE shows promising results in intermediate and advanced stage HCC [5], individual treatment outcomes are currently unpredictable. Corresponding stratification criteria are still unclear.

We hypothesised that individual radioresistance/radiosensitivity may play a crucial role in treatment response towards RE strongly influencing individual outcomes. Further, HCC represents a highly heterogeneous group of patients which requires patient stratification according to clear criteria for treatment algorithms to be applied individually. Multilevel diagnostic approach (MLDA) is considered helpful to set-up optimal predictive and prognostic biomarker panel for individualised application of radioembolisation. Besides comprehensive medical imaging, our MLDA includes non-invasive multi-omics and sub-cellular imaging. Individual patient profiles are expected to give a clue to targeting shifted molecular pathways, individual RE susceptibility, treatment response. Hence, a dysregulation of the detoxification pathway (SOD2/Catalase) might indicate possible adverse effects of RE, and highly increased systemic activities of matrix metalloproteinases indicate an enhanced tumour aggressiveness and provide insights into molecular mechanisms/targets. Consequently, an optimal set-up of predictive and prognostic biomarker panels may lead to the changed treatment paradigm from untargeted “treat and wait” to the cost-effective predictive, preventive and personalised approach, improving the life quality and life expectancy of HCC patients.

**References**

1. Li QL, Zhang PP, Wang PQ, Yu HB, Sun F, Hu WZ, et al. The cytotoxic and mechanistic effects of aaptamine on hepatocellular carcinoma. Anticancer Agents Med Chem. 2015;15(3):291–7.

2. Li Q, Hu Y, Xi M, He L, Zhao L, Liu M. Sorafenib modulates the radio sensitivity of hepatocellular carcinoma cells in vitro in a schedule-dependent manner. BMC Cancer. 2012;12:485.

3. Llovet JM, Ducreux M, Lencioni R, Di Bisceglie AM, Galle PR, Dufour JF, et al. EASL-EORTC clinical practice guidelines: management of hepatocellular carcinoma. J Hepatol. 2012;56(4):908–43.

4. Berliner L, Lemke HU (eds). An Information Technology Framework for Predictive, Preventive and Personalised Medicine: A Use-Case with Hepatocellular Carcinoma vol. 8. In Book Series “Advances in Predictive, Preventive and Personalised Medicine”, Golubnitschaja O. (Series Editor), Springer Dordrecht Heidelberg New York London, ISBN 978-3-319-12165-9, 2015.

5. Mazzaferro V, Sposito C, Bhoori S, Romito R, Chiesa C, Morosi C, et al. Yttrium-90 radioembolization for intermediate-advanced hepatocellular carcinoma: a phase 2 study. Hepatology. 2013;57(5):1826–37.

## A2 Integrated market access approach amplifying value of “Rx-CDx”

### Ildar Akhmetov (ildar.export@gmail.com )

#### Market Access at Unicorn, P.O.B. 91, Zhytomyr 10020, Ukraine

**Keywords:** Market access, Value, Strategy, Companion diagnostics, Cost-effectiveness, Reimbursement, Health technology assessment, Economic models, Predictive preventive personalized medicine

Achieving and sustaining seamless “drug – companion diagnostic” market access requires a sound strategy throughout a product life cycle, which enables timely creation, substantiation and communication of value to key stakeholders [1, 2]. The study aims at understanding the root-cause of market access inefficiencies of companies by gazing at the “Rx-CDx” co-development process through the prism of “value”, and developing a perfect co-development scenario based on the literature review and discussions with the subject matter experts. The presenter suggests that an integrated market access approach is the need of the hour, and it should cover the entire “Rx-CDx” value chain – from early-stage pre-clinical (Rx) and feasibility (CDx) studies to post-launch considerations of a co-labeled product [3]. Such approach can leverage patient selection strategies to reduce clinical study size, increase chances to achieve earlier regulatory submission and launches, contribute to better upside for drug developers, simplify value justification, fit with the emerging “value-based” healthcare delivery practices, enable risk sharing, and facilitate funding of the biomarker research [1, 3, 4].

**References**

1. Akhmetov I, Bubnov RV. Assessing value of innovative molecular diagnostic tests in the concept of predictive, preventive, and personalized medicine. EPMA J. 2015;6(19):12. doi: 10.1186/s13167-015-0041-3

2. Agarwal A, Ressler D, Snyder G. The current and future state of companion diagnostics. Pharmgenomics Pers Med. 2015;8:99-110. doi: 10.2147/PGPM.S49493

3. Akhmetov IR, Ramaswamy R, Akhmetov IR, Thimmaraju, P. Market access advancements and challenges in “drug-companion diagnostic test” co-development in Europe. J Pers Med. 2015;5(2):213-28. doi:10.3390/jpm5020213

4. Olsen D, Jorgensen JT. Companion diagnostics for targeted cancer drugs - clinical and regulatory aspects. Front Oncol. 2014;4:105. doi: 10.3389/fonc.2014.00105

## A3 Disaster response: an opportunity to improve global healthcare

### Russell J. Andrews^1^, Leonidas Quintana^2^

#### ^1^Nanotechnology & Smart Systems, NASA Ames Research Center, Moffett Field, CA, USA; ^2^Department of Neurosurgery, Valparaiso University Medical School, Valparaiso, Chile

##### **Correspondence:** Russell J. Andrews (rja@russelljandrews.org) – Nanotechnology & Smart Systems, NASA Ames Research Center, Moffett Field, CA, USA

**Keywords:** Disaster response, Emergency response, Global health care, International medicine, Medical evacuation, Mobile hospitals, Trauma, Telemedicine

**Objectives:** The United Nations estimates in the past two decades disasters have cost over 1.3 million lives and US $2 trillion damage. During the first week following the 2010 Haiti earthquake, an estimated *20,000 people died each day* due to lack of surgical facilities [1]. To improve survival, disaster response resources must be “on-site” within 24 hours - not the days to weeks currently accepted [2].

**Approaches:** Trauma and stroke centers (TSCs) evolved when evidence showed that immediate “24/7” treatment resulted in dramatically improved morbidity/mortality [3, 4]. TSCs are part of the “mainstream” ongoing healthcare delivery and education system - not a separate entity. All TSC physicians, nurses, and personnel (both senior and in-training) are seamlessly integrated into the overall healthcare system. Equipment is available (e.g. operating rooms and battery-driven CTs, portable by helicopter) for a mobile trauma center to be operational anywhere worldwide less than 24 hours after a disaster strikes. Fortunately the universal humanitarian response to disasters removes political, cultural, and socioeconomic barriers that hinder the response to other global medical problems.

**Results:** Disaster response remains separate from ongoing healthcare systems, e.g. UNOCHA (UN Office for Coordination of Humanitarian Affairs) and the Red Cross. We propose that disaster response - like TSCs - be integrated into ongoing healthcare systems worldwide (governmental/nongovernmental, national/international) [2]. This global “mega TSC system” would improve disaster response and, moreover, establish worldwide training, certification, and research standards for healthcare.

**Recommendations:** There are substantial political, cultural, and socioeconomic benefits - in addition to healthcare benefits - of integrating disaster response globally into the ongoing healthcare system.

**References**

1. CBS News 60 Minutes 18 Jan 2010. @katiecouric: Disaster in Haiti. Available from: http://www.cbsnews.com/videos/katiecouric-disaster-in-haiti/. Accessed March 30, 2015.

2. Andrews RJ, Quintana LM. Unpredictable, unpreventable and impersonal medicine: global disaster response in the 21^st^ century. EPMA J. 2015;6(2):1-12.

3. Haas B, Stukel TA, Gomez D, Zagorski B, De Mestral C, Sharma SV, et al. The mortality benefit of direct trauma center transport in a regional trauma system: a population-based analysis. J Trauma Acute Care Surg. 2012;72:1510–5.

4. Meretoja A, Roine RO, Kaste M, Linne M, Roine S, Juntunen M, et al. Effectiveness of primary and comprehensive stroke centers – PERFECT Stroke: a nationwide observational study from Finland. Stroke. 2010;41:1102–7.

## A4 USA PPPM: proscriptive, profligate, profiteering medicine-good for 1 % wealthy, not for 99 % unhealthy

### Russell J. Andrews (rja@russelljandrews.org) 

#### Nanotechnology & Smart Systems, NASA Ames Research Center, Moffett Field, CA, USA

**Keywords:** For-profit medicine, Healthcare financing, Medical education, Universal healthcare

**Objectives:** USA spends twice other developed countries on healthcare, yet lags far behind. Its near neighbor, Cuba, spends one-tenth *per capita* compared to USA, yet matches or exceeds on life expectancy and infant mortality [1]. Can USA healthcare merely rein in excessive costs (as healthcare economists assert), or is a fundamental change necessary?

**Approaches:** USA healthcare embraces the profit incentive, and the necessity for healthcare insurance. Despite its title, the Affordable Care Act (ACA – aka “Obamacare”) has not reduced healthcare costs [2]. The massive healthcare lobby in Washington DC and state capitals – more than three times the defense lobby financially – ensures that those parties doing well under the current system will continue to do well. Opposition to the present system is met with “socialized medicine”, a term commonly equated with “un-American”.

**Results:** Under ACA, costs shifted from insurer to patient: increasing deductibles ($5,000/year or more) and co-pays (up to $75 per outpatient visit) make it cheaper for US citizens to travel to Europe for surgical procedures (paying cash!) than pay deductibles/co-pays in the USA [1, 2]. A primary physician is reimbursed $16 for a patient covered by Medi-Cal (the California coverage for the uninsured) – ¼ the co-pay under some insurance plans. Residents/registrars now spend time learning “coding” – classification of medical care to maximize hospital and physician reimbursement – rather than medical science or patient care [1, 3]. The hospitalist model allows convenient “shift work” medicine, but degrades continuity – essential for efficient healthcare. Economists manipulate statistics to demonstrate incremental financial advantages of “tweaking” USA healthcare – which fail more comprehensive analyses.

**Recommendations:** Despite the political and lobbyist inertia, USA physicians unanimously recommend universal healthcare (like all other developed countries) [1, 4]. In education – lacking rich lobbyists - “commercial” (for-profit) colleges have recently declared bankruptcy (Corinthian Colleges) or been charged with stock-fraud (ITT Educational Services). They profited from government-backed student loans but - with meaningless diplomas - graduates remained unemployed, defaulting on loans. A prime example of “profits are privatized, but losses are socialized” – is also true in USA healthcare [1]. Until USA realizes that for-profit healthcare cannot be “too big to fail or jail”, meaningful healthcare reform will remain elusive. Learn from USA healthcare!

**References**

1. Andrews RJ. Too big to succeed: profiteering in American medicine. iUniverse, 2013.

2. Alonzo-Zaldivar R. Skimpy insurance seen by Democrats as next health care issue. Associated Press, May 25, 2015.

3. Ludmerer KM. Let me heal: the opportunity to preserve excellence in American medicine. Oxford University Press, 2014.

4. Relman AS. The new medical-industrial complex. N Engl J Med. 1980;303:963-70.

## A5 The role of IDO in a murine model of gingivitis: predictive and therapeutic potentials

### Babak Baban, Jun Yao Liu, Xu Qin, Tailing Wang, Mahmood S. Mozaffari

#### Department of Oral Biology, College of Dental Medicine, Georgia Regents University, Augusta, Georgia 30912, USA

##### **Correspondence:** Babak Baban (BBABAN@gru.edu) – Department of Oral Biology, College of Dental Medicine, Georgia Regents University, Augusta, Georgia 30912, USA

**Keywords:** Indoleamine 2,3-dioxygenase, Gingivitis, Regulatory T cells, Cytokines, Cell death, Predictive and therapeutic potentials

**Scientific Objective:** Indoleamine 2,3 dioxygenase (IDO) is a cytosolic enzyme and a major pathway for metabolism of tryptophan in a variety of cells including immune cells. Increasing evidence indicates that IDO is a critical player in establishing the balance between immunity and tolerance and ultimately maintenance of normal homeostasis. Gingivitis is a prevalent human condition with the hallmark feature of severe inflammation but the role of IDO in this condition remains elusive. Thus, this study tested the hypothesis that IDO is a pivotal player in regulating the immune and inflammatory responses of gingiva.

**Technical Approach/Methods:** We utilized the IDO knockout mouse model in conjunction with lipopolysaccharide (LPS)-induced gingivitis. Accordingly, wild type and IDO knockout mice were injected with LPS in the anterior mandible followed by procurement of gingival tissue for histopathology and preparation of tissue for flow cytometry-based studies.

**Results/Interpretation:** Clinical and histological examinations revealed marked adverse impact of IDO deficiency on LPS-induced gingivitis. These observations were consistent with a marked increase in the pro-inflammatory cytokine interleukin (IL)-17 positive cells but no significant change in the anti-inflammatory IL-10 positive cells. Consistent with the more marked pro-inflammatory impact of IDO deficiency, the percent of regulatory T cells (Tregs) was markedly lower in gingival tissue of IDO knockout than wild type mice. These pro-inflammatory changes were accompanied with marked increase in apoptotic and necrotic cell death in gingival tissue of IDO knockout than wild type mice.

**Outlook/Expert recommendations:** Severe inflammation of periodontal soft tissue is a hallmark feature of gingivitis. If left untreated, it can progress to destruction of alveolar bon, ultimately causing mobility and loss of one or more teeth. Thus, effective treatment modalities emanating from the pathogenesis of gingivitis as well as the possibility of predicting gingivitis lesions with greater propensity to progress to periodontitis would be of clinical relevance and significance. Our studies have established the role of IDO in pathogenesis of gingivitis thereby laying down the foundation to further explore and harness its predictive and treatment potentials for diseases of periodontal tissues.

## A6 Specific diets for personalised treatment of diabetes type 2

### Viktoriia V. Bati (v.bati@mail.ru)^1^, Tamara V. Meleshko (meleshkotv@ukr.net)^1^, Olga B. Levchuk (olgalevchuk27@gmail.com)^1,2^, Nadiya V. Boyko^1,3^

#### ^1^Research, Developing and Training Centre of Molecular Microbiology and Mucosal Immunology, Uzhhorod National University, Uzhhorod, Ukraine; ^2^“Astra-DIA” Medical Diagnostic Centre, Uzhhorod, Ukraine; ^3^Cassovia Life Science, Kysucké Nové Mesto, Slovakia

##### **Correspondence:** Nadiya V. Boyko (nadiya.boyko@cassovialifesciences.eu) – Cassovia Life Science, Kysucké Nové Mesto, Slovakia

**Keywords:** Personalised patient-centred nutrition, Chronic diseases, Human gut, Saliva microbiome

**Scientific objective:** To verify via limited clinical trial the efficacy of personalised nutrition proposed for the treatment of patients with diabetes type 2 (DT2).

**Technological approaches**: 1) screening of patient-centred diets based on detection of each person saliva and gut microbiome profile (SGM) and on previous data obtained in vitro and in vivo experiments about ability of plant originated foods and selected fermented traditional products’ to regulate specifically the ratio between commensal and opportunistic representatives of human SGM; 2) limited clinical diet experiment for randomly assigned for classical antidiabetic therapy and patients fed additionally with person-selected local ethnical foods, with chemically analysed [1] and balanced composition of biologically active compounds and defined by MALDI and gene sequencing technique microbial content.

**Results interpretation:** Specific SGM profile of each patient had been indicated before and after applied diet. Implementation of control diet led to the normalisation of the majority of SGM representatives. Increasing number of *Lactobacillus* spp. was accompanied by reduced levels of glycosylated haemoglobin. Reduction of cholesterol and triglycerides in the blood of patients had been correlated with the decreased amount of *Enterococcus spp.* in gut microbiota content. Normalised glucose and improved ratio between low and high density lipids had been reported.

**Outlook and Expert recommendations:** Chronic inflammation led to the development of human metabolic non-communicable disorders. Earlier we had proposed alarm markers for diagnostic of DT2 and cardiovascular diseases [2].

This study recommends to develop software/in silico model in order to apply widely obtained algorithm of personalised nutrition approach for patient-centred treatment for other chronic diseases connected with human microbiome disbalance.

**References**

1. Costa H, Albuquerque T, Sanches-Silva A, Vasilopoulou E, Trichopoulou A, D'Antuono F, et al. New nutritional composition data on selected traditional foods consumed in Black Sea Area countries. J Sci Food Agric. 2013,93:3524–34.

2. Petrov VO, Boyko NV. Early diagnostic markers of obesity, diabetes and metabolic syndrome. Patent number 90788: Appl. 08/01/2014, publ. 10.06.2014 Bull. №11.

## A7 Towards personalized physiotherapeutic approach

### Joanna Bauer^1^, Ewa Boerner^2^, Halina Podbielska^1,2^

#### ^1^Wroclaw University of Technology, Department of Biomedical Engineering, Wroclaw, Poland; ^2^Wrocław University School of Physical Education, Faculty of Physiotherapy, Wroclaw, Poland

##### **Correspondence:** Halina Podbielska (info@halinapodbielska.pl) – Wrocław University School of Physical Education, Faculty of Physiotherapy, Wroclaw, Poland

**Keywords:** Physiotherapy, Personalized treatment, Thermal imaging

**Scientific objectives:** In spite of a wide bibliography concerning physiotherapy, there are still no comprehensive studies on the influence of patient’s age, gender, BMI, dose of therapeutic agent, treatment’s duration and application’s order in the therapy outcomes. Personalization of physical treatment is not a trivial task; it requires a proper planning of procedures, especially in case of multiple therapies. Monitoring of the changes due to various stimuli is essential for proper planning. Thermal imaging enables the assessment of body reaction and can be exploited in the physical medicine. The main objective of our study is to compare the polytherapy results depending of the order of administration as well as individual parameters. This study was performed with the formal approval of the Senate Committee for the Ethics of Scientific Research of the Wroclaw University School of Physical Education.

**Technological approaches:** Thermographic recording was used to estimate the thermal effects of the physical treatments. Various physical stimuli were applied, as electric current, ultrasound, thermal agents and cryogenic cooling in various orders. The study was conducted on the healthy volunteers. The influence of the multiple therapies sequence, as well age and BMI of treated individuals was evaluated.

All volunteers involved in the study fulfilled the inclusion criteria and were informed about the risks and benefits of the procedures. Their consent was obtained, and all participants were familiarized with the study's purpose and procedures.

**Results interpretation:** Our observations revealed that age should be taken as a premise to personalization of the treatment parameters. The elderly persons usually react more exhaustive on physical stimuli and this reaction lasts longer. Also the order of physical treatments in case of polytherapy is important and can cause significant temperature changes in the treated area.

**Outlook and Expert recommendations:** Monitoring of the thermal response to various physical agents may help to plan physical treatments more precisely. The further studies are necessary to establish the optimal therapies, parameters and sequences, depending on the individual responses of the treated individuals.

## A8 Cells, animal, SHIME and in silico models for detection and verification of specific biomarkers of non-communicable chronic diseases

### Alojz Bomba (alojz.bomba@upjs.sk)^1,2^, Viktor O. Petrov (petrovviktor.uzh@gmail.com)^3^, Volodymyr G. Drobnych (drobnich@rambler.ru)^3^, Rostyslav V. Bubnov (rostbubnov@gmail.com)^4^, Oksana M. Bykova (oksana.bykova@tsbua.com)^5,6^, Nadiya V. Boyko^2,3,5^

#### ^1^Pavol Jozef Šafárik University, Košice, Slovakia; ^2^Cassovia Life Science, Kysucké Nové Mesto, Slovakia; ^3^Uzhhorod National University, Uzhhorod, Ukraine; ^4^The Centre of ultrasound diagnostics and interventional sonography, Clinical hospital “Pheophania” of State Affairs Department, Kyiv, Ukraine; ^5^Association “Health and Wealth”, Kyiv, Ukraine; ^6^TSBUA “IT Solutions”, Kyiv, Ukraine

##### **Correspondence:** Nadiya V. Boyko (nadiya.boyko@cassovialifesciences.eu) – Association “Health and Wealth”, Kyiv, Ukraine

**Keywords:** Patient stratification, Human microbiome, in vitro, in vivo, ex vivo, in silico models, Biomarkers.

**Scientific objectives**: Human microbiome data and the natural way of its correction needed to be exploited for implementation of patient stratification strategy.

Verification of *in vitro*, *in vivo*, *ex vivo*, *in silico* models and detected biomarkers of chronic diseases should be revised for realisation of new health care-system based on innovative personalized management of disease and health via creation of clinically and economically optimal diagnostics/treatment algorithms.

**Technological approaches:** 1) *in vitro* (human dendritic cells derived from peripheral blood monocytes, DCs), *in vivo* (mice/rats animal models), *ex vivo* (fragment culture technique) and *in silico* (data bases of limited cohort study) models for detection of strongly correlated biomarkers of initiation of low-grade inflammation including changes of saliva/gut microbiota (SGM) compositions, relevant molecular pathways, metabolic production and typical epigenetic factors; 2) integrated *in vitro* Simulator of Human Intestinal Microbial Ecosystem (SHIME) technology platform for verification of efficacy of proposed pre-screened natural ways of human microbiome correction and biomarkers validation.

**Results interpretation:** Chronic low-grade inflammation is the key linking factors of the metabolic and age relevant diseases. As results of limited cohort studies indicators for early diagnostic of the selected non-communicable diseases (obesity, diabetes type 2 and cardiovascular diseases) based on correlation between microbial, biochemical and immune parameters are proposed.

Healthy products in form of modified ethnical foods were designed and ability of their active components to effect on SGM, modulate specifically host immune reactions and improve host lipid metabolism were proved on DCs, mice/obese rats models and verified in limited diet clinical intervention study.

**Outlook and Expert recommendations:** 1). Direct coupling of the SHIME technology with cell culture models required for evaluation of the gut barrier and endothelial function;

2). Clinical intervention study with SGM sequencing data before and after defined diets implementation for chronic diseases treatment is necessary;

3). Comparison of SHIME integrated technology with results obtained on cells/animal experiments and *in silico* model data for evaluation of adequacy of pre-clinical and clinical tools for the following implementation of patient stratification strategy in health care system.

## A9 INTERACT-chronic care model: Self-treatment by patients with decision support e-Health solution

### Hans-Peter Brunner-La Rocca^1^, Lutz Fleischhacker^2^, Olga Golubnitschaja^3^, Frank Heemskerk^4^, Thomas Helms^5^, Tiny Jaarsma^6^, Judita Kinkorová^7^, Jan Ramaekers^8^, Peter Ruff^9^, Ivana Schnur^10^, Emilio Vanoli^11^, Jose Verdu^12^

#### ^1^Maastricht University Medical Centre, Maastricht, The Netherlands; ^2^Fleischhacker GmbH, Schwerte, Germany; ^3^The European Association for Predictive, Preventive and Personalised Medicine, Brussels, Belgium; ^4^RIMS bvba, Overijse, Belgium; ^5^German Foundation For the Chronically Ill, Fürth, Germany; ^6^Linköping University, Norrköping, Sweden; ^7^Medical Faculty Pilsen, Pilsen, Czech Republic; ^7^Sananet Care BV, Sittard, The Netherlands; ^9^Exploris AG, Zürich, Switzerland; ^10^sense.ly, San Francisco, USA; ^11^Mulimedica SPA, Milano, Italy; ^12^Medtronic Iberica SA, Madrid, Spain

##### **Correspondence:** Hans-Peter Brunner-La Rocca (hp.brunnerlarocca@mumc.nl) – Maastricht University Medical Centre, Maastricht, The Netherlands

**Keywords**: Personalised care, Prediction, Prevention, Heart failure, Co-morbidities, Telemedicine, Self-care, Caregivers, Communication, Outcome

*Patients with chronic diseases should be able to perform a substantial part of outpatient care by themselves if they are sufficiently supported by new technologies. INTERACT-chronic aims to realise a platform for chronic disease management that is evidence-based and patient-centred, gradually shifting disease-specific, caregiver driven care to integrated patient self-care.*

INTERACT-chronic stands for Integrating e-health To Efficaciously Raise the level of Contemporary Treatment in Chronic diseases. It is a pan European consortium with broad experience in e-Health solutions including university medical centres, knowledge institutes and innovative companies including a US partner. The consortium is seeking funding to reach its high ambition: the development of an e-Health system that revolutionises chronic care.

**Substantial change in delivering care urgently needed**

Health care in Western countries requires an innovative approach to provide sustainability of care and prevent the collapse of the current systems. The increasing prevalence of chronic diseases combined with their enormous economic impact and the increasing shortage of health care providers are among the most critical threats. Attempts to solve these problems have failed and future limitations in financial resources will result in much lower quality of care. Thus, changing the approach to care for chronic diseases is of utmost social importance.

**Patients in driver’s seat**

INTERACT-chronic will achieve this ambitious aim by realising a decision-support system enabling evidence-based self-management by the patients. Poor interaction and communication between stakeholders are major obstacles in delivering efficient and cost-effective care. Therefore, we will also provide a platform for an optimal interaction and communication between patients, their relatives and caregivers. This will facilitate patients’ self-management and enhance cooperation between caregivers. A comprehensive ICT system will support patients herein, focussing on care integration in complex patients. INTERACT-chronic will apply *a holistic disease management process* for preventing, predicting, diagnosing and treating chronic diseases, which *will replace traditional, disease-specific care*. INTERACT-chronic will build the ICT system upon a number of important pillars that are innovative on its own, but unique in their combination.

**Patient involvement in the whole chain**

Patient involvement in every phase of the project including implementation into clinical practice is central. This precondition is combined with the involvement of all other stakeholders who participate in the management of chronic diseases to sufficiently address the specific needs.

**User-friendly devices**

Very user-friendly front-end personal devices will enable patients, irrespective of age and education level, to adequately use the individualised e-Health system, by applying novel engagement technology to communicate with and educate the patients.

**Integrated ICT system enabling improvements**

An integrated communication and data platform for all involved stakeholders will enable fast, easy and comprehensive exchange of information and data between all involved, considering data privacy. Central storage will allow further process and use of all data. This will enable compatibility of patient data and central data processing which is a crucial prerequisite for success. The possibility to further adapt and improve the system will make the system flexible and able to enhance its quality.

**Decision support system**

A central part of INTERACT-chronic is a decision support system that compares individual patient data with available knowledge, based on guidelines and existing patient data. The decision support system will be built in a way that it is able to gradually increase the complexity of advice communicated to patients for self-management. Appropriate clinical testing will guarantee its reliability and accuracy.

**Effective support in critical decisions**

A novel, pioneering methodology to successfully evaluate the efficacy of the system will make its progress much faster than it is possible with current approaches. It also allows decisions to be made more flexible and to be handled at different levels through-out the whole chain from disease prevention to palliative care. The latter will support decisions to most efficaciously improve quality of life and to prevent patients from being exposed to expensive, but useless treatment.

**Huge social and financial impact**

The a priori approach of multiple chronic diseases reflects the need of patients much better than current care focusing on single diseases and it is a prerequisite for broad implementation of true self-management. This will result in benefits also for the society as a whole as it uses available resources much more efficiently. Therefore, the social and financial impacts will be huge. In a clinical study, we will show that our approach is superior to current standards regarding prognosis, quality of life and patient satisfaction, at much lower costs.

**New market and value network**

Thus, INTERACT-chronic aims to increase longevity of the patients while reducing total costs significantly, which as yet has been considered impossible. Once established, our approach can be extended to care in general. We aim to create a new market and value network, and eventually disrupt existing care models to facilitate and motivate patients to actively participate in both care and health. Taken together, INTERACT-chronic will address the whole chain of health and care in patients with chronic diseases. This requires not only novel and innovative technology, but also an innovative and novel vision on care and health. This combination is exactly what makes INTERACT-chronic so unique.

*Further information:*

• Hans-Peter Brunner-La Rocca, Professor of Cardiology / clinical heart failure, Maastricht University Medical Centre, NL – T +31 43 3877097, hp.brunnerlarocca@mumc.nl


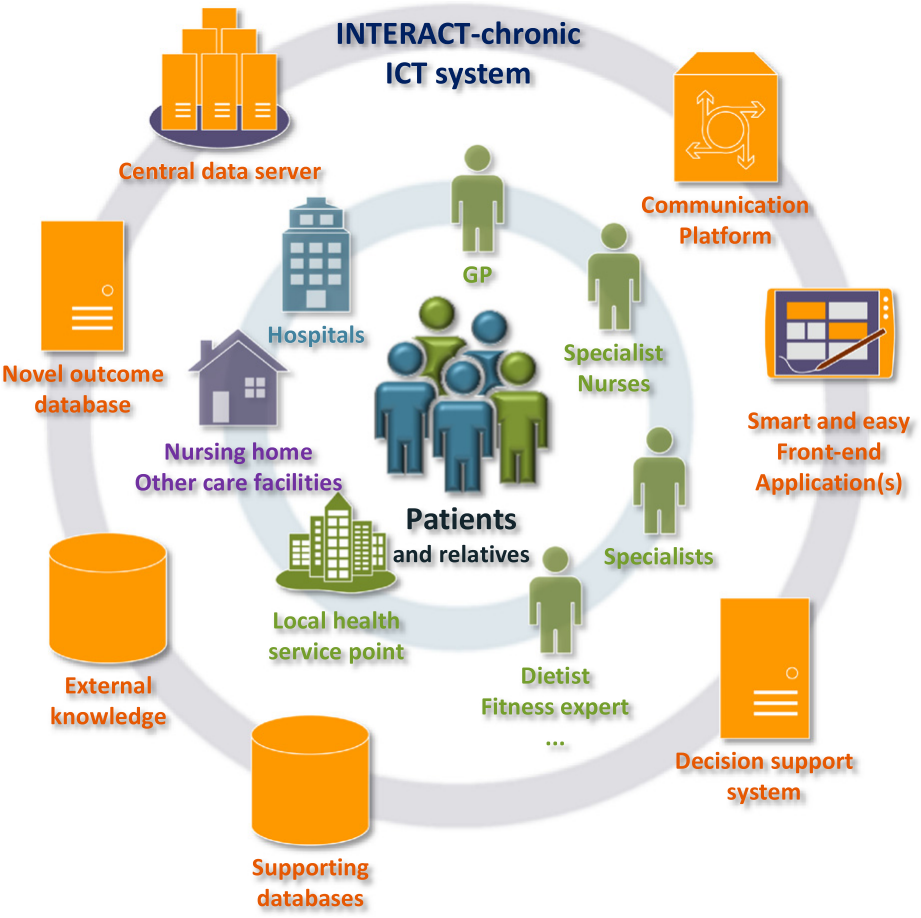


## A10 PPPM in cardiovascular medicine in 2015

### Hans-Peter Brunner-La Rocca (hp.brunnerlarocca@mumc.nl)

#### Maastricht University Medical Centre, Maastricht, The Netherlands

**Keywords**: Preventive predictive personalised medicine, Cardiovascular diseases, Cardiovascular risk factors, Exercise, Diagnosis, Treatment, Pathophysiology, Biomarkers

The big successes in cardiovascular medicine resulted in a rather unselected approach to treatment. Thus, treatment is often uniform for all patients with a particular disease, as expressed in current guidelines. Moreover, focus was and still is on treatment rather than prevention. Still, PPPM plays an important role in cardiovascular medicine that is often neglected. E.g., treatment of hypertension, hypercholestolaemia and diabetes mellitus actually is to prevent disease though often rather late.

Important steps still must be made. Prevention should start earlier and must be much more attractive since an unhealthy lifestyle obviously is currently more appealing to the majority of the population. Current campaigns are not reaching the targets and foci should be changed. E.g., interventions already during childhood are promising (e.g. integration of exercise in school), but are not implemented due to questionable reasons. Processed food which is healthy, but equally attractive as junk food may help. Recognising risk earlier with more specific interventions could have important impact for the general population.

The efforts to predict risk at every stage from health (healthy population) until advanced disease (e.g. advanced heart failure) unfortunately have not resulted in targeted interventions due to several reasons. Among others, predictive models are often retrospective and not prospectively validated. Importantly, interventions based on such models are often not prospectively tested. Thus, predicting risk does not necessarily imply that the best intervention is known to improve outcome, unless this has been specifically tested. This, however, is hardly the case.

Making this step would be important progress towards (more) personalised medicine in both preventing and treating cardiovascular diseases. Several prerequisites are important to achieve the goal of real personalised medicine. This includes detailed understanding of the pathophysiology of cardiovascular diseases. Thus, it may be not sufficient to know if a disease is present or a patient is at risk to develop a disease, but to understand the different mechanisms causatively involved in this disease. Very likely and also shown in some instances, such further classification may allow to more specifically and more efficaciously address this disease. Heart failure with preserved ejection fraction (HFpEF) is a good example for this. Moreover, likely not all patients with a certain disease profit equally from the same intervention. If not purely related to symptom reduction and several options are available, it is hardly known which intervention is most important in individual patients and which even might cause harm. If known, we could speak of real PPPM.

The question arises how we can reach this target. Among other, more specific diagnostic testing/prediction are important and biomarkers may be very helpful in this. Applying omics-approaches to find target markers from a huge set of molecules has not yet resulted in any clinically meaningful outcome, most likely because such an approach requires an enormous amount of patient data to identify targets which then have to be prospectively tested. On the short to medium term, it might be more promising to investigate specific targets that are already known or suggested to be pathophysiologically important, as the gap with clinical reality may be otherwise too large.

## A11 Magnetic resonance imaging of nanoparticles in mice, potential for theranostic and contrast media development – pilot results

### Rostyslav V. Bubnov^1,2^, Sergiy A. Grabovetskyi (purusa2@gmail.com)^1^, Olena M. Mykhalchenko (mykhalchenko.gr@gmail.com)^1^, Natalia O. Tymoshok (Timoshok@serv.imv.kiev.ua)^2^, Oleksandr B. Shcherbakov^2^, Igor P. Semeniv^1^, Mykola Y. Spivak (n.spivak@rambler.ru)^2^

#### ^1^Clinical Hospital “Pheophania” of State Affairs Department, Zabolotny Str., 21, Kyiv 03680, Ukraine; ^2^Zabolotny Institute of Microbiology and Virology, National Academy of Sciences of Ukraine, 154, Zabolotny st., Kyiv 03680, Ukraine

##### **Correspondence:** Rostyslav V. Bubnov (rostbubnov@gmail.com) – Zabolotny Institute of Microbiology and Virology, National Academy of Sciences of Ukraine, 154, Zabolotny st., Kyiv 03680, Ukraine

**Keywords:** Predictive preventive personalized medicine, Nanomedicine, Magnetic resonance imaging, Cerium dioxide nanoparticles, Gold nanoparticles, Drug delivery, Theranostic

**Introduction:** The application of nanoparticles allowing the combination of therapy and diagnosis, known as theranostic, has received increasing attention in biomedicine. Thus, nanoparticles of gold [1] and cerium dioxide [2] were reported as strong agents against oxidative damage having anti-aging activity. Nanoparticles of cerium dioxide, considering its UV-shielding effect, antiviral, antibacterial, antifungal activity, cardioprotective, neurotrophic, hepato- and nephroprotective, and anti-aging effect, have potential for various biomedical applications. Imaging is crucial for evaluation efficacy of treatment, material distribution and personalized interventions. The relevant visualization of solutions of nanoparticles on magnetic resonance imaging (MRI) provides innovative perspectives to image-guided treatment, drug delivery, development novel contrast media, etc. for ensuring theranostic approach.

The aim was to evaluate the visualization of solutions of nanoparticles with beneficial biological properties on magnetic resonance imaging.

**Methods:** We performed three consecutive experiments, each included ten mice, weight of 9 mice was 28-35 g and weight of one smaller mouse was 14-17 g; all were injected with 200 mkl of the solutions as follows:

1. saline, small mouse (14-17 g) - control;

2. saline, larger mouse (28-35 g) - control;

3. cerium dioxide nanoparticles orally;

4. cerium dioxide nanoparticles and gadolinium orally;

5. gadolinium orally;

6. cerium dioxide nanoparticles intraperitoneally;

7. cerium dioxide nanoparticles and gadolinium intraperitoneally;

8. gadolinium intraperitoneally;

9. gold nanoparticles intraperitoneally;

10. europium and cerium dioxide nanoparticles intraperitoneally.

All mice underwent magnetic resonance imaging in T1-WI VIBE iso fat suppression, TR - 18, TE - 3.8, Flip angle - 10°, fat suppression - SPAIR, resolution: FoV 190 mm (phase 100 %), slice thickness - 0.5 mm, base resolution - 256 mm, phase resolution -100 %, slice resolution - 83 %.

Signal intensity was measured at 30 and 60 min after injection in different organs and tissues three times and average value was calculated.

**Results:** We obtained heterogenous data in all experiments, the highest signals were registered in kidneys of all mice. We revealed slightly superior magnetic signal intensity in brain and muscles in mice injected with gold nanoparticles and europium with cerium dioxide nanoparticles intraperitoneally vs controls and vs mice injected with gadolinium; data are presented on Table 1.

**Conclusion:** Nanoparticles demonstrated fair contrast effect on MRI comparable with gadolinium.

**Outlook and Expert recommendations:** Preliminary results might be promising in particular to use for brain tissues imaging / interventions, considering penetrating EBB and combine application with advances of brain imaging modalities [3,4]. The follow up the study promising to focus on physical properties of nanoparticles, including sonoporation, photoacoustic imaging, ultraviolet spectra, paramagnetic properties, etc.; a study on interactions with other nanomaterials and combination with other biological (gene, regenerative) therapies.

It is recommended to create an international project to study nanoparticles application in diagnosis and therapy of neurodegenerative, heart, liver, and kidney diseases and muscle dystrophy, combining with biological therapies to achieve sustainable effects from theranostic approach.

**References**

1. Spivak MY, Bubnov RV, Yemets IM, Lazarenko LM, Tymoshok NO, Ulberg ZR. Development and testing of gold nanoparticles for drug delivery and treatment of heart failure: a theranostic potential for PPP cardiology. EPMA J. 2013;4(1):20.

2. Zholobak NM, Sherbakov AB, Babenko LS, Bogorad-Kobelska OS, Bubnov RV, Spivak MY et al. The perspectives of biomedical application of the nanoceria. EPMA J. 2014;5(Suppl 1):A136.

3. Grabovetskyi S. Proton Magnetic Resonance Spectroscopy of Focal Intracranial Lesions: Role in Clinical Practice. J Cancer Prev Curr Res. 2015;2(5):00052. doi: 10.15406/jcpcr.2015.05.00052

4. Sinnecker T, Kuchling J, Dusek P, Dörr J, Niendorf T, Paul F, et al. Ultrahigh field MRI in clinical neuroimmunology: a potential contribution to improved diagnostics and personalised disease management. EPMA J. 2015;6:16.

**Table 1 (abstract A11).** Avarage signal intensity after injection in organs and tissues

Tissue/organ	Specimen*									
	1	2	3	4	5	6	7	8	9	10
muscle	520	577	560	557	590	598	555	500	552	670
liver	n/a	537	512	554	626	514	590	541	545	590
kidneys	n/a	706	630	666	642	622	590	627	n/a	646
brain	530	n/a	510	496	484	566	558	557	556	530
heart	n/a	504	512	577	549	444	566	481	507	460

* number corresponds to the injected substance, as in Methods paragraph

## A12 Ultrasound diagnosis for diabetic neuropathy - comparative study

### Rostyslav V. Bubnov^1,2^, Tetyana V. Ostapenko (ter2@ukr.net)^3^

#### ^1^Centre of Ultrasound Clinical Hospital “Pheophania” of State Affairs Department, Zabolotny Str., 21, Kyiv 03680, Ukraine; ^2^Zabolotny Institute of Microbiology and Virology, National Academy of Sciences of Ukraine, 154, Zabolotny st., Kyiv 03680, Ukraine; ^3^Endocrinology centre, Clinical Hospital “Pheophania” of State Affairs Department, Zabolotny Str., 21, Kyiv 03680, Ukraine

##### **Correspondence:** Rostyslav V Bubnov (rostbubnov@gmail.com) – Zabolotny Institute of Microbiology and Virology, National Academy of Sciences of Ukraine, 154, Zabolotny st., Kyiv 03680, Ukraine

**Keywords:** Predictive preventive personalized medicine, Diabetes mellitus type 1 and 2, Ultrasonography, Peripheral nerve, Neuropathy

**Introduction:** The diagnostic ultrasound (US) is used for peripheral nerves visualization, particularly in regional anesthesia; recently we formulated diagnostic markers of peripheral neuropathy, based on ultrasound of nerve structure changes [1]. The US characteristics for peripheral neuropathy diagnosis in patients with diabetes mellitus (DM), in particular by distinguishing types 1 and 2 are not studied yet. PainDETECT is an effective screening questionnaire to identify neuropathic components in patients with back pain [2].

**The aim** of paper was to study the nerve structure changes in patients with diabetes mellitus type 1 and 2.

**Materials and Methods:** We included 30 patients of both genders, assessed into the following two groups: 1 (n = 15) - DM type 1 (16-32 years old); group 2 (n = 15) - DM type 2 (48-63 years old) with period of disease 1-5 years, suffering from clinically diagnosed neuropathy of lower limbs. Deference in age bias was unavoidable due to difference in age of first manifestation and development of DM I and DM II, but period of disease was similar. As a control group we included 20 volunteers (18-54 years) without clinical disturbance of peripheral nerves.

We conducted survey using linear 12 MHz ultrasound probe performing scanning at the lower femoral level from posterior access. We measured fascicular diameter, nerve cross-section area [1]. Patients underwent a standardized nerve motor and sensitive nerve conduction study and responded PainDetect questionnaire (1-38) scores [2].

**Results:** In patients of the first group the ultrasound symptoms of nerve structure disorder were revealed: the changes of nerve cross-sectional area were insignificant among groups (P >0.05); swelling of the nerve bundles; fascicular diameter was 1.8 mm in first group (p < 0.01), and 1.1 mm in second and 0.9 in control group (in 83 % patients). Indirect US signs of neuropathy are registered as follows: reducing number of nerve bundles, increase in the fibrous component, inequality nerve path, thinning of the muscles and reducing the amplitude of movements.

**Conclusion:** Ultrasonography is an effective diagnostic tool for diabetic neuropathy, in particular for early diagnosis in patients with DM type 1 in young age.

**Outlook and Expert recommendations:** According to obtained results, ultrasonography has strong diagnostic predictive potential for neuropathy, much expressed in patients with DM type 1. Further studies for correlation imaging--neurophysiological molecular data and validation are needed.

Current diagnostic imaging information should impact on the diagnostic thinking of the clinician, effect on the patient management plan, improving the therapeutic outcome and have essential societal benefits of implementing this diagnostic imaging technology. It is recommended to initiate screening program for diabetic patients with DM type 1 for early diagnosis, patient stratification and preventive measures in young age, and to organize relevant educational programs.

**References**

1. Bubnov RV. Ultrasonography diagnosis of peripheral neuropathy. The initial experience. Ultrasound Med Biol. 2011;37(Suppl 1):S144-5.

2. Freynhagen R, Baron R, Gockel U, Tölle TR. PainDETECT: a new screening questionnaire to identify neuropathic components in patients with back pain. Curr Med Res Opin. 2006;22(10):1911-20.

## A13 Ultrasound for stratification patients with diabetic foot ulcers for prevention and personalized treatment - pilot results

### Rostyslav V. Bubnov^1,2^, Nazarii M. Kobyliak (nazariikobyliak@gmail.com)^3^, Nadiya M. Zholobak (n.zholobak@gmail.com)^1^, Mykola Ya. Spivak (n.spivak@rambler.ru)^1^

#### ^1^Zabolotny Institute of Microbiology and Virology, National Academy of Sciences of Ukraine, Zabolotny Str., 154, Kyiv 03680, Ukraine; ^2^Clinical Hospital ‘Pheophania’ of State Management of Affairs Department, Zabolotny Str., 21, Kyiv 03680, Ukraine; ^3^Bogomolets National Medical University, T. Shevchenko boulevard, 13, Kyiv 01601, Ukraine

##### **Correspondence:** Rostyslav V. Bubnov (rostbubnov@gmail.com) – Zabolotny Institute of Microbiology and Virology, National Academy of Sciences of Ukraine, Zabolotny Str., 154, Kyiv 03680, Ukraine

**Keywords:** Predictive preventive personalized medicine, Diabetic foot ulcer, Ischemia, Congestion, Neuropathy, Ultrasound, Cerium dioxide nanoparticles, Nanomedicine

**Introduction:** The “diabetic foot” is a group of syndromes in which neuropathy, ischemia, and infection lead to tissue breakdown. Approximately 60 % of all diabetic foot ulcers (DFU) result from neuropathy; of these, half are related to peripheral arterial disease [1]. Complications of DFUs, such as infections and gangrene, frequently lead to hospitalization and to extensive tissue destruction or amputation and impaired quality of life, incur a significant cost economically, socially and psychologically [2,3]. Even when ulcers are healed, >50 % will have a recurrence after 3 years [2]. Neuropathic ulcers, deep foot infection, site of ulcer (plantar for foot ulcer, metatarsal head) and co-morbidity (non-retinal eye disease, end-stage renal disease, edema, walking disability) are related to minor or major amputation in non-ischemic patients. Edema is usually of multifactorial origin, is related to outcome both in neuropathic and neuro-ischemic ulcers, and should be treated accordingly. Diabetes with neuropathy and a long-standing plantar ulcer increases the risk of infection and an infected foot ulcer precedes about two thirds of amputations [4]. Stratification of DFU according neuropathic; ischemic and congestive mechanism is crucial for prevention of ulceration and personalized treatment [5].

**The Aim** was to evaluate ultrasound signs for stratification DFU according neuropathic; ischemic and congestive mechanism and personalized preliminary evaluate treatment efficacy of nanoceria.

**Materials:** We included 14 patients (9 males, 5 females, age was 52-73 years) with diabetic foot ulcers (DFU). Ultrasound survey was conducted using 12-18 MHz probes of GE Logic equipment. There were no signs of osteomyelitis on Ro. All were treated according to the standards including insulin therapy, gauze with hypertonic solution (0.45 % NaCl) in the wound channel; antibiotic therapy (lincomycin intramuscular on course for 7 days); daily dressings.

**Results:** We revealed dominating of several ultrasound patterns as follows:

In *neuropathic pattern* (n = 9) ultrasound finding demonstrated signs of diabetic neuropathy of lower limb nerves– we defined thickening fascicles of nerves to 2-4 mm (while normally to 1-2 mm) with pronounced inter-fascicular fibrosis with normal arterial and venous blood flow. During all cases in the phase of wound healing we observed granulations with intensive local vascularization - positive predictive sign. In patients with gout we found uric acids deposits inside the joints and in granulation tissue in scars that slowed down the healing. A small amount of liquid content might be considered as wound exudates or an inflammation.

In *ischemic pattern* (n = 6) the typical presentation of reduced blood flow and multiple arteries occlusions and strongly declines peripheral circulation, no swelling and nerve changes were revealed.

In *congestive pattern* (n = 3) we revealed extensive vein congestion, soft tissues edema and joints effusions, accompanied with lymphadenopathy, lymphostasis, and deep veins thrombosis. Arterial blood flow and nerves structure remained relatively unchanged. We revealed bone surface deformations with small ulcerations.

*Personalized treatment approaches*

Recently Chigurupati et al. reported that topical application of water soluble cerium oxide nanoparticles (*Nanoceria*) accelerates the healing of full-thickness dermal wounds in mice by a mechanism that involves enhancement of the proliferation and migration of fibroblasts, keratinocytes and VECs. The nanoceria can penetrate into the wound tissue and reduced oxidative damage to cellular membranes and proteins, suggesting a therapeutic potential for topical treatment of wounds with antioxidant nanoparticles [7].

In three patients after ineffective standard treatment we performed dermotropic ointment based on *cerium dioxide nanoparticles* in the wound channel 2 times daily (in the morning and evening time).

After treatment we observed fast decreasing of ulcer total healing in all patients. On ultrasound we observed intensive vascularization, tissue regeneration signs.

After successful treatment of previous DFU two patients were re-hospitalized on with recidivism and underwent the same treatment strategy with positive outcome.

**Conclusions:** Ultrasound is effective for stratification DFU according neuropathic; ischemic and congestive mechanism. Local application of cerium dioxide nanoparticles weas efrfective for DFU wound healing.

**Outlook and Expert recommendations:** Since preliminary results provide good perspectives for use of nanoceria for DFU treatment we suggest to create an international research project to study the biomedical effects of nanoceria offer the prospect of its use as a UV protectant, a drug with antiviral, antibacterial and antifungal activity, as well as means capable of reducing the level of oxidative stress in diverse tissues of human body. The larger studies with long term assessment are required. Study of effects of nanoceria on *vasospasm* is necessary for stratifications of potential responders to formulate clear personalized application.

Considering biosafety of ceria nanoparticles, the group of potential patients (consumers) of person-related smart physiologic low-dose treatments can be large.

**References**

1. Campbell I. Diabetic foot disease. Br J Diabetes Vasc Dis. 2011;11:53–4.

2. Boulton AJM, Vileikyte L, Ragnarson-Tennvall G, Apelqvist J. The global burden of diabetic foot disease. Lancet. 2005;366:1719–24.

3. Steed DL, Attinger C, Colaizzi T, Crossland M, Franz M, Harkless L, et al. Guidelines for the treatment of diabetic foot ulcers. Wound Repair Regen. 2006;14:680–92.

4. Gershater MA, Löndahl M, Nyberg P, Larsson J, Thörne J, Eneroth M, et al. Complexity of factors related to outcome of neuropathic and neuroischaemic/ischaemic diabetic foot ulcers: a cohort study. Diabetologia. 2009;52(3):398-407.

5. Huang Y, Xie T, Cao Y, Wu M, Yu L, Lu S, et al. Comparison of two classification systems in predicting the outcome of diabetic foot ulcers: the Wagner grade and the saint elian wound score systems. Wound Repair Regen. 2015;23(3):379-85.

6. Chigurupati S, Mughal MR, Okun E, Das S, Kumar A, McCaffery M, et al. Effects of cerium oxide nanoparticles on the growth of keratinocytes, fibroblasts and vascular endothelial cells in cutaneous wound healing. Biomaterials. 2013;34(9):2194-201.

7. Chigurupati S, Mughal MR, Okun E, Das S, Kumar A, McCaffery M, et al. Effects of cerium oxide nanoparticles on the growth of keratinocytes, fibroblasts and vascular endothelial cells in cutaneous wound healing. Biomaterials. 2013;34(9):2194-201.

## A14 Project ImaGenX – designing and executing a questionnaire on environment and lifestyle risk of breast cancer

### John Paul Cauchi (john.p.cauchi@um.edu.mt)

#### Department of Physiology and Biochemistry, University of Malta, Msida, Malta

**Keywords:** Breast cancer, Environmental health, Lifestyle, Cancer

Project ImaGenX is an Italia-Malta EU project between six partners which seeks to strengthen breast cancer surveillance in both countries, while also identifying genetic and environmental or lifestyle risks that could affect breast cancer. The environmental and lifestyle risks questionnaire was designed by the University of Malta together with Palermo University, and has been carried out in Malta, Palermo and Siracusa in an attempt to see which factors might influence the risk of developing cancer. This retrospective case-control study focuses on risks at the workplace (exposure), diet and lifestyle, based on available literature on the subject, and is mostly exploratory in nature.

In Malta, over 600 participants were randomly selected and contacted (200 cases and 400 controls), with cases being women with breast cancer without a family history – so-called “sporadic” cases and controls being women who have not had breast cancer. Interviews were carried out face-to-face, and participation was entirely voluntary. This presentation will focus on the methodology and challenges of this study, its limitations, its possible data outcomes and possible ramifications of this study within local public health scenario.

## A15 Genomics – a new structural brand of predictive, preventive and personalized medicine or the new driver as well?

### Dmitrii Cherepakhin^1^, Marina Bakay^2^, Artem Borovikov^1^, Sergey Suchkov^1^

#### ^1^First Moscow State Medical University, Moscow, Russia; ^2^The Children's Hospital of Philadelphia, Philadelphia, USA

##### **Correspondence:** Dmitrii Cherepakhin (dimadimacher@gmail.com) – First Moscow State Medical University, Moscow, Russia

**Keywords:** Genomics, Personalized medicine, EPMA, Genes, Genomics, Metabolomics, Microbiology

Genomics is one of the most important unit of predictive, preventive and personalized medicine (PPPM). All diseases of human body start in molecular level and one of the main pathological pathways include genes destruction. All diseases can manifest in any time of human life or hide in another disease. In our time, we have a big permissions to diagnose this molecular and genes destruction. Nowadays in scientific article we can noticed a huge splash of attention to genetic tests and way of diagnosis genetic diseases. Day by day, we come to the most accurate and the most informational result of genetic test, but we have some problems with it. The first - its money and the second - we do not have enough qualified employees. We can solve problem with qualified employees. In last EPMA congress, our group developed a project about special medical education. The creations and developments of the special elective course at school helps students to get acquainted with broad spectrum directions of PPPM and they must have a clinical practice or take part in research in the structure of PPPM. Students must take the knowledge about PPPM, when they study at school. Now we have the first results of our work. In this day, we have special scientific group, which include students at school, medical students in university and medical professors in university. This system of scientific work helps student at school take knowledge from medical student, but also can help for them take a part in scientific work. As a result, the work we can say that next year our group will release Medical English-Russian dictionary. It will be the biggest and the fullest dictionary about medicine. It will consist of new spheres of medicine such as PPPM, genomics, metabolomics, microbiology and so one. And the main idea that in work on this dictionary were included our school students and they did a big part of this dictionary. We can give the name of our system, for example, “From young professional to mentor”.

## A16 Survey of questionnaires for evaluation of the quality of life in various medical fields

### Barbara Cieślik^1^, Agnieszka Migasiewicz^1^, Maria-Luiza Podbielska^2^, Markus Pelleter^3^, Agnieszka Giemza^3^, Halina Podbielska^4,1^

#### ^1^Wroclaw University School of Physical Education, Faculty of Physiotherapy, Wroclaw, Poland; ^2^Inwestasekur, Wroclaw, Poland; ^3^VIANESSE AG, Research and Development, Engelberg, Switzerland; ^4^Wroclaw University of Technology, Department of Biomedical Engineering, Wroclaw, Poland

##### **Correspondence:** Halina Podbielska (info@halinapodbielska.pl) – Wroclaw University of Technology, Department of Biomedical Engineering, Wroclaw, Poland

**Keywords:** Quality of life, Measurement methods, Questionnaires

**Scientific objectives:** Numerous questionnaires are used e.g. for study therapeutic efficiency in miscellaneous medical fields. Recently, the quality of life, not only the therapeutic responses, is in focus of many studies. It is recognized that the quality of life is dependent on specific parameters: the person's physical health, psychological state, level of independence, social relationships, and their relationship to salient features of their environment. Various questionnaires are used for this purpose. The objective of this study is to statistically evaluate the questionnaires adopted in various medical fields to measure the quality of life.

**Technological approaches:** The published papers, majority of them recorded in PubMed, were the primary sources of the information. The following fields were taken into account: cardiology, rheumatology, neurology, gastrology, transplantology, diabetes, oncology, gynecology, dermatology, dietetics and sport science.

**Results interpretation:** The most frequently used questionnaires are: The Medical Outcomes Study 36-Items Short – Form Health Survey (SF-36), The Short Form 12 (SF-12), The Six-Dimensional Health State Classification Short Form 6D (SF-6D), Euro-Quality of Life Questionnaire (EQ-5D), and The World Health Organization Quality of Life – BREF (WHOQOL-BREF).

Our analysis shows that SF-36 was employed most frequently and ranged between 60 % and 100 % in all of the 130 papers that were evaluated. At 20–50 %, the second most frequently employed questionnaire was EQ-5D. Overall, scientific development shows that there is an increasing number of studies on the quality of life. As and example until 1995, 512 questionnaire studies on the quality of life were recorded in the PubMed database, were us until 2014, this figure rose to 6325.

**Outlook and Expert recommendations:** As a result of the growing interest on the quality of life and the quite inexpensive data collection that requires little effort, a further rise in the number of studies on the quality of life using questionnaires can be expected. Contemporary medical science is concentrated not only on diagnosis and treatment and the improving the general quality of life is important, as well. Predictive, preventive and personalized approach should consider the life quality assessment.

## A17 Personalized molecular treatment for muscular dystrophies

### Sebahattin Cirak (Sebahattin.Cirakuk-koeln.de)^1,2,3^

#### ^1^Center for Molecular Medicine, University Hospital Cologne, Cologne, Germany; ^2^Institut für Humangenetik, Universitätsklinikum Köln, Köln, Germany; ^3^Klinik und Poliklinik für Kinder- und Jugendmedizin, Universitätsklinikum Köln, Köln, Germany

**Keywords:** Muscular dystrophy, Rare diseases, Drug treatment, Exon skipping, Predictive preventive personalized medicine

Muscular dystrophies (MD) are representing a clinical and genetically heterogeneous group of severe progressive disorders often fatally leading to death. Despite recent advances, unfortunately in about 50 % of the patients disease genes are unknown. Currently, there are no curative treatments available for muscular dystrophies. There is a disease ameliorating therapy in development for the common form Duchenne Muscular Dystrophy based on Exon skipping therapy [1, 2]. A major obstacle for translational research is unfortunately that for many MD forms even the underlying disease genes and mechanisms are unknown. There is an unmet medical need for an integrated systems approach to understand the genetic, biochemical and pathophysiological basis of MD for the development of personalized medicine.

By whole exome sequencing, we have discovered 3 new genes (*ISPD, B3GALNT2 and GMPPB*). The biochemical function of *isoprenoid synthase domain containing (ISPD)* in mammals remain unknown. Remarkably, we identified a novel CMD phenotype harbouring mutations in *ISPD* characterized by LGMD, oculomotor apraxia, myopia and cerebellar hypoplasia. Further Genotype-Phenotype correlations of muscular dystrophies are investigated using combined next generation sequencing approaches from >200 cases will be shown.

To explore the possible effects of missense mutations ISPD, we have mapped them onto the homology model of human ISPD derived from the structure of a related bacterial protein (CDP-ME synthase; 1VPA). We have expressed recombinant His-tagged wt and mut. ISPD protein in E.coli and purified Co-IMAC affinity chromatography. By the use of Thermofluor we could confirm the differences in the stability between wt and mut. ISPD proteins. We using Thermofluor for screening of chemical chaperones in order to develop personalized drugs.

To enable clinical trials, we performed a pilot study for the discovery of serum biomarkers in LGMD2I and could identify various candidates, which we grouped into a) myofibrillar proteins b) glycolytic enzymes, c) extracellular matrix and d) other muscle specific proteins.

**References**

1. Lu QL, Cirak S, Partridge T. What Can We Learn From Clinical Trials of Exon Skipping for DMD? Mol Ther Nucleic Acids. 2014;3:e152.

2. Cirak S, Arechavala-Gomeza V, Guglieri M, Feng L, Torelli S, Anthony K, et al. Exon skipping and dystrophin restoration in patients with Duchenne muscular dystrophy after systemic phosphorodiamidate morpholino oligomer treatment: an open-label, phase 2, dose-escalation study. Lancet. 2011;378(9791):595-605.

## A18 Secondary mutations in circulating tumour DNA for acquired drug resistance in patients with advanced ALK+NSCLC

### Marzia Del Re^1^, Paola Bordi (paolabordi@yahoo.it)^2^, Valentina Citi (valentina.citi@for.unipi.it)^1^, Marta Palombi (martapalo@yahoo.it)^1^, Carmine Pinto (cpinto@ao.pr.it)^2^, Marcello Tiseo (mtiseo@ao.pr.it)^2^, Romano Danesi (romano.danesi@unipi.it)^1^

#### ^1^Clinical Pharmacology and Pharmacogenetics Unit, Department of Clinical and Experimental Medicine, University of Pisa, Pisa, Italy; ^2^Medical Oncology Unit, University Hospital of Parma, Parma, Italy

##### **Correspondence:** Marzia Del Re (marzia.delre@for.unipi.it) – Clinical Pharmacology and Pharmacogenetics Unit, Department of Clinical and Experimental Medicine, University of Pisa, Pisa, Italy

**Keywords:** Circulating tumor DNA, NSCLC, ALK, Acquired resistance, TKI

**Scientific objectives:** ALK translocation is present in about 5 % of advanced NSCLC and is a predictive factor of response to ALK Tyrosine Kinase Inhibitors (TKI), such as crizotinib [1]. Unfortunately, disease progression occurs after a median period of 9-10 months of treatment with crizotinib. Several mechanisms of resistance have been identified and include other mutations in ALK gene, ALK amplification, activation of bypassing signaling pathways involving EGFR, KRAS and c-KIT [2]. Second-generation ALK-TKIs demonstrated an enhanced spectrum of activity in crizotinib-resistant ALK mutants. However, re-biopsy in NSCLC patients represents a critical issue and analysis of circulating cell-free DNA (cftDNA) has a promising role for identification of mechanisms of resistance to targeted therapy.

**Technological approaches:** Twelve crizotinib treated patients were enrolled. After tumor progression, blood was collected and plasma isolated by centrifugation. DNA was extracted from plasma using QIAamp circulating nucleic acid kit (Qiagen®) and tested for ALK secondary mutations, KRAS exon 12 mutations and BRAF V600E, using a Digital Droplet PCR (BioRad®).

**Results interpretation:** Eleven patients received crizotinib and only 1 ceritinib. ALK-TKIs was administered mainly as second-line, in 2 cases as first-line and in the remaining as third-line therapy. Best response was partial in 10 patients and stable disease in 2. Median PFS was 16.9 months. ALK secondary point mutations were identified in 3 patients, all female, never-smokers and treated with crizotinib. The first showed p.L1196M and p.G1269A ALK mutations; their plasma levels decreased after the 2 months of therapy with second generation ALK-TKI, in association to tumor response. The second presented p.L1196M while the third, initially wild-type, showed p.F1174L after initiation of second generation ALK-TKI. In a total of 9 patients, including those with secondary ALK point mutations, the KRAS mutation G12D or G12V appeared in blood samples at the time of resistance to TKI.

**Outlook and Expert recommendations:** ddPCR can detect resistance mutations in cftDNA of ALK+ NSCLC and may represent an effective alternative to re-biopsy. Moreover, the assessment of mutated allele burden could be used for response monitoring during treatment. Moreover, the development of KRAS mutations may play a role in resistance to ALK-TKIs.

**References**

1. Alkan A, Köksoy EB, Utkan G. First-line crizotinib in ALK-positive lung cancer. N Engl J Med. 2015;372(8):781-2.

2. Toyokawa G, Seto T. Updated Evidence on the Mechanisms of Resistance to ALK Inhibitors and Strategies to Overcome Such Resistance: Clinical and Preclinical Data. Oncol Res Treat. 2015;38(6):291-8.

## A19 Recombinant species-specific FcεRI alpha proteins for diagnosis of IgE-mediated allergies in dogs, cats and horses

### Lukas Einhorn^1,2^, Judit Fazekas (judit.fazekas@meduniwien.ac.at)^1,2^, Martina Muhr (0808663@students.vetmeduni.ac.at)^1,2^, Alexandra Schoos (Alexandra.Schoos@vetmeduni.ac.at)^1,2^, Lucia Panakova (lucia.panakova@vetmeduni.ac.at)^3^, Ina Herrmann (ina.herrmann@vetmeduni.ac.at)^3^, Krisztina Manzano-Szalai (krisztina.szalai@meduniwien.ac.at)^1^, Kumiko Oida (kumiko.oida@vetmeduni.ac.at)^1,4^, Edda Fiebiger (Edda.Fiebiger@childrens.harvard.edu)^5^, Josef Singer (josef.singer@meduniwien.ac.at)^2^, Erika Jensen-Jarolim (Erika.jensen-jarolim@meduniwien.ac.at)^1,2^

#### ^1^Comparative Medicine, Messerli Research Institute of the University of Veterinary Medicine Vienna, Medical University Vienna and University Vienna, Vienna, Austria; ^2^Comparative Immunology and Oncology, Institute for Pathophysiology and Allergy Research, Center of Pathophysiology, Infectiology and Immunology, Medical University of Vienna, Vienna, Austria; ^3^Department for Companion Animals and Horses, University of Veterinary Medicine Vienna, Vienna, Austria; ^4^Laboratory of Veterinary Molecular Pathology and Therapeutics, Tokyo University of Agriculture and Technology, Tokyo, Japan; ^5^Division of Gastroenterology and Nutrition, Boston Children's Hospital and Department of Pediatrics, Harvard Medical School, Boston, USA

##### **Correspondence:** Lukas Einhorn (lukas.einhorn@meduniwien.ac.at) – Comparative Medicine, Messerli Research Institute of the University of Veterinary Medicine Vienna, Medical University Vienna and University Vienna, Vienna, Austria

**Keywords:** FcεRIα, IgE, IgE Fc receptor, Dog, Cat, Horse, Allergy diagnosis

**Background:** Domestic animals, such as dogs, cats and horses develop IgE-mediated allergies comparably to humans (1). Common symptoms include chronic pruritus, papules, erythema, sometimes urticarial lesions and, in cats and horses, asthma. The most prominent clinical phenotype is canine, feline or equine atopic dermatitis (AD) elicited by respiratory (2), food or insect venom allergens. In humans, component resolved diagnosis of allergy using antibodies for IgE detection has recently entered clinical routine. In cats, dogs and horses allergy diagnosis is so far performed with allergen extracts, but recently the usage of the human alpha chain of the high affinity IgE receptor (FcεRIα) is implemented for IgE detection in veterinary diagnosis (3, 4). The high interspecies amino acid homology (54-56 %) is responsible for binding of human FcεRIα with IgE of these species. We hypothesized, however, that the IgE detection could be improved by the use of recombinant species-specific FcεRIα in allergic dogs, cats and horses.

**Methods:** Canine, feline and equine recombinant FcεRIα (rFcεRIα) were expressed in CHO- DUKX B11 cells using a custom SV40_Neo mammalian expression vector. 384 clones of each species were evaluated for their production of IgE-binding rFcεRIα by immunoblotting and Enzyme-Linked Immunosorbent Assay (ELISA) prior to isolation of rFcεRIα from selected clones via anti-FLAG M2 affinity purification.

**Results:** Proper structure of rFcεRIα proteins was confirmed by CD-spectroscopy. Immunoblot and ELISA experiments verified that the three rFcεRIα proteins were able to bind IgE for the respective species with high affinity to serum IgE of each species. Next, the IgE binding capacities of the recombinant alpha chains will be compared in solid phase assays.

**Conclusion:** The detection of serum IgE of veterinary patients by the application of the three species-specific rFcεRIα may offer further tools for improved allergy diagnosis in allergic veterinary patients in the future.

**Sources of funding:** The study was supported by the Austrian Science Fund (FWF) grants SFB F4606-B19, W 1248-B13 (MCCA), and in part by P23398-B11 and W1205-B09 (CCHD).

**References**

1. Schäfer T, Merkl J, Klemm E, Wichmann HE, Ring J. We and our pets: allergic together? Acta Vet Hung. 2008;56(2):153-61.

2. Jensen-Jarolim E, Einhorn L, Herrmann I, Thalhammer JG, Panakova L. Pollen Allergies in Humans and Dogs, Cats and Horses: differences and similarities. Clin Transl Allergy. 2015;5:15.

3. DeBoer DJ, Hillier A. The ACVD task force on canine atopic dermatitis (XV): fundamental concepts in clinical diagnosis. Vet Immunol Immunopathol. 2001;81(3-4):271-6.

4. Stedman K, Lee K, Hunter S, Rivoire B, McCall C, Wassom D. Measurement of canine IgE using the alpha chain of the human high affinity IgE receptor. Vet Immunol Immunopathol. 2001;78(3-4):349-55.

## A20 Global methodology for developmental neurotoxicity testing in humans and animals early and chronically exposed to chemical contaminants

### Arpiné A. Elnar^1^, Nadia Ouamara^2^, Nadiya Boyko^3^, Xavier Coumoul^4^, Jean-Philippe Antignac^5^, Bruno Le Bizec^5^, Gauthier Eppe^6^, Jenny Renaut^7^, Torsten Bonn^7^, Cédric Guignard^7^, Margherita Ferrante^8^, Maria Liusa Chiusano^9^, Salvatore Cuzzocrea^10^, Gerard O'Keeffe^11^, John Cryan^11^, Michelle Bisson^12^, Amina Barakat^13^, Ihsane Hmamouchi^13^, Nasser Zawia^14^, Anumantha Kanthasamy^15^, Glen E. Kisby^16^, Rui Alves^17^, Oscar Villacañas Pérez^18^, Kim Burgard^19^, Peter Spencer^20^, Norbert Bomba^21^, Martin Haranta^21^, Nina Zaitseva^22^, Irina May^22^, Stéphanie Grojean^23^, Mathilde Body-Malapel^24^, Florencia Harari^25^, Raul Harari^25^, Kristina Yeghiazaryan^26^, Olga Golubnitschaja^26^, Vittorio Calabrese^8^, Christophe Nemos^1^, Rachid Soulimani^1^

#### ^1^Université de Lorraine, Metz, France; ^2^CHR Metz-Thionville, Pôle mère-enfant, Metz, France; ^3^Cassovia Life Sciences, Research and Innovation, Košice, Slovakia; ^4^Université Paris Descartes, Paris, France; ^5^Ecole Nationale Vétérinaire, Agroalimentaire et de l’Alimentation, Nantes, France; ^6^Université de Liège, Liège, Belgium; ^7^Luxembourg Institute of Sciences and Technology, Belvaux, Luxembourg; ^8^University of Catania, Catania, Italy; ^9^University Federico II of Naples, Naples, Italy; ^10^University of Messina, Messina, Italy; ^11^University College Cork, Cork, Ireland; ^12^Institut National de l'Environnement Industriel et des Risques, Paris, France; ^13^University Hospital, Rabat, Morocco; ^14^University of Rhodes Island, Rhodes Island, USA; ^15^Iowa State University, Ames, IA, USA; ^16^Western University of Health Sciences, Oregon, USA; ^17^Biomedical Research Institute of Lleida, Lleida, Catalunya, Spain; ^18^CEO - Owner at Intelligent Pharma, Barcelona, Spain; ^19^Laboratoires Réunis, Luxembourg, Luxembourg; ^20^Oregon Health & Science University, Oregon, USA; ^21^PAMIDA International, Kysucké Nové Mesto, Slovakia; ^22^University of Perm, Perm, Russia; ^23^European Drug Development Hub, Nancy, France; ^24^Université de Lille II Droit et Santé, Lille, France; ^25^Institute for Production and Work Environment Development, Quito, Ecuador; ^26^European Association for Predictive and Personalised Medicine, Brussels, Belgium

##### **Correspondence:** Arpiné A. Elnar (arpine.el-nar@univ-lorraine.fr) – Université de Lorraine, Metz, France

**Keywords:** OMICs approaches, Predictive markers, Chemical contaminants, Central nervous system

The nervous system of children is especially vulnerable to chemical exposure because of a long developmental period beginning shortly after conception and continuing through adolescence. The complex developmental process requires precise coordination of cell growth, migration and network formation. Brain development can be disrupted by even short-term exposures to chemical agents during critical periods of maturation (i.e., fetal, neonatal and adolescence). This disruption can lead to permanent functional deficits and may predispose to disease later in life. Epidemiological studies suggest an increasing incidence of mental and neurological disorders among children and adults, including autism, attention-deficit hyperactivity disorder and neurodegenerative diseases such as Alzheimer disease. Environmental chemical mixtures, especially when exposures occur early in life, are suspected to contribute to the etiologies of these disorders. Developmental neurotoxicants are among the top 50 compounds listed by ATSDR they include: heavy metals (lead, methylmercury, cadmium…), polychlorinated biphenyls (PCBs), polycyclic aromatic hydrocarbons (PAHs), polybrominated diphenyl ethers (PBDEs) and organochlorine pesticides, namely dichlorodiphenyltrichloroethane (DDT) and hexachlorocyclohexane (HCH). According to the European Commission, there are 143,000 registered chemicals, many which have not been evaluated for possible neurotoxic properties, let alone their effect on the developing human brain.

In this conference, a global methodology using *in vivo*, *ex vivo* and *in vitro* tests as predictive markers for the screening of neurotoxic potential of chemical contaminants will be presented.

## A21 Mental indicators at young people with attributes hypertension and pre-hypertension

### Maria E. Evsevyeva, Elena A. Mishenko, Zurida V. Kumukova, Evgeniy V. Chudnovsky, Tatyana A. Smirnova

#### Stavropol State Medical University, Stavropol, Russia

##### **Correspondence:** Maria E. Evsevyeva (evsevieva@mail.ru) – Stavropol State Medical University, Stavropol, Russia

**Keywords:** Young people, Arterial pressure, Mental indicators

**The actuality:** It was found that the mechanisms of pressure increase are largely dependent on age [1, 2]. In particular, it is assumed that in the development of arterial hypertension (AH) in young contingent significant role is played by the various psychogenic factors [3, 4]. However, such studies carried out are not enough.

**Objective:** Identification of personality profiles for young people including self-centeredness, stress stability, anxiety-depressive disorders (ADD) and features of stress reactivity.

**Material and Methods:** 147 young men aged from 18 till 25 years were examined. Three groups were created: 1 – normal blood pressure (BP) (n = 81); 2 – high normal BP, i.e. prehypertension (PH) (n = 30); 3 – AH (n = 36). For assessment of psychological status the hospital scale of alarm and depression (HADS), questionnaire of stress-resistance and drawing test were used. Last test revealed the various personal qualities of the subject, including creativity, self-esteem, self-centeredness and others. At height of test "The Mathematical Scoring" and in restoration assessment of haemodynamic signs were carried out. Statistical processing was carried out by means of the program BioStat.

**Results:** The tendency of reduction of stress-resistance in groups with increased BP is revealed. The lowered stress-resistance was observed for a third of young men with PH, practically a half of ones with AH and only 16 % of persons with normal BP. A third of young men with PH and a half of young people with AH alarm signs came to light. ADD subclinical is revealed in 12 % and 5 % of persons from the second and third groups of observation. The most significant reaction BP and rhythm in response to stress-test is noted at persons with signs of AH and PH. In individuals from groups of increase BP were significantly more marked with presence of high self-esteem and the desire to achieve high scores of people around.

**Conclusion:** Young people with existence not only AH, but also PH should be carrying out stress-resistance and psychoemotional tests. It is necessary to involve psychotherapist in preventive maintaining in centers of student health [5] for more personalized prevention programs among young people.

**References**

1. Vasan RS, Larson MG, Leip EP, Kannel WB, Levy D. Assessment of frequency of progression to hypertension in nonhypertensive participants in the Framingham Heart Study: a cohort study. Lancet. 2001;358:1682-6.

2. O'Donnell M, Xavier D, Liu L, Zhang H, Chin SL, Rao-Melacini P, et al. Risk factors for ischaemic and intracerebral haemorrhagic stroke in 22 countries (the INTERSTROKE Study): a case-control study. Lancet. 2010;376:112-23.

3. Gryglewska B, Sulicka J, Fornal M, Wizner B, Cwynar M, Grodzicki T. Women with prehypertension in primary care – Risk profile on the basis of selected cardiovascular risk factors. Blood Press. 2009;3:99-104.

4. Knoflach M. Cardiovascular risk factors and atherosclerosis in young women. Atherosclerosis risk factors in female youngsters (ARFY Study). Stroke. 2009;40:1063-81.

5. Evsevyeva ME, Muravieva VN, Eremin MV, Galkova IY, Chudnovsky EV, et al. Student Health Center: the main areas of work at this stage. Prevent Med. 2013;1:8-12.

## A22 On the approaches to the early diagnosis of stress-induced hypertension in young employees of State law enforcement agencies

### Maria E. Evsevyeva, Ludmila V. Ivanova, Michail V. Eremin, Maria V. Rostovtseva

#### Stavropol State Medical University, Stavropol, Russian Federation

##### **Correspondence:** Maria E. Evsevyeva (evsevieva@mail.ru) – Stavropol State Medical University, Stavropol, Russian Federation

**Keywords:** Job stress, Young men, Arterial hypertension

**The actuality:** Now psycho-emotional stress (PES) is one of the leading risk factors of cardiovascular (CV) diseases, including arterial hypertension (AH) [1]. According to the international multicenter study INTERHEART (2004) PES is among the leading factors of CV risk, ranking third after smoking and dyslipidemia [2]. One variety of chronic PES is job stress (JS) [3], which is far more studied in the field of industry, transport [4], and much less studied in terms of law enforcement [5].

**Objective:** Estimation the features of circadian blood pressure (BP) profile in young men employed in the stress-associated field work of law enforcement.

**Material and methods:** A total of 132 young men, exposed to different severity of JS in type of operational activity (OA) of experience for 1 to 5 years were surveyed. Control group was formed of persons, whose daily work is not related with OA. Circadian monitoring (CM) BP was conducted on different days of the week, comparing results of the office and DMBP determining.

**Results:** It was revealed that the presence of different forms of stress-induced AH - stable, isolated office and hidden ones were significantly more often compared to the control group. The most significant violations identified by the time indices of increasing BP day and night, as well as the speed of morning rise BP. In output day, violations of DMBP leveled, that confirms the stressful nature of diagnosed AH.

**Conclusion:** It is proposed the use of CMBP during regular medical examinations of young men, exposed to the JS, in order to carry out differential diagnosis of various forms of stressful AH in a good time. This can be useful for the further conduct of individualized prevention programs [1] in the workplace.

**References**

1. Poghosova GV. Recognition of the importance of mental and emotional stress as cardiovascular risk factor of the first order. Cardiology. 2007;2:65-72.

2. Yusuf S, Hawken S, Ounpu S, Dans T, Avezum A, Lanas F, et al. Effect of potentially modifiable risk factors associated with myocardial infarction in 52 countries (the INTERHEART Study): case-control study. Lancet. 2004;364:937-52.

3. Karasek R, Theorell T. Healthy work: stress, productivity, and the reconstruction of working life.Basic Books. New York: The Perseus Book Group, 1990.

4. Kivimaki V, Leino-Arjas А, Luukkonen R, Riihimäki H, Vahtera J, Kirjonen J. Work stress and risk of cardiovascular mortality prospective cohort study of industrial employes. BMJ 2002;325:857-60.

5. Ivanova LV, Evsevyeva ME, Eremin MV, Rostovtseva MV, Orechova NV. Workplace arterial hypertension and tolerability of various diagnostic loads. Modern Problems Sci Educ. 2015;4.

## A23 Сentral aortic pressure and indexes of augmentation in young persons in view of risk factors

### Maria E. Evsevyeva, Michail V. Eremin, Vladimir I. Koshel, Oksana V. Sergeeva, Nadesgda M. Konovalova

#### Stavropol State Medical University, Stavropol, Russian Federation

##### **Correspondence:** Maria E. Evsevyeva (evsevieva@mail.ru) – Stavropol State Medical University, Stavropol, Russian Federation

**Keywords:** Persons of young age, Central аortic pressure, Risk factors

**The actuality:** The phenomenon of augmentation, that has important prognostic value [1], is studied basically on old contingent [2, 3]. At young it is investigated much worse [4].

**Objective:** Estimation central aortic pressure (CAP), index of augmentation (AIxао) and index of amplification (PPA) in young people, taking into account the presence of RF.

**Material and Methods:** 78 students were surveyed within the framework of the regular medical inspections on the basis of the University Center of Student's Health. Screenings included: RF - overweight, smoking, physical inactivity, anxiety/depression (A/D), infectious-inflammatory diseases (IID) most often in the form of pathology of LOR-organs, adverse heredity (AH), and arterial hypertension (AH). Parameters were studied CAP by diagnostic complex BPLab ('Peter Telegin', Russia). We provided systolic blood pressure (BP) aortic (SYSао), diastolic BP aortic (DIAао), index of augmentation in aorta (AIxао), pulse pressure amplification (PPA) and others. There were two groups: 1 g. – RF not present (control group); 2 g. - RF present (the basic group). Statistical processing was performed by means of package of programs «Statistica 8».

**Results:** Youth AIxao index in the two groups of observation was -8 (-12; 2 %) and -4 (-9; 2)% (p = 0.32), while among women the figure was 1.5 (-2, 5; 5) and 7 (2.5; 12)% (p = 0.04). Among young men in two groups PPA index was 148 (142; 158) % and 148 (137; 152) % (p = 0.79), while among women the figure was 140.5 (133; 152) and 140.5 (132; 145) % (p = 0.89).

**Conclusion:** Under the influence of the FR in young people there is an increase in AIxао. PPA remains almost unchanged. It identified gender-specific violations of the AIxао. Girls are characterized by increase of this index: almost six-fold, and the young men - only twice. These data should be considered when implementing the diagnostic screening of threats to cardiovascular health in young people [5], as well as in the process of monitoring the effectiveness of preventive measures among youth.

**References**

1. Laurent S, Katsahian S, Fassot C, Tropeano AI, Gautier I, Laloux B, et al. Aortic stiffness is an independent predictor of fatal stroke in essential hypertension. Stroke. 2003,34:1203–6.

2. Segers PN. Basic principles of wave reflection and central pressure. In: Laurent S, Cockroft J, editors. Central aortic blood pressure. Paris: Les Laboratoires Servier; 2008, p. 19-25.

3. Mancia G, Fagard R, Narkiewicz K, Redon J, Zanchetti A, Böhm M, et al. 2013 ESH/ESC Guidelines for the management of arterial hypertension: the Task Force for the management of arterial hypertension of the European Society of Hypertension (ESH) and of the European Society of Cardiology (ESC). Eur Heart J. 2013;34(28):2159-219. doi:10.1093/eurheartj/eht151

4. Laurent S, Cockcroft J, Van Bortel L, Boutouyrie P, Giannattasio C, Hayoz D, et al. Expert consensus document on arterial stiffness: methodological issues and clinical applications. Eur Heart J. 2006;27(21):2588-605.

5. Evsevyeva ME, Sergeeva OV, Niculina GP, Baturina MV, Rostovtseva МV, Naymanova ZN, et al. Ways of improving the medical examination of young individuals at risk for adulthood cardio-vascular diseases. Prevent Med. 2008;3:40-3.

## A24 Breast cancer prediction and prevention: Are reliable biomarkers in horizon?

### Shantanu Girotra (shantanugirotra@gmail.com), Olga Golubnitschaja

#### Department of Radiology, University of Bonn, Bonn, Germany

##### **Correspondence:** Olga Golubnitschaja (olga.golubnitschaja@ukb.uni-bonn.de) – Department of Radiology, University of Bonn, Bonn, Germany

**Keywords:** Predictive preventive personalized medicine, Breast cancer, Biomarker pattern, Patient stratification, Risk assessment, Non-invasive diagnostics, Multilevel diagnostics, Blood test, Screening program, Health economy

**Breast cancer burden**

Breast cancer (BC) is the most common cancer amongst women worldwide: annually about half a million deaths is reported [1]. First-line screening program is through mammography for which false-negative results, over-diagnosis and health adverse effects are not rare issues. Although treatment approaches consider the known BC relevant biomarkers (such as BRCA1/2, ER/PR, HER2, UPA, PAI-1, etc.), a large number of BC cases remains unrecognisable for currently applied screening programmes, early detection, correct diagnosis and prognosis, optimal treatment regiments such as triple-negative BC patients with overall poor outcomes.

**Cost-effective approach**

Well acknowledged modifiable risk factor in BC are: diet, physical activity, sleep quality, alcohol intake and psychological distress. The proposed cost-effective approach might be the paradigm shift from delayed to predictive, preventive and personalised BC management, which considers shifting focus from illness to health, innovative screening programs, regular monitoring of pre-disposed individuals and the ones a high risk, targeted preventive measures and treatments tailored to the individual.

PPPM in BC management can be effectively implemented by targeting the overall diagnostic and treatment approaches to well stratified patient groups.

**Patient stratification in BC management: creation of highly specific and sensitive biomarker-panels**

An optimal BC management considers the development of multilevel diagnostics and treatment regiments:

1. Innovative screening programs identifying individuals at risk and early-stage patients;

2. Stratification of BC patients predisposed to metastatic disease;

3. Creation of individualised patient profiles using specific biomarker sets;

4. Targeted preventive measures and treatment algorithms according to the individualised patient profiles;

5. Prognosis of the disease severity and treatment outcomes.

Any stage of the above consolidate approach requires highly specific and sensitive biomarker-panels to be created using medical imaging, in-silico diagnosis and bioinformatics. Innovative concepts and non-invasive blood tests are proposed [2-4].

**References**

1. Yeghiazaryan K, Cebioglu M, Braun M, Kuhn W, Schild HH, Golubnitschaja O. Noninvasive subcellular imaging in breast cancer risk assessment: construction of diagnostic windows. Personalized Med. 2011;8(3):321-30.

2. Braun M, Fountoulakis M, Papadopoulou A, Vougas K, Seidel I, Höller T, et al. Down-regulation of microfilamental network-associated proteins in leukocytes of breast cancer patients: potential application to predictive diagnosis. Cancer Genomics Proteomics. 2009;6:31-40.

3. Golubnitschaja O, Yeghiazaryan K, Costigliola V, Trog D, Braun M, Debald M, et al. Risk assessment, disease prevention and personalised treatments in breast cancer: is clinically qualified integrative approach in the horizon? EPMA J. 2013;4(1):6.

4. Debald M, Yeghiazaryan K, Cebioglu M, Kuhn W, Schild HH, Golubnitschaja O. “Suspect molecular signature” in blood as the indicator of undiagnosed breast cancer, cancer risk and targeted prevention. EPMA J. 2013;4(1):22.

## A25 Flammer Syndrome and potential formation of pre-metastatic niches: A multi-centred study on phenotyping, patient stratification, prediction and potential prevention of aggressive breast cancer and metastatic disease

### Olga Golubnitschaja^1^, Manuel Debald^1^, Walther Kuhn^1^, Kristina Yeghiazaryan^1^, Rostyslav V. Bubnov^2^, Vadym M. Goncharenko^2^, Ulyana Lushchyk^2^, Godfrey Grech^3^, Katarzyna Konieczka^4^

#### ^1^Breast Cancer Research Centre, University of Bonn, Bonn, Germany; ^2^Clinical hospital “Pheophania” of State Affairs Department, Kyiv, Ukraine; ^3^University of Malta, Msida, Malta; ^4^Department of Ophthalmology, University of Basel, Basel, Switzerland

##### **Correspondence:** Olga Golubnitschaja (olga.golubnitschaja@ukb.uni-bonn.de) – Breast Cancer Research Centre, University of Bonn, Bonn, Germany

**Keywords:** Predictive preventive personalised medicine, Cancer advancement, Breast cancer, Pre-metastatic niches, Metastatic disease, Flammer Syndrome, Hypoxic effect, Phenotyping, Patient stratification, Multi-centred study

Detailed autopsy findings demonstrate that the absolute majority of people are carriers of hardly detectable micro and asymptomatic tumour lesions which, however, not necessarily may progress into clinically manifested disease. Further, in case of manifested oncologic diseases, less than 1 % of all disseminated and circulated tumour cells have a potential to form secondary and distanced tumours (metastatic diseases) – the phenomenon known as the “metastatic inefficiency” [1]. In this context, the key question puzzling modern predictive preventive and personalised medicine is how to discriminate between those carriers who are predisposed to a disease manifestation / progression and “silent” carriers.

By evidence, both initial tumours and secondary metastases need a “fertile” microenvironment effectively supporting their growth and progression [2]. What are the mechanisms “fertilising” the microenvironment for a particularly effective cancer advancement? In general, these are local and systemic effects at molecular, cellular and tissue levels which create hospitable conditions for tumour and metastatic colonisation. Amongst pronounced risk factors hypoxia is recognised as a strong driver of aggressive cancer types and active metastatic disease, e.g. triple negative breast cancer. Systemic hypoxic effects have been demonstrated as forming pre-metastatic niches in distant organs [2,3].

Regarding specific phenotypes particularly predisposed to local and systemic hypoxic effects, individuals with Flammer Syndrome (FS) phenotype create prominent cohorts of healthy individuals in sub-optimal health condition [4] as well as patients suffering from severe diseases such as eye disorders [5,6]. In the above introduced context, FS individuals are of particular interest, due to

- clearly defined phenotype [4]
- onset of symptoms early in life (puberty)
- more frequent in young women
- systemic hypoxic/ischaemic effects
- involvement of systemic molecular events (altered stress response, multi-drug resistance and energy metabolism; shifted regulation of transcription, apoptosis and adhesion; deficits in DNA-repair efficacy; blood-brain-barrier-breakdown; extensive tissue remodelling accompanied by highly increased activity of the core of metalloproteinase) into pathogenesis of severe disorders in patients with FS phenotype [7, 8]. All these pathways are considered as evidently involved into effective cancer advancement [9].

Our multi-centred study has been designed to respond to the following questions:

1. Are women with FS phenotype more predisposed to cancer onset and progression compared to the general population?

2. Are women with FS phenotype more predisposed to breast cancer?

3. Which types of breast cancer are more frequent in women with FS phenotype?

4. Has FS phenotype a power to predict pre-metastatic niches in cancer patients?

5. Is patients’ stratification in cancer and metastatic disease possible by phenotyping with FS symptoms?

6. Is cancer prediction and prevention possible by FS phenotyping in predisposed individuals?

Our multi-centred consortium will, further, report on the results coming from this exciting study. A series of research articles is currently in preparation by the EPMA nominated working group.

**References**

1. Redig AJ, McAllister SS. Breast cancer as a systemic disease: a view of metastasis. J Intern Med. 2013;274(2):113–26.

2. Cox TR, Rumney RM, Schoof EM, Perryman L, Hoye AM, Agrawal A, et al. The hypoxic cancer secretome induces pre-metastatic bone lesions through lysyl oxidase. Nature. 2015;522:106–10.

3. Vanharanta S. A hypoxic ticket to the bone metastatic niche. Breast Cancer Res. 2015;17(1):122.

4. Konieczka K, Ritch R, Traverso CE, Kim DM, Kook MS, Gallino A, et al. Flammer syndrome. EPMA J. 2014;5(1):11.

5. Yeghiazaryan K, Flammer J, Orgül S, Wunderlich K, Golubnitschaja O. Vasospastic individuals demonstrate significant similarity to glaucoma patients as revealed by gene expression profiling in circulating leukocytes. Mol Vis. 2009;15:2339–48.

6. Flammer J, Konieczka K, Flammer AJ. The primary vascular dysregulation syndrome: implications for eye diseases. EPMA J. 2013;4(1):14.

7. Yeghiazaryan K, Flammer J, Golubnitschaja O. Predictive molecular profiling in blood of healthy vasospastic individuals: clue to targeted prevention as personalised medicine to effective costs. EPMA J. 2010;1(2):263–72.

8. Golubnitschaja O, Yeghiazaryan K, Flammer J. Key molecular pathways affected by glaucoma pathology: is predictive diagnosis possible? EPMA J. 2010;1(2):237–44.

9. Golubnitschaja O, Yeghiazaryan K, Costigliola V, Trog D, Braun M, Debald M, et al. Risk assessment, disease prevention and personalised treatments in breast cancer: is clinically qualified integrative approach in the horizon? EPMA J. 2013;4(1):6.

## A26 Innovative tools for prenatal diagnostics and monitoring: improving individual pregnancy outcomes and health-economy in EU

### Olga Golubnitschaja^1,2^, Jan Jaap Erwich^3^, Vincenzo Costigliola^2,4^, Kristina Yeghiazaryan^1,2^, Ulrich Gembruch^5^

#### ^1^Department of Radiology, University of Bonn, Bonn, Germany; ^2^European Association for Predictive, Preventive and Personalised Medicine (EPMA), Brussels, Belgium; ^3^University Medical Center Groningen, Groningen, The Netherlands; ^4^European Medical Association (EMA), Brussels, Belgium; ^5^Department of Gynaecology and Obstetrics, University of Bonn, Bonn, Germany

##### **Correspondence:** Olga Golubnitschaja (olga.golubnitschaja@ukb.uni-bonn.de) – Department of Radiology, University of Bonn, Bonn, Germany

**Keywords:** Prenatal diagnostics, Healthcare system, Economical burden, Predictive preventive personalised medicine

**EU birth rates in the global context**

In the world ranking for annual birth rates (from place 1 for Nigel till place 224 for Monaco with 46.12 *versus* 6.72 annual births, respectively), all the EU countries are positioned very low beginning with the highest rank 132 for Ireland (15.18 births/1000) and going deep into the lowest rank for 219 for Germany – position 6^th^ from the last place [1].

**Improving healthcare for and increasing life quality of pregnant women in EU**

From the above statistical data it is evident that the birth rates in the EU belong to the lowest ones worldwide. This fact motivates European Union for improving the healthcare for and increasing life quality of pregnant women, advancing the level of professional monitoring of pregnancy and delivery, and promoting the philosophy of predictive and preventive medical services and wellbeing in pregnancy with improved individual outcomes of delivery that is the main focus of our consortium.

**Epidemiology of prenatal / perinatal complications and “down-stream” pathologies**

Current statistical data considering worldwide epidemiology of prenatal and perinatal complications and pathologies have not been systematically analysed being frequently controversial for single countries. However, recent studies have demonstrated the prenatal and perinatal morbidity comprising the majority of the infant deaths giving a general idea of the biggest impact of prenatal and perinatal complications and reflecting extensive deficits in medical care as currently conducted to pregnant women. Further, for life births, when newborns were affected by prenatal / perinatal complications, individual outcomes are hardly estimable but the long-term consequences might be dramatic such as hypoxic-ischaemic encephalopathy, injuries of the central nervous system, epilepsy, strong predisposition to develop neurodegenerative and cardiovascular diseases, diabetes and cancer early in life [2-4].

**Economical burden to healthcare systems**

Already now the economical burden to healthcare systems is enormous for each of the above listed pathologies with a very pessimistic prognosis for the next decade in case of less improvement in medical services. Therefore, the quality of prenatal care is the central issue for entire progress in healthcare systems and economy to be made [5].

In the European and in high-income countries generally, the most of the severe complications during pregnancy still occur in a-priori low-risk pregnant women [6]. Consequently, the prioritised need is to develop affordable instruments to diagnose and monitor most important risk factors in order to prevent development of high blood-pressure, foetal growth restriction, preterm delivery leading to stillbirth or neonatal death. Our consortium is open for international multi-disciplinary collaborations which might create a series of projects required for effective advancements in the field.

**References**

1. [Birth rates]. http://www.laenderdaten.de/bevoelkerung/geburtenrate.aspx Accessed 15 Mar 2015

2. Golubnitschaja O, Yeghiazaryan K, Cebioglu M, Morelli M, Herrera-Marschitz M. Birth asphyxia as the major complication in newborns: moving towards improved individual outcomes by prediction, targeted prevention and tailored medical care. EPMA J. 2011;2(2):197–210.

3. Peeva V, Yeghiazaryan K, Golubnitschaja O. Birth asphyxia as the most frequent perinatal complication. In: Predictive diagnostics and personalized treatment: Dream or Reality. Golubnitschaja O (ed), ISBN 978-1-60692-737-3, Nova Science Publishers, New York, USA, 2009.

4. Yeghiazaryan K, Peeva V, Morelli M, Herrera-Marschitz M, Golubnitschaja O. Potential targets for early diagnosis and neuroprotection in asphyxiated newborns. In: Predictive diagnostics and personalized treatment: Dream or Reality. Golubnitschaja O (ed), ISBN 978-1-60692-737-3, Nova Science Publishers, New York, USA, 2009.

5. Healthcare Overview: New Perspectives, Ed.: V. Costigliola, in Book Series “Advances in Predictive, Preventive and Personalised Medicine”, Golubnitschaja O. (Series Editor), Springer Dordrecht Heidelberg New York London, V.1, 2012 ISBN 978-94-007-4602-2

6. Goldenberg RL, McClure EM, Bhutta ZA, Belizán JM, Reddy UM, Rubens CE, et al. Stillbirths: the vision for 2020. Lancet 2011;377(9779):1798–805.

## A27 Immunohistochemical assessment of APUD cells in endometriosis

### Vadym M. Goncharenko (dr.v.goncharenko@gmail.com)^1^, Vasyl O. Beniuk (benyuk@i.ua)^2^, Olga V. Kalenska (OVKalenska@ukr.net)^1^, Rostyslav V. Bubnov^1^

#### ^1^Clinical Hospital ‘Pheophania’ of State Affairs Department, Zabolotny str., 21, Kyiv 03680, Ukraine; ^2^Bogomolets National Medical University, Kyiv 01601, Ukraine

##### **Correspondence:** Rostyslav V. Bubnov (rostbubnov@gmail.com) – Clinical Hospital ‘Pheophania’ of State Affairs Department, Zabolotny str., 21, Kyiv 03680, Ukraine

**Keywords:** Predictive preventive personalized medicine, Participating medicine, Endometriosis, APUD cells, Serotonin

**Introduction:** Endometriosis is among leading gynecological pathologies, amine precursor uptake and decarboxylation (APUD) cells still were not sufficiently studied in this matter.

The aims were to conduct immunohistochemical assessment of diffuse endocrine system (APUD) cells in uterus endometriosis (adenomyosis) and ovarian endometriosis.

**Materials and Methods:** We included 25 patients with uterine endometriosis (mean age 44.2 ± 1.67), and 19 patients with ovarian endometriosis (39.4 ± 1,82 years old). The control group included 15 women (38.6 ± 1.54 years old) with no gynecological pathology. Fragments of the myometrium with endometriosis, endometrioid ovarian cysts walls, endometrium samples were assessed using immunohistochemical study (murine monoclonal antibody serotonin Ab-1, ClonDesignation 5HT-H20S). As the imaging system we used UltraVisionQuantoDetectionSystemHRPDAB (Thermoscientific). APUD cells were counted in 10 fields at average magnification x280.

**Results:** The APUD cells were found in 18 patients in the adenomyosis and in 16 patients with ovarian endometrioid cysts. In all patients APUD cells were also found in the endometrium. In the control group few APUD cells were observed in the endometrium (4-6 in 10 fields of view, x280). In the endometrium of patients with adenomyosis we found 5.6 APUD cells in 10 views; in the endometrium of patients with endometrioid cysts - 22.4 ± 1.68, significantly increased vs the control group (p <0.01) and vs group of adenomyosis patients (p <0.01). In the endometrioid ovarian cysts walls we defined 28.6 ± 1.72 APUD cells, significantly higher than in the foci of adenomyosis (p <0.05). APUD cells had different shapes, mostly irregular, were identified to produce serotonin.

**Conclusions:** We have revealed the reliable increase of APUD cells in ovarium endometriosis as in locus of endometriosis, so and in endometrium of this patients compared to normal endometrium of patients with adenomyosis.

**Outlook and Expert recommendations:** Neuroendocrine, APUD cells signaling and serotonin are important and not sufficiently studied mechanisms for number of pathologies of different localization, and link amongst series of pathological processes including obesity, CVD, cancer, etc. Serotonin is a primal signaling molecule that is implicated in the control of energy balance.

There should be a sufficient evidence study to determine relationships among APUD cells signaling and serotonin with endometrial receptor system, genetics, microbiome, immune pathways, apoptosis and vascular patterns to complement the diagnostic algorithm that will allow the development of novel treatments and model-guided approach.

## A28 Updating personalized management algorithm of endometrial hyperplasia in pre-menopause women

### Vadym M. Goncharenko (dr.v.goncharenko@gmail.com)^1,2^, Vasyl O. Beniuk (benyuk@i.ua)^1^, Rostyslav V. Bubnov^2,3^, Olga Melnychuk (Melnichukolga@inbox.ru)^1^

#### ^1^Bogomolets National Medical University, Kyiv 01601, Ukraine; ^2^Clinical Hospital ‘Pheophania’ of State Affairs Department, Zabolotny str., 21, Kyiv 03680, Ukraine; ^3^Zabolotny Institute of Microbiology and Virology, National Academy of Sciences of Ukraine, Zabolotny Str., 154, Kyiv 03680, Ukraine

##### **Correspondence:** Rostyslav V. Bubnov (rostbubnov@gmail.com) – Zabolotny Institute of Microbiology and Virology, National Academy of Sciences of Ukraine, Zabolotny Str., 154, Kyiv 03680, Ukraine

**Keywords:** Predictive preventive personalized medicine, Endometrium hyperplasia, Receptor biology, Ultrasound, Hysterescopy

**Introduction:** Endometrium hyperplasia (EH) is of huge importance in gynaecological morbidity. The debatable nature of aetiology pathogenous moments, insufficient classifications, the risk of changes leading to cancer and cancer of endometrium determine the topicality of this problem for women, those of the pre-menopause age in particular [1-3].

**The aim was** to update the EH treatment effectiveness via predictive diagnosis and personalized pathogenesis-based treatment algorithm in pre-menopause age patients.

**Materials and Methods:** We included to the study 161 women with the EH (age from 45 to 56, the average age 49.3 ± 2.3 tears), the patients of clinical hospital “Pheophania” and Kyiv Maternity Hospital N3.

Patients underwent clinical examination, the diagnostic hysteroscopy with the obligatory cervical channel and uterus cavity scraping off, and following histological diagnosis verification and the endometrium receptors phenotype identification.

Endometrium hysteroscopic ablation was performed using «KarlStorz» hysteroresectoscope (Germany). The transvaginal ultrasound was performed twice, after 1, 3 and 6 months after surgical intervention.

In order to define the differentiated tactics for the patients’ treatment, the age, EH form and the presence of the collateral somatic and genital pathology were taken to consideration.

**Results:** The investigation of reproductive system’s organs of women with EH in pre-menopause defined the following frequency of the gynecological pathology: fibroid tumor - 54 (33.5 %) patients, adenomyosis - 32 (19 %) patients, breast - 28 (17.3 %) women. The analysis of the extra genital pathology structure of women with EH stated the following frequency of the cardiovascular system disorders 62 (38.3 %), hepatic biliary system diseases - 39 (24.2 %) and neuro neuroendocrine disorders - 56 (34.7 %) patients.

While developing the differentiated treatment program, we considered the age of a woman, endometrial histological peculiarities (including the receptor phenotype, presence of the accompanying somatic and genital pathology); it provides one with the possibility to single out the patients who have the contra-indication to hormonal therapy and whom the surgical intervention is likely to be recommended.

EH is accompanied by the receptors quantitative and qualitative features which are worth taking into account while developing the personalized tactics of treatment, if ER/PR ≤ 1.0, the progesterone drugs therapy is preferable. In case the ER/PR correlation is over 1.0 and in case of receptors to progesterone decrease, GTRG are to be used for menolipsis and physiological correlations of receptors systems renovation.

All this data allowed to distinguish group of patients with contra-indication to progestin hormone therapy, the group of patients who were prescribed aGTRG antagonists or surgical teatment (intervention). In case of contra-indications to hormonal therapy and EH relapse’s development without atypical features, women of pre-menopause age should undergo the endometrium hysterescopic ablation which is the alternative to both continuous hormonal and radical surgical intervention.

**Conclusions:** Appropriate assessment in pre-menopause women with endometrial hyperplasia the broad panel of diagnostic and predictive markers, including analysis of receptor phenotype, somatic and genital pathology, planning surgery allows suggesting effective algorithm of personalized treatment.

**Outlook and Expert recommendations:** An international women's health project including the study of integrative diagnosis ovaries, endometrial pathology, cervix, breast a in regards to health aging for stratification patients for personalized therapies; organize educational programs should be created. There should be a sufficient evidence study to determine relationships in endometrial receptor system, genetics, microbiome, immune pathways to complement the diagnostic algorithm that will allow the development of novel treatments and model-guided approach.

**References**

1. Goncharenko VM, Beniuk VA, Kalenska OV, Demchenko OM, Spivak MY, Bubnov RV. Predictive diagnosis of endometrial hyperplasia and personalized therapeutic strategy in women of fertile age. EPMA J. 2013;4:24.

2. Goncharenko VM, Beniuk VA, Vyniarskyi YM, Bashynskyi SM, Bubnov RV. Assessment of endometrial receptor systems for PPPM approach for endometrial hyperplasia in reproductive age women. EPMA J. 2014;5(Suppl 1), A39. doi:10.1186/1878-5085-5-S1-A39

3. Goncharenko VM, Beniuk VA, Vyniarskyi YM, Bashynskyi SM, Bubnov RV. Personalized treatment strategy for atypical endometrial hyperplasia with regards to age, comorbidities and endometrial receptor status. EPMA J. 2014;5(Suppl 1):A40. doi:10.1186/1878-5085-5-S1-A40.

## A29 The personified treatment approach of polimorbid patients with periodontal inflammatory diseases

### Irina A. Gorbacheva, Lyudmila Y. Orekhova, Vadim V. Tachalov

#### St. Petersburg Periodontal Center “PAKS”, Pavlov First Saint Petersburg State Medical University, Saint Petersburg, Russia

##### **Correspondence:** Vadim V. Tachalov (tachalov@mail.ru) – St. Petersburg Periodontal Center “PAKS”, Pavlov First Saint Petersburg State Medical University, Saint Petersburg, Russia

**Keywords:** Polimorbid patients, Periodontal diseases, Metabolic drugs

Now the set of certificates on interrelation of inflammatory diseases of parodont (IDP) and various internal diseases are saved up. Universal molecular and cellular deviations, violations of energetic metabolism, pathological activation of apoptosis, immuno-pathological reactions, disbalance of presence of the major macro - and microelements in various biological environments, supplementing each other in the form of self-sustaining system, promote development and progressing of many somatic diseases and IDP associated with them. The interconnected pathogenetic links of the polimorbid diseases can be united in a polimorbid continuum. Results of our research works at the polimorbid patients showed increased activity of free radical oxidation of lipids and tiols, high level of markers of an early stage of pathological apoptosis of cellular receptors of CD 95+ (р< 0.01), reduction of activity of macrophages, high level of the circulating immune complexes in blood (р< 0.01) and disbalance of macro - and microelements- magnesium, calcium, copper, zinc, iron in the bioenvironments of an organism.

Multi-purpose monotherapy of the polimorbid patients is a kind of perspective treatment by the usage of one medicine for simultaneous correction of the broken functions of several systems. The special attention is paid to the metabolic drugs with the universal complex of mechanisms, and possessing metabolic, antioxidant and antiapoptotic action. Such effects were established using a сerebrolysin, cytoflavin, mexidol, mildronate, cycloferon. The choice of a medicine must be motivated by the account of the prevailing pathology in a polimorbid complex and indications to application of the chosen mean of multimodal therapy. Such tactics provide a personified approach to the appointed treatment.

**Conclusions**

1. Polimorbid patients with IDP need the complex inspection including an assessment of an oxidizing stress, immunological shifts and balance of macro - and microelements.

2. The polimorbid patients with the combined IDP treatment must include the means of multi-purpose pathogenetic monotherapy.

3. Interdisciplinary aspects of pathogenesis of IDP demand cooperation of dentists and internist for the joint maintaining of the polimorbid patients.

## A30 Ukrainian experience in hybrid war – the challenge to update algorithms for personalized care and early prevention of different military injuries

### Olena I. Grechanyk^1^, Rizvan Ya. Abdullaiev (r.abdullaev@bk.ru)^2^, Rostyslav V. Bubnov (rostbubnov@gmail.com)^3^

#### ^1^Main Ukrainian Military Clinical Hospital #17 in Kyiv, Gospitalna str., 18, Kyiv, Ukraine; ^2^Kharkiv Medical Academy of Postgraduate Education, Kharkiv, Ukraine; ^3^Clinical Hospital “Pheophania” of State Affairs Department, Zabolotny Str., 21, Kyiv 03680, Ukraine

##### **Correspondence:** Olena I. Grechanyk (greshanyk@gmail.com) – Main Ukrainian Military Clinical Hospital #17 in Kyiv, Gospitalna str., 18, Kyiv, Ukraine

**Keywords:** Military medicine, Clinical data, Medical records, Personalization, Replacement technology, Rehabilitation centers

**Introduction:** The era of local wars greatly increased incidence of mine-explosive wounds (MEW): they accounted for 13 % up to 76 % in war history. While ensuring antiterrorist operation (ATO) we observe the growing number of wounded servicemen with mine-explosive wounds, affecting many areas of the body and develops post-contusion-commotion syndrome, leg injuries. Use of kevlar helmets, body armor, in many cases saving the head, chest, so the fore limb damage. Injuries of limbs in the structure of surgical sanitary losses prevail over the other injuries, many of the wounded are amputees and suffer from severe infections, including gangrene.

The aim was to analyze the structure of radiology / ultrasound diagnosis at tertiary level military clinic during the anti-terrorist operation (ATO) in the East of Ukraine.

**Materials:** We analyzed clinical records of wounded patients, who underwent examination in Ultrasound department of Ukrainian Military Clinical Hospital working in enhanced mode during the Ukrainian Revolution of Dignity and ATO (January 2014 to January 2015).

Ultrasound was conducted in 2789 patients, among them 1305 (48 %) patients with mine-explosive wounds, with different nature of injuries. The average age of injured males surveyed amounted 37.9 ± 13.6 years. When screening via ultrasound survey patients were divided into two groups: surgical patients in 1345 individuals (48 %), affected therapeutic profile in 1444 patients (52 %). According to localization in anatomical and topographical sections lesions wounded were divided into 5 groups: head and neck (group 1; 22 %), chest and upper limbs (group 2; 13 %), abdomen (group 3; 31 %), bowel and the lower extremities (group 4; 34 %).

X-ray, CT, MRI were used for diagnosis cranial, orbital pathology, to identify anatomical and topographical location of foreign bodies (shrapnel, bullets, drainage, tampons, various fragments of shells). The initial stage of diagnosis algorithm of wounds was ultrasound.

**Results and Discussion:** According to our records 58 % of injuries were combine damages of liver, spleen, kidneys, retroperitoneal, subcapsular organic and extraorganic hematomas, hemotorax, hemoperitoneum, hemoscrotum, hemophthalmia that in general was gunshots wounds prevailed in the structure of all damages and is 69 %. Organs contusion induced splanchnomegalia and enhanced vascularization.

57 % of surveyed patients of therapeutic profiles had acute reactive states, neurological, angio-neurological, neurosurgical conditions. In 53 cases with blunt head trauma (closed head injury, concussion, acute acoustic trauma, wounds coal face, scalp, concussion) we registered reduction of blood flow in the vertebral arteries.

The injuries of extremities ranged over 65 %. Deep veins thrombosis (DVT) of the lower extremities was found in 45 (32 %) patients with mine-explosive wounds, asymptomatic DVT was diagnosed using Doppler ultrasound. We successfully used share wave sonoelastography (SEG) to study the elasticity of thrombus. Thus, the average values of the clot elasticity "fresh" blood clots, blood clots with high risk for embolization was 1.39 ± 1,14 kPa; for blood clots boundary degree of risk of embolization – 2.89 ± 0.50 kPa; for "consolidated" blood clots – 7.82 ± 0.39 kPa. In 29 (40 %) of cases we detected changes in soft tissies on SEG that were not detected in the gray scale mode, as an intermediate type mapping (yellow-red staining, Young modulus was 98.8 ± 45.1 kPa), indicating that intramuscular hematoma with areas of fibrosis, scars to replace the tissue gaps.

**Conclusions:** Analyzing the large clinical data we observed the prevailing of gunshots, shrapnel wounds, mines and explosive damage of any affected multiple parts of the body. When using personal protective equipment limb injuries are dominant. Ultrasound is the primary method algorithm for screening and diagnosis of traumatic injuries. The use of Doppler and sonoelastography of extremities allows to diagnose asymptomatic thrombotic complications in peripheral vessels at different times after injury.

**Outlook and Expert recommendations:** Antiterrorist operation (ATO) provided large clinical data with systemic medical records collection for analysis and research to predict and prevent injuries in the future. Early measures (innovative first aid and wound care) to prevent amputations and complication are needed to be implemented. Developments of rehabilitation centers network, and further socialization using broad volunteer activity is an urgent need. Delivery of personalized prosthesis and replacement technologies via multidisciplinary smart solutions is strongly needed (e.g., using 3D modeling, 3D printing, etc.) for improvement of quality of life of patients.

## A31 Tear fluid biomarkers: a comparison of tear fluid sampling and storage protocols

### Suzanne Hagan, Eilidh Martin, Ian Pearce, Katherine Oliver

#### Vision Sciences, School of Health and Life Sciences, Glasgow Caledonian University, Glasgow, Scotland, UK

##### **Correspondence:** Suzanne Hagan (Suzanne.Hagan@gcu.ac.uk) – Vision Sciences, School of Health and Life Sciences, Glasgow Caledonian University, Glasgow, Scotland, UK

**Keywords:** Biomarker, Tear fluids, Sample storage, Standard operating procedure, Multiplex arrays

**Purpose:** In recent years, multiplex microbead technology has been increasingly used to identify novel biomarkers of ocular surface disease via tear fluids. These studies, however, have also highlighted differences in tear fluid sampling across laboratories. We have performed a standard operating procedure (SOP) to optimise tear fluid retrieval and storage methods, versus cytokine expression, using magnetic multiplex bead arrays.

**Methods:** Pooled tear fluid samples underwent microarray bead analysis for 7 cytokines (IL1-β, IL-2, IL-6, IL-8, IL-17A, IFN-γ and TNF-α) using various tear sampling and storage techniques. A standard method of using 1ul tears in a 50x dilution was used throughout the study, except when looking at the effect of different tear volumes on cytokine detection.

**Conditions Tested**

Lo-Bind eppendorfs versus standard microfuge tubes

Multiple freeze/thaw cycles

Retrieval: Schirmer strip versus microcapillaries versus minisponges

Different tear volumes (1μl, 3μl, 5μl)

Sample storage (-20 °C versus -80 °C)

**Results:** Multiple freeze/thaw cycles showed significantly reduced levels of IL1-β and -2 and showed a trend for a reduction in the other 5 cytokines. Levels of IL1-β and TNF-α were significantly diminished for tears stored in standard eppendorf tubes, versus Lo-Bind eppendorfs. Moreover, IL-2, -6, and -17 were significantly reduced in tears collected with a Schirmer strip versus a microcapillary. There were significant reductions in IL-8 and IFN-γ in tears retrieved using a minisponge versus microcapillaires. No significant difference was observed for tears stored at -20 °C versus -80 °C. A general trend for reduced cytokine levels was observed as tear sample volumes increased (1 μl versus 3 μl and 5 μl). This reduction was found to be significant when comparing 1 and 5 μl volumes for IL1-β and 17A.

**Conclusions:** Differences in tear fluid recovery and sample storage may affect cytokine detection by multiplex arrays. This may be due to sample dilution and inherent matrix effects, varying degrees of protein binding affinities of lab plastics used for storage, and sample sublimation. A standardised operating procedure for tear fluid retrieval and storage would benefit future studies of cytokine expression in dry eye disease. It is anticipated that the preliminary data generated here will serve to inform a more standardised protocol across laboratories.

## A32 The correlation of dietary habits with gingival problems during menstruation

### Cenk Haytac^1^, Fariz Salimov^1^, Servin Yoksul^1^, Anatoly A. Kunin^2^, Natalia S. Moiseeva^2^

#### ^1^Cukurova University, Faculty of Dentistry, Adana, Turkey; ^2^Voronezh N.N. Burdenko State Medical University, Voronezh, Russia

##### **Correspondence:** Cenk Haytac (cenkhaytac@cu.edu.tr) – Cukurova University, Faculty of Dentistry, Adana, Turkey

**Keywords:** Gingivitis, Inflammation, Menstruation, Nutrition

**Aim:** Some women experience inflamed and bleeding gingival problems and aphteous stomatitis due to hormonal imbalance during menstrual cycle. The sex hormones such as estrogene and progestrone affect angiogenesis which eventually increases vascularization of gingival tissues. Since not all women have these problems, some cofactors should be investigated as etiologic factors. Therefore, the aim of this study was to analyze the effects of dietary habits on gingival changes during menstruation.

**Material and Methods:** 30 women aged between 18 and 25 on the 1st and 3rd day of menstruation were included in this study. The plaque index and gingival index scores were recorded. The women also filled the questionarre of ‘Healthy Eating Index” forms and possible correlations were statistically analyzed.

**Results:** Overall scores of ‘HEIndex and gingival inflammation were not correlated. However, the analysis of each food sub-group showed that fruit consumption was negatively correlated with plaque and gingival index, while ‘empty calorie consumption’ was positively correlated with gingival inflammation(p < 0.05).

**Conclusion**: Dietary consultations can be added to routine periodontal treatment for better therapy outcomes.

## A33 Genomic medicine in a contemporary Spanish population of prostate cancer: our experience

### Bernardo Herrera-Imbroda (ber.urologia@gmail.com)^1^, Sergio del Río-González (srioglez@gmail.com)^1^, Maria Fernanda Lara^1^, Antonia Angulo (nangulo@myriad.com)^2^, Francisco Javier Machuca Santa-Cruz (machuca29018@gmail.com)^1^

#### ^1^Intercenter Urology Unit, Virgen de la Victoria University Hospital, Málaga, Spain; ^2^Myriad Genetics España S.L.U, Madrid, Spain

##### **Correspondence:** Maria Fernanda Lara (fer76lc@hotmail.com) – Intercenter Urology Unit, Virgen de la Victoria University Hospital, Málaga, Spain

**Keywords:** Prostate cancer, Personalized Medicine, CCP score, Prolaris

**Background/Objective:** Prostate cancer (PCa) is a major health problem worldwide due to its prevalence, morbidity and resource management associated. The applicability of personalized medicine will improve the patient and socio-economic management of this disease.

Prolaris is a novel biomarker for PCa used as an oncogenomic test, which has been validated retrospectively in over 8000 patients as prognostic and predictor factor for cancer-specific mortality and metastatic disease [1, 2].

Objective: To assess the usefulness of Prolaris to enhance the heterogeneity of the current risk groups and the impact on the personalized management of localized prostate cancer (LPCa).

**Methods:** A Spanish cohort of 284 LPCa patients (2013-2014) were analyzed and Prolaris was applied. Sections (2-4 mm) of tumor tissue samples embedded in paraffin were obtained from biopsies and sent to Myriad Genetics to apply the Prolaris test. Age, PSA, Gleason, number of biopsy cylinders, percentage of tumor affected and clinical state (cT) from patients were recorded. Bio-bank and local ethic committee approved informed consent were signed by all patients.

AUA tumor risk (PSA, Gleason and cT) was estimated by comparing it with the risk provided by the expression analysis (Prolaris-CCP score). Inferential analyzes were performed using Chi-square.

**Fig. 1 (abstract A33).**
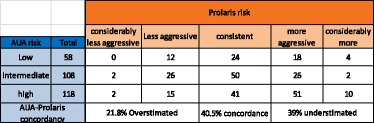
Tumor aggressiveness by AUA and Prolaris risk.

**Results:** The comparative analysis between AUA risk and Prolaris risk showed only a concordance of 40.5 % in the overall series, 41.3 % for low-risk tumors, 46.9 % for intermediate risk tumors and 34.7 % for high-risk tumors (Fig. 1, p <0.05). According to these results, the application of the parameters used in routine clinical practice would incur in an underestimation of the risk of 39 % and an overestimation of 20.1 %.

**Conclusions/Recommendations:** Individualized risk assessment of LPCa by Prolaris test allows better accuracy discrimination of tumor heterogeneity of the different actual risk groups. The clinical applicability of omics tools could improve our strategies in the selection of patients for different therapeutic modalities.

**Competing interests**

This study was funded by Myriad Genetics. A. Angulo works in Myriad Genetics. The remaining authors declare no conflict of interest.

**References**

1. Cuzick J, Swanson GP, Fisher G, Brothman AR, Berney DM, Reid JE, et al. Prognostic value of an RNA expression signature derived from cell cycle proliferation genes in patients with prostate cancer: a retrospective study. Lancet Oncol. 2011;12(3):245-55.

2. Cuzick J, Berney DM, Fisher G, Mesher D, Møller H, Reid JE, et al. Prognostic value of a cell cycle progression signature for prostate cancer death in a conservatively managed needle biopsy cohort. Br J Cancer. 2012;106(6):1095-9.

## A34 Challenges, opportunities and collaborations for personalized medicine applicability in uro-oncological disease

### Bernardo Herrera-Imbroda (ber.urologia@gmail.com), Sergio del Río-González (srioglez@gmail.com), Maria Fernanda Lara

#### Intercenter Urology Unit, Virgen de la Victoria University Hospital, Málaga, Spain

##### **Correspondence:** Maria Fernanda Lara (fer76lc@hotmail.com) – Intercenter Urology Unit, Virgen de la Victoria University Hospital, Málaga, Spain

**Keywords:** Multidisciplinary approach, Uro-oncological disease, Personalized medicine

**Background:** Our unit consists of three centers attending a health area of over 1 million people; therefore our ability to recruit and include patients in projects is very high.

The unit has previous experience in the development of studies related to molecular oncology, new biomarkers and personalized medicine. Our unit is included within the Biomedical Research Institute of Malaga, and has a laboratory of oncology and molecular imaging (NGS, Ncounter, PCRreal-time, isoflux-CTCs, cDNA, PET-MRI3T Fusion).

**Objectives:** Prostate cancer (PCa) is a major health problem worldwide due to its prevalence, morbidity and resource management associated. Bladder cancer (BC) is the most expensive oncological disease due to the chronicity of non-muscle-invasive tumors, the radical treatments and the systemic therapies for infiltrative disease.

Our main activities in the uro-oncology are focus on:

**i) PCa:** The overall aim is to develop and assess a personalized medicine program on localized PCa intended for the detention of clinical significant and non-significant tumors as well as to establish at-risk-population profiles based on the combination of omic tools, genetic, epigenetic, preventive, risk factors and lifestyles, as compared with the parameters of conventional clinical practice.

The results will enable to adopt novel preventive and therapeutic recommendations within the health system with the aim of decreasing over diagnosis and overtreatment on non-significant PCa; selecting suitable candidates for initial curative-intended treatments; decreasing morbimortality in additional procedures; improving the quality of life and psychosocial effects derived from unnecessary treatments; and readdressing policies leading to an improvement in health as well as to the optimization of social, health and economic resources.

**ii) BC:** There are several patients susceptible to high cancer-specific mortality, in which it is crucial to incorporate new predictive and prognostic diagnostic tools and to identify new targets that establish new treatments or optimize the current one, in order to improve survival.

The overall aim of our research is to establish a new taxonomy in bladder cancer by comprehensive analytical strategy of various molecular pathways in order to identify candidates for new-targeted therapies and implementing them in clinical practice.

**Conclusions:**

PCa and BC are two oncological diseases that may benefit of novel therapeutic strategies; many of them will derive from the applicability of the omics sciences and personalized medicine. Therefore, it is important to incorporate new models of health-care based on individualized risk profiles and to establish indicators and evidence of the suitability of these models of personalized medicine to be incorporated into health policies.

## A35 Metabolic hallmarks of cancer as targets for a personalized therapy

### John Ionescu (John.Ionescu@gmx.com)^1,2^

#### ^1^Spezialklinik Neukirchen, Neukirchen beim Heiligen Blut, Germany; ^2^Donau University Krems, Krems, Austria

**Keywords:** Cancer, Integrative therapy, Life quality, Predictive preventive personalized medicine

Redox and pH reactions involve electron and proton transfers in chemical and biological systems, whereby deviations from normal values are defined as oxidosis/redosis and acidosis/alkalosis, respectively. Cancer cells are depicting a constant redosis state with an increased accumulation of reduced glutathione, NADH, NADPH, cysteine, glucose and transitional metals, respectively. Consistent with their redosis state, proliferating cancer cells show a permanent intracellular alkalinization (pHi 7.12-7.65) when compared to normal cells (pHi 6.99-7.20) related to an obvious HIF1 and aerobic glycolysis activation, known since 1930 as “Warburg effect”.

The constant intracellular alkalinization of proliferating cancer cells is closely related to a highly increased activity of the Na^+^/H^+^ Antiporter system, of the V-ATP-ase proton pumps and of the MCT lactate transporters, all of them leading to a continuous excretion of protons (H^+^) and lactate in the extracellular milieu. Zn-dependent carbonic anhydrases (CA2, CA9, CA12) are also contributing to the acidification of the extracellular tumor environment (pHe 6.2-6.9) when compared to normal tissue (pHe 7.3-7.4). This acidic milieu is promoting both the tumor growth with metastatic spreading and the inhibition of immune competent cells.

The above-mentioned molecular-biological hallmarks gain an important role in the diagnosis and treatment of therapy-resistant neoplastic disorders. New integrative therapy approaches with significant antiproliferative and pro-apoptotic effects may include:

(1) The elimination of the intracellular redosis state by means of pro-oxidative approaches,

(2) The use of i.v. basic solutions for buffering the extracellular acidic state,

(3) The usage of high-dose Vitamin C and polyphenolic compounds leading to in situ high ROS generation in the presence of metal-rich tumor cells,

(4) Pharmacologic inhibition of proton pumps, Na^+^/H^+^ antiporter systems and carbonic anhydrases,

(5) The choice of appropriate diet forms with low glycemic index and high ketogenic and pro-oxidative properties and

(6) The inhibition of key enzymes of aerobic glycolysis.

These therapeutic interventions alone, or in combination with classic oncology treatments can significantly increase the lifespan and life quality.

## A36 Influence of genetic polymorphism as a predictor of the development of periodontal disease in patients with gastric ulcer and 12 duodenal ulcer

### Alfiya Z. Isamulaeva^1^, Anatoly A. Kunin^2^, Shamil Sh. Magomedov^3^, Aida I. Isamulaeva^4^

#### ^1^Department of Therapeutic Dentistry Medical University "Astrakhan State Medical Academy" Russian Ministry of Health, 121 Bakinskaya St., Astrakhan, 414000, Russia; ^2^Faculty of Dentistry Medical University "Voronezh State Medical Academy" of the Ministry of Health of the Russian Federation n.a. N.N. Burdenko, Institute of Dentistry at VGMA n.a. N.N. Burdenko, 14 pr. Revolyutsii, Voronezh, 394000, Russia; ^3^Department of Therapeutic Stomatology, Medical University "Astrakhan State Medical University" the Ministry of Health of Russia, 121 Bakinskaya St., Astrakhan, 414000, Russia; ^4^Faculty of Dentistry Medical University "Astrakhan State Medical Academy", the Ministry of Health of Russia, Russia, 121 Bakinskaya St., Astrakhan, 414000, Russia

##### **Correspondence:** Alfiya Z. Isamulaeva (annaviktorovna-8@yandex.ru) – Department of Therapeutic Dentistry Medical University "Astrakhan State Medical Academy" Russian Ministry of Health, 121 Bakinskaya St., Astrakhan, 414000, Russia

**Keywords:** Рeriodontal disease, Gene polymorphism, Predictive preventive personalized medicine

The study of the genetic basis of periodontal lesions on the background of peptic ulcer is an urgent task. Identification of genetic systems, their pleiotropic effects will clarify the genetic basis of periodontal disease and closer to the mechanisms of interaction of polygenic systems implementation of genetic information at the level of the whole organism [1-3].

The objective was to analyze the association of clinical manifestations with polymorphic variants of genes IL-1β and IL-1RN on the course of periodontal disease.

We examined 100 patients with periodontal disease, comorbidity of the gastrointestinal tract and 117 healthy blood donors. Using molecular genetic methods based on DNA extraction, polymerase chain reaction, restriction analysis, the method of polyacrylamide gel electrophoresis were identified polymorphisms T511C (+3953) A1/A2 in the gene IL-1β and VNTR polymorphism in the gene antagonist of IL-1.

Patients revealed the association 3953 polymorphism A1/A2 gene IL-1b, what proved the influence of genotype A1/A2(tt) and A2/A2(tt) on the severity of periodontitis. Patients with different genotypes for the polymorphism 511(C/T) gene IL-1b did not differ in frequency of inheritance of gastric ulcer and 12 duodenal ulcer (χ2 = 0.065; df = 2; p = 0.45). A similar trend was observed when comparing the allele (χ2 = 0.052; df = 1; p = 0.77). In patients with different genotypes and alleles in VNTR polymorphism of the gene IL-1Ra also did not differ in the frequency of occurrence compromised hereditary history for the development of somatic pathology by comparing allele (χ2 = 0.329 and χ2 = 0.028, p > 0.05, respectively).

The study to the association of the VNTR polymorphism of the gene IL-1Ra with the development of the disease, proved the influence of A1/A2, A2/A2 on the severity of inflammatory and destructive processes in the periodontium.

This polymorphism as a predictor of the development of severity, predict the dynamics of periodontal disease, contributing to the justification of a predictive, preventive and personalized medicine [4, 5]. The use of targeted immune will lead to a pathogenetic approach in the treatment of periodontal lesions.

**References**

1. Buduneli N, Kinane DF. Host-derived diagnostic markers related to soft tissue destruction and bone degradation in periodontitis. J Clin Periodontol. 2011;38:85–105.

2. El-Omar EM, Carrington М, Chow WH, McColl KE, Bream JH, Young HA, et al. Interleukin-1 polymorphisms associated with increased risk of gastric cancer. Nature. 2000;404:398-402.

3. Hasturk H, Kantarci A, VanDyke TE. Oral inflammatory diseases and systemic inflammation: role of the macrophage. Front Immunol. 2012;3:118.

4. McGuire MK, Nunn ME. Prognosis versus actual outcome. IV. The effectiveness of clinical parameters and tooth survival. J Periodontol. 199970(1):49-56.

5. Parhill JM, Henning BJ, Chapple IL, Heasman PA, Taylor JJ. Association of interleukin-1 gene polymophism with early-onset periodontitis. J Clin Periodontol. 2000;27(9):682-9.

## A37 Challenges in diabetic macular edema

### Tatjana Josifova (tatjana.josifova@augenzentrum-fankhauser.ch)

#### Augenzentrum Fankhauser, Bern, Switzerland

**Keywords:** Diabetic macular edema, Anti-VEGF drugs, Retinal vein pressure, Visual test self–control, Screening

Diabetic macular edema (DME) is one of the most common DR complications. The incidence of DME is in approximately 50 % of the diabetic subjects. In mild form of diabetes, it is present in 3 %, while in moderate NPDR rises to 38 %. In eyes with PDR the incidence of DME reaches 71 % [1].The mechanism of action is in direct connection with chronic retinal microvascular damage. This causes an elevation of intraocular levels of vascular endothelial growth factor A (VEGF), a potent, endothelial-specific mitogen. The production of VEGF A leads to increased permeability of the vasculature and developing of edema [2]. Through history, the standard of care for DME has been laser photocoagulation, which has shown to be effective in stabilizing of vision and reducing the rate of further vision loss by 50 %. Macular laser leads to vision recovery in only 15 % of treated patients [3]. Ocular pharmacotherapy includes the intravitreal drug applications, such as corticosteroids and Anti-VEGF drugs. Among different side effects, one of the greatest problems is the repeated application of the drugs [4].

Medical care plays a crucial role in the prevention of diabetic eye complications. Lower levels of glycosylated hemoglobin (HbA1C) are associated with lower incidence of DME. Study results: An increase of 1 % of HbA1C was associated with a 22 % increase in the 21-year cumulative incidence of DME [1]. Severe hypoglycemic episodes lead to development of DME. A speeded up improvement of the glycemic control lead to rapid worsening of the DME. Patients with DR and DME have significant increase of retinal vein pressure compared to normal subjects [5].

Despite continued improvement in the diagnostic techniques, DR remains the leading cause of blindness in working-age populations. According to the life style and the life expectance, we have to change the old screening or treatment diabetic programs. Genetic investigations should be the first level of the screening goal. Among the risk population, an interdisciplinary approach should have a mandatory role in prevention of diabetic eye complications. Visual test self-control and distribution to an eye-reading center should be an important part in detection of the early eye changes in diabetic patients.

**References**

1. Klein R, Knudtson MD, Lee KE, Gangnon R, Klein BE. The Wisconsin Epidemiological Study of Diabetic Retinopathy. The 25-year incidence of macular edema in persons with type 1 diabetes. Ophthalmology 2009;116(3):497-503.

2. Gao G, Li Y, Zhang, D Gee S, Crosson C, Ma J. Unbalanced expression of VEGF and PEDF in ischemia-induced neovascularisation. FEBS Lett. 2001;489(2-3):270-6.

3. Diabetic Retinopathy Clinical Research Network. A randomised trial comparing intravitreal triamcinolone acetonide and focal/grid photocoagulation for diabetic macular edema. Opthalmology. 2008;115(9):1447-9.

4. Kuppermann BD, Blumenkranz MS, Haller JA, Williams GA, Weinberg DV, Chou C, et al. Randomized controlled study of an intravitreous dexamethasone delivery system in patients with persistent macular edema. Arch Ophthalmol. 2007;125:309-17.

5. Cybulska-Heinrich AK, Baertschi M, Loesche CC, Schoetzau A, Konieczka K, Josifova T, et al. Patients with diabetic retinopathy have high retinal veinous pressure. EPMA J. 2015;6:5

## A38 Overview of the EPMA strategies in laboratory medicine relevant for PPPM

### Marko Kapalla, Juraj Kubáň, Olga Golubnitschaja, Vincenzo Costigliola

#### European Association for Predictive, Preventive and Personalised Medicine (EPMA), Brussels, Belgium

##### **Correspondence:** Marko Kapalla (marko.kapalla@gmail.com) - European Association for Predictive, Preventive and Personalised Medicine (EPMA), Brussels, Belgium

**Keywords:** PPPM, Laboratory medicine, Strategies, Cooperation, Healthcare

Among the cornerstones of PPPM there is laboratory medicine (LM). EPMA finds it essential that all relevant expert organizations contribute with their expertise and their complementary visions and ideas to PPPM and help to change the paradigm from the current reactive medicine to the active PPPM with predictive diagnostics, targeted prevention, personalized treatment and consequently create new, perspective and desired healthcare system which would be the long-term alternative and complement to the current system of disease-oriented “disease care”.

The following actions will be taken into consideration for further cooperation and development of EPMA strategies in LM, relevant for PPPM and advancing healthcare:

1. Systematic classification of all types of predictive markers
2. Establishing new requirements for data and knowledge processing in clinical laboratory information systems from the PPPM perspective
3. Further extension of LM which should include analyses of the external-factor-based materials influencing health
4. Cooperation on the EPMA envisioned PPPM center
5. Supporting necessary cooperation between PPPM, biobanking, bioinformatics and LM
6. Common education of experts in PPPM and LM
7. Cooperation in grant schemes in order to acquire financial resources for further development of technologies and interdisciplinary research in PPPM and LM
8. Publication activities promoting PPPM and LM
9. Common meetings and roundtable discussions dedicated to complex interdisciplinary cooperation and application of the achievements into practical procedures in medicine and healthcare
10. Cooperation in fund raising and promotion of both PPPM and LM
11. Research cooperation in PPPM and LM
12. Political activities related to the role of PPPM and LM in advancing healthcare

The overall cooperation between all professional medical organizations and fields is not only mutually beneficial but also essential. EPMA considers it to be the duty of all medical experts and scientists to use available expertise and knowledge to create vital conditions for an inevitable change in the way people care about their own health.

## A39 EPMA initiative for effective organization of medical travel: European concepts and criteria

### Vincenzo Costigliola, Marko Kapalla, Juraj Kubáň, Olga Golubnitschaja

#### European Association for Predictive, Preventive and Personalised Medicine (EPMA), Brussels, Belgium

##### **Correspondence:** Vincenzo Costigliola (vincenzo@emanet.org) – European Association for Predictive, Preventive and Personalised Medicine (EPMA), Brussels, Belgium

**Keywords:** Medical travel, EPMA, European concepts, Criteria, Predictive preventive personalised medicine (PPPM), Healthcare, Individualised patient profiles, Medical services, Education, Economy

Medical travel (MT) is defined as a package of services provided to the person (healthy or diseased one) that is health/life important, however, not available near his/her home. EPMA considers PPPM concepts as being the most optimal in effective organisation of MT, namely individualised deep diagnostics, targeted preventive measures and treatments tailored to the person. Service packages of advanced MT are distinguishable in five categories:

1. Primary MT: specialised medical resources and treatments (e.g. highly specialised transplants)

2. Revalidation (e.g. neurological revalidation after an accident)

3. Wellness (integrated medical servicers, health supporting care, prediction, prevention and treatment of individual predisposition to pathologies)

4. Spa (specialisation in certain diseases/predispositions, such as arthritic or liver diseases)

5. Home care for older people (possibility of trans-mobility within and outside the Europe).

European concepts for effectively organised MT foresee strong professional networking and clustering of specialised medical centres which fulfil the strict criteria of excellence that are conform to the PPPM concepts and principles presented in the EPMA White Paper created by the leading specialists from over 40 countries [1].

As reported earlier EPMA has nominated a working group which elaborates the criteria for centres of excellence in PPPM [2].

Criteria to be considered in individual countries for participation in the EPMA-MT network are political stability, strong scientific contribution to PPPM concepts and implementation, unique medical expertise contributing to the above pointed categories 1-5, developed infrastructure, good accessibility via means of transportation, well established network of medical facilities offering high quality of medical services to reasonable costs, high standard hotels, favourable ecological situation (preserved nature with tourist paths in the area, fresh water resources, food quality control, etc.), opportunities for a wide range of sports and cultural activities, customer friendly attitude to visitors, and opportunities for investments in this type of medical services.

European countries and international organisations are invited to follow the EPMA initiative for an optimal organisation of MT within and outside the Europe. A series of meetings dedicated to the European concepts and professional networking in MT is organised by the EPMA such as the specialised workshop on MT, “EPMA World Congress 2015”, Bonn, Germany; “European MT”, specialised workshop at the European Parliament, Brussels, Belgium, March 2016, etc.

**References**

1. Golubnitschaja O, Costigliola V, EPMA (2012) General report & recommendations in predictive, preventive and personalised medicine 2012: White Paper of the European Association for Predictive, Preventive and Personalised Medicine. EPMA J. 1(3):14.

2. Golubnitschaja O, Costigliola V, EPMA (2015) EPMA summit 2014 under the auspices of the presidency of Italy in the EU: professional statements. EPMA J 6(1):4.

## A40 Design and innovation in e-textiles: implications for PPPM

### Anthony Kent, Tom Fisher (tom.fisher@ntu.ac.uk), Tilak Dias (tilak.dias@ntu.ac.uk)

#### School of Art and Design, Nottingham Trent University, Nottingham, UK

##### **Correspondence:** Anthony Kent (anthony.kent@ntu.ac.uk) – School of Art and Design, Nottingham Trent University, Nottingham, UK

**Keywords:** Personalisation, Design, Innovation, e-textiles

The aim of this contribution is to summarise an interdisciplinary approach to explain personalisation through design. It examines the design processes in the innovation of e-textiles and subsequently proposes a framework, which exposes the relationships between the actors in innovation, the design process and ‘design thinking’ and their application to personalised medicine. It focuses on a significant and complex new area of e-textiles that ‘host’ ICT by integrating electronic components. It evaluates the recent history of innovation in e-textile technology in the contexts of design practice as an innovation ecosystem. When focused on e-textiles, an interdisciplinary approach offers insights that can both delineate design’s role in innovation, and point towards specific innovations. Efforts to define the relationships between design and other actors in the ‘ecological’ networks that bring about innovation are likely to increase understanding of both the potential of design, and productive ways to encourage such networks. As this research originates in both design practice and academic design research it is well placed to demonstrate the relationships that make up such networks. The review concludes that e-textiles can make a valuable contribution to PPPM by mediating information through smart clothing and accessories. Both the design of e-textile objects and design of their place in the PPPM ecosystem will contribute to more effective personalised medicine.

## A41 Biobank in Pilsen as a member of national node BBMRI_CZ

### Judita Kinkorová, Ondřej Topolčan (TOPOLCAN@fnplzen.cz)

#### Faculty Hospital in Pilsen, Charles University Medical Faculty in Pilsen, Pilsen, Czech Republic

##### **Correspondence:** Judita Kinkorová (KINKOROVAJ@fnplzen.cz) - Faculty Hospital in Pilsen, Charles University Medical Faculty in Pilsen, Pilsen, Czech Republic

**Keywords:** Biobank, BBMRI, Research infrastructure, International cooperation

Biobanks and Biomolecular Resources Research Infrastructure - European Research Infrastructure Consortium (BBMRI-ERIC) is one of the largest European infrastructures, consortium consisting of 54 members with more than 225 associated organizations, mostly biobanks from over 30 countries in Europe. BBMRI-CZ was established under the governance of the Ministry of Education and became a founding member of the European biobanking infrastructure BBMRI-ERIC. The Czech consortium is coordinated by Masaryk Memorial Cancer Institute in Brno and the following members are: Charles University (1^st^ Medical Faculty in Prague, Medical Faculty in Hradec Králové, and Medical Faculty in Pilsen), Palacky Universtity Olomouc (Medical Faculty).

The main purpose of biobank in Pilsen is to establish a platform for the research and for national and international cooperation. Main topics for research at the Faculty Hospital in Pilsen and at the Medical Faculty – Charles University in Pilsen are related to the use of biomarkers in oncology, particularly for: prevention – active search for risk factors, early diagnostics, therapy effect monitoring, prognosis estimation, and recurrence disease and disease progression diagnostics.

Main problems to be solved now are: definition of the character and purpose of the biobank, choice of individuals and frequency of the monitoring, pre-analytic problems (solved), data quantity and quality, unified system of the storage, ethical issues solution on the national level that has been currently missing.

We assume that multidisciplinary team of specialists, functioning infrastructure and harmonizing all necessary processes enable that the biobank will not serve only for the local hospital purpose, but it will play an important role for the national and international cooperation.

## A42 Big data in personalized medicine: hype and hope

### Matthias Kohl (Matthias.Kohl@hs-furtwangen.de)

#### Department of Medical and Life Sciences, Furtwangen University, Villingen-Schwenningen, Germany

**Keywords:** Big data, Data standards, Data integration, Data analysis, Statistical machine learning, Patient-specific learning methods

Today huge amounts of data are generated by the omics disciplines and clinical data are collected in patient data management systems for more than 20 years now. There is the hope that big data will lead to a more effective and more personalized medicine. But how and how quickly will big data transform healthcare?

I will elaborate on the following key challenges of big data: standards, integration, and statistical analysis where I will focus on biomedical data. Many groups of scientists have been working on establishing the relevant data standards for omics data. They include standards for experiment description, data exchange, terminology, and experiment execution. I will introduce MIAME (Minimum Information About a Microarray Experiment) [1] and some analogs for other omics data.

An important goal of data standardization is the simplification of the subsequent data integration [2], i.e., the combination of data of different sources to provide a unified view on these data. I will briefly summarize some current approaches in personalized medicine such as The Cancer Genome Atlas Project (TGCA) [3]. In the last part, I will talk about the statistical analysis of big data [4]. Here I will sketch the workflow of building predictive models by applying modern statistical machine learning methods. In particular, I will present PATIENTS one of my projects that aims at the development of patient-specific learning methods [5].

**References**

1. Brazma A, Hingamp P, Quackenbush J, Sherlock G, Spellman P, Stoeckert C, et al. Minimum information about a microarray experiment (MIAME)-toward standards for microarray data. Nat Genet. 2001;29(4):365-71.

2. Chervitz SA, Deutsch EW, Field D, Parkinson H, Quackenbush J, Rocca-Serra P, et al. Data Standards for Omics Data: The Basis of Data Sharing and Reuse. Methods Mol Biol. 2011;719:31–69.

3. Cancer Genome Atlas Research Network, Weinstein JN, Collisson EA, Mills GB, Shaw KR, Ozenberger BA, et al. The Cancer Genome Atlas Pan-Cancer analysis project. Nat Genet. 2013;45(10):1113-20.

4. Schneeweis S. Learning from big health care data. N Engl J Med. 2014;370(23):2161-3.

5. Visweswaran S, Angus DC, Hsieh M, Weissfeld L, Yealy D, Cooper GF. Learning patient-specific predictive models from clinical data. J Biomed Inform. 2010;43(5):669-85.

## A43 The 3P approach as the platform of the European Dentistry Department (DPPPD)

### Anatoly A. Kunin, Natalia S. Moiseeva

#### Voronezh N.N. Burdenko State Medical University, Voronezh, Russia

##### **Correspondence:** Natalia S. Moiseeva (natazarova@yandex.ru) – Voronezh N.N. Burdenko State Medical University, Voronezh, Russia

**Keywords:** 3P approach, Preventive personalized dentistry, European Dentistry Department

Many strategic and tactical issues are to be resolved for implementation of predictive and preventive measures for various dental diseases. On September 13-15, 2011, in Bonn, Germany, the Board of the European Association for Predictive, Preventive and Personalised Medicine (EPMA, Brussels, Belgium) made a decision to establish the European Department of Dentistry (DPPPD). The task was assigned to the Head of the Therapeutic Dentistry Department of Voronezh State Medical University, the Director of the Dental Institute, Dr. Med. Sc., Prof. Anatoly A. Kunin. The Department was officially opened at the International Dental Congress held on February 24, 2012, in Voronezh, Russia, in the presence of the EPMA Secretary-General Prof. Olga Golubnitschaja and 32 professors from 8 countries participating in the roundtable discussions. Over the three-year period of its existence, the EPMA European Department of Dentistry has carried out certain works on promoting prediction and prevention of dental diseases in the DPPPD member countries. Italy, The United States, Estonia and Russia have taken the most active part in this direction. The work resulted in organization and coordination of the Dental Section at the EPMA World Congress held on September 19-21, 2013, in Brussels, Belgium. Scientific reports and their discussion under the program made a certain contribution to the development of diagnostic predictive and preventive techniques in dentistry. Some of them should be distinguished: preclinical diagnostics of dental caries (light-induced fluorescence and electrical conductivity of dental hard tissues; cytological and bacterioscopical indicators of initial oral manifestations; normal and pathological values; different options of dental hard tissues micro-damage in dental caries treatment; high-tech methods for caries and periodontal diseases prevention; numerous studies of consumable items for dental caries prevention, and many other things.

Special emphasis should be placed on the strategically important EPMA activities based on the consolidation of 45 participating countries and their vigorous interdisciplinary activity focused on Future Medicine where the 3P (predictive, preventive and personalized) approach based on up-dated biomedical techniques and developments is a priority. That is the reason why the Innovative Centers established on the basis of the European Department of Dentistry provide high-tech medical services with due consideration of a personalized approach for patients.

## A44 The endometrium cytokine patterns for predictive diagnosis of proliferation severity and cancer prevention

### Andrii I. Kurchenko^1^, Vasyl A. Beniuk (benyuk@i.ua)^1^, Vadym M. Goncharenko (dr.v.goncharenko@gmail.com)^1,2^, Rostyslav V. Bubnov^2,3^, Nadiya V. Boyko (nadiya.boyko@cassovialifesciences.eu)^3,4^, Andriy M. Strokan (strokandr@mail.ru)^2^

#### ^1^Bogomolets National Medical University, Kyiv 01601, Ukraine; ^2^Clinical Hospital ‘Pheophania’ of State Affairs Department, Zabolotny str., 21, Kyiv 03680, Ukraine; ^3^Zabolotny Institute of Microbiology and Virology, National Academy of Sciences of Ukraine, Zabolotny Str., 154, Kyiv 03680, Ukraine; ^4^Cassovia Life Science, Komenského 1337, 02401 Kysucké Nové Mesto, Slovakia

##### **Correspondence:** Rostyslav V. Bubnov (rostbubnov@gmail.com) – Zabolotny Institute of Microbiology and Virology, National Academy of Sciences of Ukraine, Zabolotny Str., 154, Kyiv 03680, Ukraine

**Keywords:** Predictive preventive personalized medicine, Endometrium hyperplasia, Receptor biology, Ultrasound, Hysterescopy, Cytokine patterns, Pro-inflammatory cytokines

**Introduction:** Endometrial hyperplasia (EH) holds leading position in the structure of gynecological pathology, evoked by changing women lifestyles, widespread abortion, collateral diseases and limitations of conservative treatment due to the contraindications to hormone therapy. Endocrine factor is crucial [1-3], however not the only a part of EH pathogenesis, hormonal reception disorders, are related to immune responses and apoptosis, etc. Therefore, identification of new pathogenic mechanisms of EH, including immunological, development on results of these studies of new therapeutic and diagnostic approaches will improve outcome and timing of treatment, quality of women life.

The aim was to study the levels of cytokines in flushing from the uterus in women with endometrial pathology.

**Materials and Methods:** We included to the study 192 women who passed the survey and were treated at the General Gynecology Center of Clinical Hospital “Pheophania”. Age of women ranged from 22 to 83 years and averaged 46,0 ± 8,3 years; in reproductive period there were 54 (34.6 %) patients, 49 in premenopausal (31.4 %) and 53 in postmenopausal (34.0 %) cases. The control group (n = 36), was formed by three sub-groups, which included 12 (33.3 %) women of each age period. Patients were divided into 7 groups: the first group - patients with simple EH (n = 28 (17.9 %), the second group consisted of patients with simple EH with atypia (n = 24 (15.4 %), the third group represented by patients with complex EH without atypia (n = 24 (15.4 %),25 (16.0 %) women with complex (adenomatous) endometrial hyperplasia with atypia made up the fourth group, the fifth group - 31 (19.9 %) observations - women with endometrial polyposis (EP) and sixth group - 24 (15.4 %) women with endometrial cancer (adenocarcinoma). The sixth group included patients with first stage of endometrial cancer. The seventh control group consisted of 36 healthy examined women.

All the patients underwent general clinical examination, indications for hospitalization were the results of transvaginal ultrasonography (ultrasound signs of EH). All patients underwent hysteroscopy with further diagnostic scraping the walls of the uterus and cervical canal. Determination of the concentrations of cytokines IL-1, IL-2, IL-6 and TNF performed solid enzyme-linked immunosorbent assay by means of commercial reagent kits produced by LTD "Cytokines" (St. Petersburg, Russia).

**Results:** Simple non-atypical endometrial hyperplasia accompanied by an increase in flushing from the uterus of TNF level, IL-1 β level, IL-6 and IL-2 by 2 times.

The presence of complex non-atypical endometrial hyperplasia accompanied by an increase in flushing from the uterus of TNF levels by 1.5 times, increase levels of IL-1β in 3-4 times, higher levels of IL-6 by 2 times and IL-2 by 1.5 times.

Pro-inflammatory cytokines level in flushing from the uterus in women with atypical forms were sharply increased. Thus, the level of TNF exceeded the reference values by 5-6 times the level of IL-1β in a simple EH with atypia – by 5 times, with complex EH with atypia - 10-11 times. The content of IL-6 in flushing from the uterus in patients with simple EH with atypia was increased by 6 times, with complex EH with atypia – by 10 times. The level of IL-2 increased in these groups by 2.5 times.

Endometrial polyposis accompanied by increased pro-inflammatory cytokines (from 2 to 6 times), but is characterized by wide fluctuations in values, in our opinion, due to the presence of combined chronic process in the uterus.

In malignant transformation of the endometrium there is a significant increase in local inflammatory cytokines in the uterus, which characterizes the maximum activation of immune defense mechanisms, and appears increasing of TNF and IL-6 in 11-12 times, IL-1β in 25 times and IL-2 in 2.5 times.

**Conclusion:** The development of endometrial proliferative processes is accompanied by a local increase in the levels of pro-inflammatory cytokines, correlating with degree of pathological transformation of the endometrium.

**Outlook and Expert recommendations:** Defining the level of pro-inflammatory cytokines in the uterus may be used as diagnostic determinants in defining the nature of intrauterine pathology and criterion of efficiency of conservative therapy phase.

There should be a sufficient evidence study to determine relationships in endometrial receptor system, genetics, immune pathways and vascular patterns to complement the diagnostic algorithm that will allow the development of novel treatments and model-guided approach.

Vaginal / uterus microbiome studies are necessary in the various stages of disease and in different at-risk populations, in regards to the role of host genotype, involvement hormonal receptors might suggest promising approach for understanding pathogenesis of chronic gender-related inflammatory diseases, development personalized treatments, diet and lifestyle corrections [4].

**References**

1. Goncharenko VM, Beniuk VA, Kalenska OV, Demchenko OM, Spivak MY, Bubnov RV. Predictive diagnosis of endometrial hyperplasia and personalized therapeutic strategy in women of fertile age. EPMA J. 2013;4:24.

2. Goncharenko VM, Beniuk VA, Vyniarskyi YM, Bashynskyi SM, Bubnov RV. Assessment of endometrial receptor systems for PPPM approach for endometrial hyperplasia in reproductive age women. EPMA J. 2014;5(Suppl 1), A39. doi:10.1186/1878-5085-5-S1-A39

3. Goncharenko VM, Beniuk VA, Vyniarskyi YM, Bashynskyi SM, Bubnov RV. Personalized treatment strategy for atypical endometrial hyperplasia with regards to age, comorbidities and endometrial receptor status. EPMA J. 2014;5(Suppl 1):A40. doi:10.1186/1878-5085-5-S1-A40.

4. Bubnov RV, Spivak MY, Lazarenko LM, Bomba A, Boyko NV. Probiotics and immunity: provisional role for personalized diets and disease prevention. EPMA J. 2015;6:14.

## A45 A monocyte-based in-vitro system for testing individual responses to the implanted material: future for personalized implant construction

### Julia Kzhyshkowska^1,2,3^, Alexandru Gudima^1^, Ksenia S. Stankevich^1,4^, Victor D. Filimonov^4^, Harald Klüter^1,2^, Evgeniya M. Mamontova^4^, Sergei I. Tverdokhlebov^4^

#### ^1^Institute of Transfusion Medicine and Immunology, Medical Faculty Mannheim, Heidelberg University, Mannheim, Germany; ^2^German Red Cross Blood Service Baden-Württemberg – Hessen, Mannheim, Germany; ^3^Laboratory for translational cellular and molecular biomedicine, Tomsk State University, Tomsk, Russia; ^4^National Research Tomsk Polytechnic University, Tomsk, Russia

##### **Correspondence:** Julia Kzhyshkowska (julia.kzhyshkowska@googlemail.com) – Institute of Transfusion Medicine and Immunology, Medical Faculty Mannheim, Heidelberg University, Mannheim, Germany

**Keywords:** Macrophage, Inflammatory reaction, Implant construction, Personalization

Macrophages are the cells responsible for the initiation of the foreign body response and therefore regulate the immune reaction to implant materials. Adverse reactions of macrophages result in the individual profile of inflammatory complications caused by titanium implants, therefore innovative coating materials are under development in the leading laboratories in the world. A promising biomaterial is Polylactic acid (PLA) which provides high biocompatibility, processability and good mechanical properties. PLA is a synthetic biodegradable polymer used in manufacturing of implants and coatings such as resorbable sutures, clips, plates and screws and in drug delivery devices. However, degradation of PLA in the body into lactic acid can cause chronic inflammation and implant intolerance. To address this issue, surface modifications of PLA-based biomaterials may be used. Macrophages regulate the inflammatory reaction to implants, therefore their response to unmodified and modified PLA-based materials was analyzed. We found that human primary monocyte-derived macrophages react in a donor-specific way to PLA samples modified with Brilliant Green dye (BG1, BG2, BG3) and that these modifications had a stimulatory effect on the production of TNFα and CCL18 released by both M1 and M2. Additionally we studied the expression of macrophage mannose receptor CD206 and stabilin-1 to analyze whether modified or unmodified PLA samples can induce an M2 tolerogenic phenotype in monocytes. We found that unmodified PLA samples increase the expression of both markers, while the modified PLA modulate their expression in a donor specific way. Our results show that the inflammatory responses of macrophages can be changed by modification of PLA material surface. A monocyte-based in-vitro system for testing individual responses to the implanted material was established for selecting personalized implant variant.

**Funding:** IMMODGEL project, Grant No (602694).

## A46 Prediction and prevention of adverse health effects by meteorological factors: Biomarker patterns and creation of a device for self-monitoring and integrated care

### Ulyana B. Lushchyk^1^, Viktor V. Novytskyy (v.novytskyy@gmail.com)^2^, Igor P. Babii (chiefdoctor@istyna.kiev.ua)^3^, Nadiya G. Lushchyk (umi2012T@gmail.com)^4^, Lyudmyla S. Riabets (l.riabets@itmed.com.ua)^5^, Ivanna I. Legka (IvannaLegka2015@gmail.com)^6^

#### ^1^Istyna-Veritas Research Center, Kyiv, Ukraine; ^2^Institute of Mathematics of NAS of Ukraine, Kyiv, Ukraine; ^3^Clinic of Healthy Vessels, Kyiv, Ukraine; ^4^Medical center “Ukrainian medical innovations”, Ternopil, Ukraine; ^5^Veritas IT Med Center for Innovative Medical Technologies, 4 Williams Str., Kyiv 03191, Ukraine; ^6^Specialized clinic “Vessels, brain, neurorehabilitation”, Muscat, Oman

##### **Correspondence:** Ulyana B. Lushchyk (u.lushchyk@gmail.com) – Istyna-Veritas Research Center, Kyiv, Ukraine

**Keywords:** Medical travel, Angiotherapy, Meteo-dependence, Vascular screening

**Objectives:** Statistics shows that populations around the world are highly sensitive towards meteorological factors. Vascular disorders result in a systemic imbalance, further, impairing adaptation to sudden meteorological changes.

The research objectives are to find new personalized algorithms to predict adverse health effects by meteorological factors and create targeted preventive measures [1-3].

**Technological approaches:** Technological approach is focused on establishing specific biomarker patterns reflecting individual reactions towards changing meteo-factors such as atmospheric pressure, air humidity, air temperature, wind velocity and direction, magnetic vibrations.

**Results interpretation and Outlook:** The results comprise the proto-type of a portable meteo-station for self-monitoring and integrated care. The developed device is applicable to several patient cohorts such as those suffering from cardio-vascular and psychoneurological disorders.

**References**

1. Lushchyk UB, Novytskyy VV, Babii IP, Lushchyk NG, Babyuk OV, Kovpak AO, et al. Monitoring brain functioning with evident based IT-technologies with feedback control. EPMA J. 2014;5(Suppl 1):A100.

2. Lushchyk UB, Novytskyy VV, Babii IP, Riabets LS, Kolomiychuk OP, Legka II. Screening of vascular pathologies – evidence-based monitoring of prevention efficiency. EPMA J. 2014;5(Suppl 1):A91.

3. Lushchyk UB, Novytskyy VV, Bubnov RV, Legka II, Kovpak AO. IT- technology for vascular monitoring as an evidential base in prevention and prediction of a vascular pathology. EPMA J. 2014;5(Suppl 1):A64.

## A47 Targeting "disease signatures" towards personalized healthcare

### Mira Marcus-Kalish, Alexis Mitelpunkt, Tal Galili, Neta Shachar, Yoav Benjamini

#### Department of Statistics and Operations Research, the Sackler Faculty of Exact Sciences, the Sagol School of Neurosciences, Tel Aviv University, Tel Aviv, Israel

##### **Correspondence:** Mira Marcus-Kalish (miram@post.tau.ac.il) – Department of Statistics and Operations Research, the Sackler Faculty of Exact Sciences, the Sagol School of Neurosciences, Tel Aviv University, Tel Aviv, Israel

**Keywords:** Disease signature, 3C strategy, Categorization, Clustering, Classification, ADNI database.

The goal to provide individualized tailored treatment to patients in terms of efficacy and adverse events depends on the ability to define the "disease signatures". A term that became a premise in the therapeutic targets, precision medicine and disease prediction processes, but depends heavily on the comprising, analysis and translation of all relevant personal data. It may include all the micro- and macro-factors of the human body functioning in its physical, mental and community environment, such as age, gender, clinical tests, biological markers, lifestyle, medical history, genetics, imaging etc. The new sophisticated and advanced technologies are enabling the capturing of these parameters through profound insights on the human body functioning, in various levels and degrees of sensitivity, providing "Big Data" initiated from various sources. The goal is to analyze simultaneously all these identified and captured factors, including their interplay, translated to "disease signatures" towards responsible personalized, preventive and predictive medicine applied in the clinics.

Thus, it creates the need to bridge barriers between "Big Data" and "Small Data" such as the simultaneous analysis of different types of data, sometimes related to a few number of people (many parameters, few records) tackling over fitting as well as database leakage [1], replicability [2] and reproducibility barriers. For example, hospital databases with limited availability of healthy records and different diagnostic tests undertaken by healthy and sick people, suffer from leakage and replicability barriers.

Furthermore, other challenges are:

- Expert knowledge is valuable but current diagnosis might be misleading.

- Compensatory mechanisms, obscuring the linkage between biological markers (i.e. imaging, pathology and genetics) and disease manifestation, are difficult to discover.

- Big Data: Big potential but increased chances of capturing irrelevant markers.

Thus to address these challenges new technologies and targeted tools are being developed, among them the "3C" strategy developed in our group. The goal is to meet the growing sensitivity and specificity needs in representing the medical data variance and its relevance and contribution to personalized medicine.

**The 3C- Categorization, Classification & Clustering- strategy**, developed as part of the Medical Informatics sub-project in the Human Brain Project Flagship, is targeting to utilize and converge the medical expert knowledge, the disease manifestations and the potential biomarkers towards personalized prediction and treatment.

The methodology includes the three steps: Categorization, Clustering and Classification, based on supervised and unsupervised algorithms.

Step 1 - Categorization of variables into three types: (1) disease diagnosis as assigned in the electronic health record (EHR), by the medical expert. (2) Clinical measurements reflecting the patient’s condition and functionality. (3) Potential biological markers, (Proteins, imaging, etc.) proposing predictive value for disease risk, deterioration or for severity. Step 2 - Feature selection and Clustering. Following the categorization and feature selection of the clinical measurements, an unsupervised learning creates sub-classes representing new disease diagnosis classes based on the differences in the manifestation of the disease. Step 3 - Classification including potential biomarkers. Given the homogenous clusters and following a feature selection of the potential biomarkers, this step is seeking for relations between identified potential biomarkers and each of the homogenous clusters using hierarchical decision trees, or other rule based analysis.

A preliminary feasibility study of the "3C strategy" was applied successfully to the Alzheimer's disease Neuroimaging Initiative (ADNI) cohort, identifying and suggesting 10 sub-classes, rather than the 5 assigned in the ADNI data set – AD (Alzheimer disease), EMCI, LMCI (Early & Late Mild Cognitive Impairment), SMC (Significant Memory Concern) and CN (Normal).

**References**

1. Brown HW, Wexner SD, Segall MM, Brezoczky KL, Lukacz ES. Accidental bowel leakage in the mature women's health study: prevalence and predictors. Int J Clin Pract. 2012;66(11):1101-8.

2. Rosenblatt JD, Vink M, Benjamini Y. Revisiting multi-subject random effects in fMRI: advocating prevalence estimation. Neuroimage. 2014;84:113-21.

## A48 Influence of the skin imperfection on the personal quality of life and possible tools for objective diagnosis

### Agnieszka Migasiewicz^1^, Markus Pelleter^2^, Joanna Bauer^3^, Ewelina Dereń^3^, Halina Podbielska^3,1^

#### ^1^Wroclaw University School of Physical Education, Faculty of Physiotherapy, Wroclaw, Poland; ^2^VIANESSE AG, Research and Development, Engelberg, Switzerland; ^3^Wroclaw University of Technology, Department of Biomedical Engineering, Wroclaw, Poland

##### **Correspondence:** Halina Podbielska (info@halinapodbielska.pl) – Wroclaw University of Technology, Department of Biomedical Engineering, Wroclaw, Poland

**Keywords:** Skin imperfection, Cellulite, Quality of life, Thermovision, Thermographic analysis

**Scientific objectives:** The personal attitude to own body is undoubtedly correlated with the quality of life (QoL). Our recent study on the influence of the lipodystrophy (cellulite) on the quality of life of women showed that their quality of life is influenced by the skin imperfection depending on the cellulite stage [1]. In the study group, lipodystrophy according to the Nürnberger-Müller scale was diagnosed in 60 subjects. Te Polish version of The Brief Illness Perception Questionnaire – B-IPQ was used for evaluation of the QoL [2, 3]. Currently, there is no reliable objective diagnosis of this kind of lipodystrophy. In this paper, we propose to exploit the thermal imaging for cellulite diagnosis. The superficial body temperature distribution is influenced by many factors, among them the fat cells layer play an important role.

**Technological approaches:** Our study is devoted to the validation of the use of infrared thermal imaging for the assessment of cellulite and to development of the objective method to evaluate the severity of cellulite. Thermal images of the thigh were recorded and further processed to quantify the temperature distribution. 12 young women (20-24 years old) with various degrees of cellulite were enrolled in to the study. We developed our own application based on Java Technology, for importing and analyzing data from infra-red images. Our new software (ThermaAnalyzer 2.0) is designed for assessment of the degree of cellulite. It is based on an algorithm, which can search for irregularities (blemishes) on thermal image. By analyzing the temperature distribution of the examined part of body, it is possible to diagnose the cellulite stage and thus, the influence of possible anti-cellulite therapy.

**Results interpretation:** Our observations revealed that people with higher degree of cellulite have more irregular superficial temperature distribution seen as the spots on the thermal image. Thermal imaging can be an objective method for lipodystrophy diagnosis and monitoring of therapy.

**Outlook and Expert recommendations:** Further studies in larger group are required to elaborate the reliable diagnosis based on thermal imaging. This can help to personalize the treatment, which include the proper diet, cosmetics, physiotherapeutic agents and physical activities.

**References**

1. Migasiewicz A, Dereń E, Podbielska H, Bauer J, Women’s quality of life depending on the cellulite stage (in Polish), Acta Bio-Optica et Informatica Medica – Inzynieria Biomedyczna, 2014;20(4):217–26. http://www.inzynieria-biomedyczna.com/index.php/archiwum/formacie-pdf/2014-4/567-v20n4a4migasiewicz217-226/download

2. Broadbent E, Petrie KJ, Main J, Weinman J. The Brief Illness Perception Questionnaire (BIPQ). J Psych Res. 2006;60:631–7.

3. Kwestionariusz Percepcji choroby - wersja skrócona, The Illness Perception Questionnaire Polish version, http://www.uib.no/ipq/pdf/B-IPQ-Polish.pdf

## A49 The new direction in caries prevention based on the ultrastructure of dental hard tissues and filling materials

### Natalia S. Moiseeva, Anatoly A. Kunin, Dmitry A. Kunin

#### Voronezh N.N. Burdenko State Medical University, Voronezh, Russia

##### **Correspondence:** Natalia S. Moiseeva (natazarova@yandex.ru) – Voronezh N.N. Burdenko State Medical University, Voronezh, Russia

**Keywords:** Caries prevention, Tooth ultrastructure, Physical effects

A high prevalence of secondary caries as well as difficulties in verification of its stages set the problem of search for new approaches to early diagnostics, prevention and treatment of the carious process.

Nowadays the development of high-tech methods of assessment and treatment in dentistry raise requirements to the core elements of White Esthetics, such as color, form, function, and material durability. Numerous studies have shown that smaller particles of filling material makes the esthetics better and facilitate gingival attachment and adhesion that prevents from tooth-filling physical and chemical disbonding and secondary caries occurrence. However, the microfilled composites do not possess high strength properties which prevents from using them in the areas of increased load and in chewing teeth.

The new direction in dentistry presupposes an enlargement of filling material particles with its high esthetic properties reserved wherein there is an increase in durability and elasticity. It is especially important because of frequent cases of cracks and chips in restorations after treatment of teeth that carry increased functional load. A large number of scientific researches made it possible to come to a conclusion that the physical factors of therapy influence on the ultrastructure of filling materials in which there are changes in the substance structure at the micro level, which positively effects on the strength properties of the material. Teeth enamel has "enamel tunnels" and "enamel bridges" which regulate exchange processes between enamel, dentine, oral liquid and filling materials.

It is essential to search for options of physical fields’ action on filling materials, which, without affecting their esthetic properties, will enable to increase their durability and microhardness so that they become more resistant to mechanical and physical effects that will contribute to a longer lifetime of fillings. Thus, the provided data allow making the conclusion about a variety and diversity of changes occurring in a filling and in a tooth in the carious process development, which predicts perspectives for carrying out further research in the field of secondary caries prevention.

## A50 The use of LED radiation in prevention of dental diseases

### Natalia S. Moiseeva, Yury A. Ippolitov, Dmitry A. Kunin, Alexei N. Morozov, Natalia V. Chirkova, Nakhid T. Aliev

#### Voronezh N.N. Burdenko State Medical University, Voronezh, Russia

##### **Correspondence:** Natalia S. Moiseeva (natazarova@yandex.ru) – Voronezh N.N. Burdenko State Medical University, Voronezh, Russia

**Keywords:** Prevention, LED radiation, Tooth enamel

The modern dentistry has a wide range of medicines for caries prevention, but spreading allergization of the population requires the development of alternative methods for dental diseases prediction. Such preventive measures include different types of light radiation. The purpose of our research was studying the LED radiation influence of the ULOХ device on the mineral exchange in tooth enamel.

A method of enamel acid biopsy according to Leontyev and Distel offered the opportunity to determine the speed of acid solubility of enamel calcium. The X-ray spectral microanalysis (RMA) allowed studying the chemical pattern of the enamel surface layer with the accuracy of 0.01 %. The equipment used: scanning electron microscope CamScan S4.

Such factors as tooth extraction and its isolation from the saliva reflected adversely on the level of enamel mineral exchange, which consisted in increasing of acid solubility of enamel calcium by 30.13 % in a day after the tooth extraction, which is 7.66 μmol/min more than the reference value. A reliable reduction of calcium by 20.3 % and phosphorus by 19.7 % was in parallel observed in the enamel surface layer. Even upon completion of the observations (in a month), the results did not revert to the initial values.

We observed other dynamics of mineral exchange in the group of teeth exposed to the LED radiation by the ULOX device: the speed of acid solubility of enamel decreased by 23.11 % in a day after the radiation, and the content of calcium and phosphorus increased by 11.63 % and 8.53 % respectively, which is more than the initial values by 3.19 and 1.01 % by weight respectively. And by the 4th week the studied indicators did not differ much in comparison with the initial level.

The changes in tunnel structures and exchange processes stimulated by light-induced radiation and with the use of ROCS medicines according to our scientific data. Thus, the research data prove that the LED radiation facilitates increase of calcium and phosphorus content in the enamel surface layer and reduction in speed of enamel acid solubility. This indicates the mineral exchange normalization as well as a tooth enamel resistance and micro-hardness increase.

## A51 Status of endothelial progenitor cells in diabetic nephropathy: predictive and preventive potentials

### Mahmood S. Mozaffari, Jun Yao Liu (JYLIU@gru.edu), Babak Baban (BBABAN@gru.edu)

#### Department of Oral Biology, Georgia Regents University, Augusta, Georgia 30912, USA

##### **Correspondence:** Mahmood S. Mozaffari (MMOZAFFA@gru.edu) – Department of Oral Biology, Georgia Regents University, Augusta, Georgia 30912, USA

**Keywords:** Diabetic nephropathy, Stems cells, Blood, Kidney, Predictive and preventive strategies

**Scientific Objective:** The world-wide epidemic of diabetes mellitus, and associated micro- and macro-angiopathic complications, is one of the most pressing health-related challenges of our time. Indeed, diabetic nephropathy is the leading cause of end-stage kidney disease thereby requiring renal replacement therapy. This not only suggests inadequacy of current drug therapies but also failure of endogenous protective and reparative mechanisms. Abnormalities of the endothelium constitute major pathogenic mechanisms contributing to the initiation and progression of diabetic nephropathy. Endothelial progenitor cells (EPCs) are of myeloid origin and capable of replacing injured endothelial cells and differentiating into functional endothelial cells. Thus, we explored the status of EPCs in animal models of diabetes mellitus, in the setting of nephropathy, and also determined whether the diabetic milieu affects the survivability of these cells.

**Technical Approach/Methods:** Streptozotocin-induced type 1 diabetic mice and obese type 2 diabetic db/db mice, and their appropriate controls, were used for this study. Accordingly, indices of glycemic status and kidney function were determined in the context of assessment of the status of EPCs in peripheral blood and/or renal tissue. In addition, we explored whether diabetic animals display greater apoptosis of EPCs.

**Results/Interpretation:** Type 1 diabetic mice displayed significant reduction in EPCs (Sca1^+^Ckit^+^CD31^+^ cells) in the peripheral blood 6 weeks after streptozotocin injection in association with manifestations of diabetic nephropathy (e.g., proteinuria). Since type 2 diabetes mellitus accounts for the majority of diabetic cases, subsequent studies were carried out in db/db mice and their db/m controls. The db/db mice showed significant increases in insulin resistance index and hemoglobin A1c in association with marked increase in albuminuria but reduction in creatinine clearance in association with significant increases in apoptotic and necrotic cell death of kidney cells. The peripheral blood cells of db/db mice displayed reduction in EPCs compared to those of db/m controls. Further, kidney cells prepared from experimental groups also showed reductions in EPCs. Using markers of EPCs and apoptosis we observed increased apoptosis of EPCs in cells prepared from kidneys of db/db compared to those of db/m mice. Collectively, the results suggest that apoptosis of EPCs likely contributes to eventual manifestation of renal failure in diabetes mellitus.

**Outlook/Expert Recommendations:** The observation of a similar pattern of decline in EPCs in the peripheral blood and the kidney suggests that monitoring of blood levels of EPCs may be predictive of failure of their protective and reparative features. Further, strategies aimed at reducing apoptosis of EPCs and “empowering” them through genetic modulations could potentially serve as effective strategies to prevent end-stage renal failure in diabetic patients.

## A52 The status of glucocorticoid-induced leucine zipper protein in salivary gland in Sjögren’s syndrome: predictive and personalized treatment potentials

### Mahmood S. Mozaffari, Jun Yao Liu, Rafik Abdelsayed, Xing-Ming Shi, Babak Baban

#### Department of Oral Biology, College of Dental Medicine, Georgia Regents University, Augusta, Georgia 30912, USA

##### **Correspondence:** Mahmood S. Mozaffari (MMOZAFFA@gru.edu) – Department of Oral Biology, College of Dental Medicine, Georgia Regents University, Augusta, Georgia 30912, USA

**Keywords:** GILZ, Sjögren’s syndrome, Salivary glands, Inflammation, Predictive personalized treatment potentials

**Scientific Objective:** The glucocorticoid-induced leucine zipper (GILZ) protein is a pivotal player in mediating the anti-inflammatory effects of glucocorticoids. However, its status and role in salivary gland inflammation and dysfunction in Sjögren’s Syndrome (SS) is not established. Thus, we tested the hypothesis that SS is associated with reduced GILZ expression and increased leukocyte infiltration thereby contributing to inflammation and cell death in salivary glands.

**Technical Approach/Methods:** We utilized the non-obese diabetic (NOD) mice, a model of SS-like disease, in association with immunostaining and flow cytometry-based studies. These studies were complemented with *in vitro* studies whereby interleukin (IL)-23-treated salivary gland cells were co-cultured with mesenchymal stem cells (MSCs) which over express GILZ (MSCs/GILZ) or express the green fluorescent protein (MSCs/GFP); IL-23 is known to increase generation of pro-inflammatory cytokine, IL-17. In addit ion, we carried out immunostaining of lower lip biopsy samples of human subjects without or with a diagnosis of SS.

**Results/Interpretation:** Salivary glands of NOD mice displayed a) reduced GILZ expression, b) foci of inflammatory cell infiltrates including T and B lymphocytes as well as M1 and M2 macrophages and c) increased pro-inflammatory IL-17 but reduced anti-inflammatory cytokine, IL-10. These changes were accompanied with increased apoptosis and necrosis of salivary gland cells of NOD than control mice. Further, IL-23-treated salivary gland cells displayed marked increase in IL-17 which was reversed with MSCs/GILZ but not MSCs/GFP. Importantly, lower lip biopsy samples of SS patients showed marked reduction of GILZ but increased IL-17 immunostaining compared to those of non-SS subjects. Collectively, the results are suggestive of GILZ playing a role in inflammation and cell death in salivary gland in SS.

**Outlook/Expert Recommendations:** The present study has established the relevance and significance of GILZ as a novel predictive and prognostic molecular fingerprint for SS. Further, assessment of minor salivary gland GILZ expression could potentially be a more sensitive approach to help with diagnosis of SS, at early stage of the disease, than that based on leukocyte infiltration. Importantly, subsequent studies should establish whether treatment with MSCs/GILZ ameliorates signs and symptoms of salivary malfunction of SS for which effective treatment options remain elusive.

## A53 Maximal aerobic capacity - important quality marker of health

### Jaroslav Novák^1^, Milan Štork^2^, Václav Zeman^1^

#### ^1^Institute of Sports Medicine, Medical Faculty of Charles University, Plzeň, Czech Republic; ^2^Faculty of Applied Electronics, Western-Bohemian University, Plzeň, Czech Republic

##### **Correspondence:** Jaroslav Novák (novakj@lfp.cuni.cz) – Institute of Sports Medicine, Medical Faculty of Charles University, Plzeň, Czech Republic

**Keywords:** Maximal aerobic capacity, Maximal performance, Bicycle ergometer, Prediction of VO2max

**Aim:** VO2max represents limiting complex interaction of our pulmonary and cardiovascular systems and oxygen utilization on cellular level. It could be directly measured and/or indirectly predicted during stress testing on bicycle ergometer.

**Method:** VO2max was directly measured during step-vise increased workload on bicycle ergometer. The values of VO2max and VO2max/kg were compared to the values of W170 and W170/kg and Wmax and Wmax/kg and both regression equations and correlation calculated.

**Fig. 2 (abstract A53).**
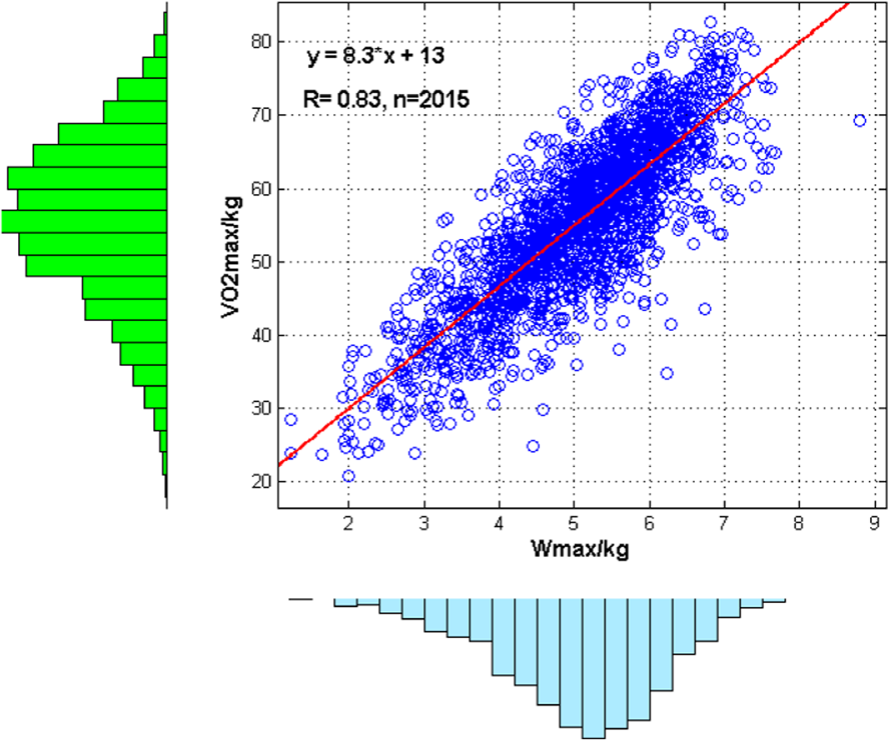
ᅟ

**Fig. 3 (abstract A53).**
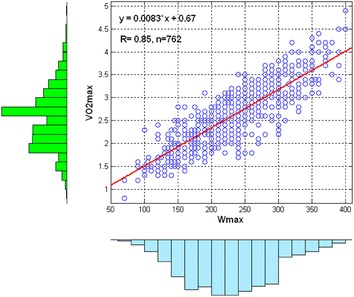
ᅟ

**Subjects:** 2777 male (2015) and female (n = 762) subjects aged 7 to 95 years were included into the study. The highest correlation between maximal aerobic capacity values VO2max and VO2max/kg was found to maximal performance in Watts (Wmax and Wmax/kg) (r = 0.92). Regression equations enable to calculate predicted VO2max and VO2max/kg with high accuracy. Figures 2 and 3 illustrate the correlation between VO2max and Wmax in men and women.

**Discussion:** Maximal aerobic capacity has been strongly and positively associated with reduced disease and mortality risk, good quality of life, performance level, and functional ability. Regular aerobic endurance exercise could reduce biological age of active individuals by 10 to 20 years with a correspondingly decreased likelihood of becoming dependent when a senior and an expressive improvement in the quality of the final years of life. However, low level of VO2max and VO2max/kg indicates that serious health threatening problems could be expected in near future.

**Conclusion:** Individual maximal aerobic capacity is an important marker of health status. Knowledge of VO2max and VO2max/kg could help to prescribe optimal volume and intensity of physical activity to every client asking for advice how to benefit from the altered lifestyle. High correlation between predicted and directly measured VO2max and VO2max/kg values enable low-cost examination of these values in the framework of preventive check-up.

## A54 The EMPOWER project: laboratory medicine and Horizon 2020

### Wytze P. Oosterhuis^1^, Elvar Theodorsson^2^

#### ^1^Atrium-Orbis, Department of Clinical Chemistry and Haematology, Heerlen, The Netherlands; ^2^Department of Clinical Chemistry, University Hospital, SE-581 85, Linköping, Sweden

##### **Correspondence:** Wytze P. Oosterhuis (w.oosterhuis@atriummc.nl) – Atrium-Orbis, Department of Clinical Chemistry and Haematology, Heerlen, The Netherlands

**Keywords:** Patient empowerment, Laboratory test results, Direct reporting, Record access, Shared decision making, Health literacy

There is a growing demand from patients to be better informed and participate more actively in treatment decisions. Initiatives to give patients access to their data, such as patient portals, reflect a patient-driven approach. The better informed patient is better equipped to participate in the medical decision process, and the terms patient empowerment and shared decision making are often used in this connection. It has been shown that a better involvement of the patient leads to an improved motivation to adhere to treatment, with better health outcome. Many general practitioners are concerned that record access will create more work, with extra consultations and telephone calls as a result of patient misunderstandings. Studies suggest the opposite – that record access can reduce resource demand. Both the European Federation of Clinical Chemistry and Laboratory Medicine (EFLM) and the Section of Laboratory Medicine of the UEMS (Union Européenne des Médecins Spécialistes) recognize the development of patient empowerment and the changing role of care providers and patients. To this end, the EFLM has established the Working Group Patient Focussed Laboratory Medicine, which took the initiative of the current project [1].

The overall aim of the project is to design, develop and evaluate an interpretative knowledge and reporting system that will inform patients so that they can comprehend the significance of their laboratory test results, enabling them to better participate in the shared decision process with their physician.

The system developed by the ICT company Medecs represents a new integrated approach to process the data from other healthcare systems and communicate the results in a flexible way to different receiving systems and individual using several advanced types of communication.

The project will help to reach a European standard in the translation of laboratory test information to information understandable for patients.

**References**

1. Watson ID, Siodmiak J, Oosterhuis WP, Corberand J, Jorgensen PE, Dikman ZG, et al. European views on patients directly obtaining their laboratory test results. Clin Chem Lab Med. 2015; doi:10.1515/cclm-2015-0056.

## A55 Personality profile manifestations in patient’s attitude to oral care and adherence to doctor’s prescriptions

### Lyudmila Y. Orekhova^1^, Tatyana V. Kudryavtseva^1^, Elena R. Isaeva^2^, Vadim V. Tachalov^1^, Ekaterina S. Loboda^1^

#### ^1^Therapeutic Dentistry Department, Pavlov First Saint Petersburg State Medical University of the Ministry of Health of the Russian Federation, Saint Petersburg, Russia; ^2^General and Clinical Psychology Department, Pavlov First Saint Petersburg State Medical University of the Ministry of Health of the Russian Federation, Saint Petersburg, Russia

##### **Correspondence:** Vadim V. Tachalov (tachalov@mail.ru) – Therapeutic Dentistry Department, Pavlov First Saint Petersburg State Medical University of the Ministry of Health of the Russian Federation, Saint Petersburg, Russia

**Keywords:** Prevention of dental diseases, Oral care, Dental status, Psychological status, Motivation for individual oral care

Preventing diseases of oral cavity, specifically periodontal diseases, is currently a high-priority issue. Despite the wide selection of individual oral care products and the abundance of information on individual oral care, the prevalence of tooth decay and inflammatory periodontal diseases remains high. Apart from knowledge of individual oral care, of great importance for patients is their capacity to develop strong motivation for full-scale hygienic procedures. Patients´ motivation is immediately interconnected with their personality specifics. This article provides the analysis on the relationship between the patient’s oral cavity status and his or her personality profile.

**Scientific objectives:** Determine relationship between patient’s personality traits and his/her oral cavity health status.

**Technological approaches:** The study was housed by the Therapeutic Dentistry Department of the First Saint Petersburg State Medical University (SMU). A total of 153 people, 18 to 24 years of age, participated in the study, with majority represented by students of SMU Faculty of Dentistry, who went through identification of dental status – through determining oral hygiene indexes (Green & Wermillion; Silness & Loe; Fyodorov & Volodkina). The study also included determining the gingival bleeding index (Saxer & Muhleman) and the papillary marginal alveolar index (PMA).

Afterwards the patients were provided with professional hygiene, individual oral care training, and recommendations on the use of individual oral care products.

In a month, the patients were invited for a repeated examination, which included determination of dental indexes.

We developed a questionnaire to obtain information about the patient’s age, sex, social status, attitudes to his/her general health status, and dental health. To identify personality traits of the respondents, we performed a survey, which included the Leary test [1], Integrative Anxiety test (IAT) [2, 3], and Locus of Control test [4], Big Five test [5].

**Results:** As a result of the study, the patients were divided into two groups: a negative dynamics group – with patients whose Green-Vermillion index increased in a month, compared to the beginning of the study; and a positive dynamics group – with patients whose GV index decreased.

Each group was analyzed using the Spearman correlation coefficient - r – for non-normal distributions. The analysis revealed weak positive (0.4-0.7), weak negative (-0.7- -0.4), strong positive (0.7-1), and strong negative (-1 - -0.7) correlation (р ≤ 0.01).

The following common factors were identified in the negative dynamics group:

1. Availability of the positive significant (р ≤ 0.01) correlation between such indicators as:

- Saxer-Muhleman index and Locus of Control Scale 4 – in the field of family relations
- Silness-Loe index and Leary 7 (cooperative style of interactions), Leary 4 (distrustful style of interactions)

2. Availability of negative significant (р ≤ 0.01) correlation between such indicators as:

- Saxer-Muhleman index and Locus of Control Scale 5 – in the field of production relations (-0.41)
- Fyodorov-Volodkina index and Integrative Anxiety Test index 10 (phobic component of anxiety) (-0.42), Locus of Control Scale 3 (control over misfortune) (-0.53), Locus of Control Scale 5 (in the field of production relations) (-0.56) , IAT 3 (-0.43), 7 (-0.44), 9 (-0.61)
- Green-Wermillion index and Integrative Anxiety Test index 4 (-0.47), 6 (-0.59), 10 (-0.45),12 (-0.41)

The following should be noted separately:

- Negative correlation relationship between frequency of dental visits and Locus of Control Scale 3 (in the field of misfortune) (-0.41)
- Attendance to preventive examinations and Leary index 1 (competitive style of interactions) (-0.54)
- Positive correlation relationship between attendance to preventive examinations and Locus of Control Scale 6 (in the field of interpersonal relations) (0.46)
- Negative correlation relationship between the tooth brushing frequency and Integrative Anxiety Test index 10 (-0.41)
- Positive correlation between the frequency of tooth brush replacement and Leary index 1 (competitive style of interactions) (0.55), 2 (aggressive-independent style of interactions) (0.47), 8 (responsible-altruistic style of interactions) (0.63), Integrative Anxiety Test index 2 (0.46)
- Negative correlation between the frequency of tooth brush replacement and Locus of Control Scale 6 (in the field of interpersonal relations) (-0.40)
- Positive correlation between the use of a tooth brush and mouthwash and Leary index 6 (dependent and obedient style of interactions) (0.41)
- Negative correlation between the right manner of cleaning teeth from the patient’s perspective and Leary index 1 (competitive style of interactions) (-0.41), 2 (aggressive-independent style of interactions) (-0.47)

In the positive dynamics group, the analysis only revealed correlation between hygienic and periodontal indexes.

**Outlook:** In the group with high oral care rates, no psychological specifics were identified. This may be indicative of the fact that the relationships between the good oral cavity health status and the patient’s psychological type were not formed, or were not manifested.

In the negative dynamics group, the following common factors were identified:

There is positive correlation between oral hygiene indexes and frequency of dental visits: the worse the oral hygiene, the more frequent the patient’s need to attend a dentist.

There is positive correlation between hygiene indexes and the use of additional hygiene products, and there is negative correlation between hygiene indexes and the use of only a tooth brush, which is indicative of the patient’s need in additional hygienic products in case of increased plaque-forming.

Inverse correlation between oral hygiene indexes and trait and state anxiety in the field of production relations (Locus of Control Scale 5), may be explained by the patient’s low level of responsibility for his/her work and oral care.

There is positive correlation between Silness-Loe index and Leary index 4 (skeptical and distrustful style of interactions), which shows that people of this type of interpersonal relations distrust doctors recommendations on oral care.

Patients with low level of anxiety replace toothbrushes less frequently.

Patients with low figures of Leary indexes 1, 2, and 8, which correspond to competitive, aggressive-independent, and responsible-altruistic styles of interactions respectively, are less careful about their health.

Patients with low Leary index 6 (dependent and obedient style of interactions) do not follow doctor’s recommendations properly.

**References**

1. Leary T. Interpersonal Diagnosis of Personality: A functional theory and methodology for personality evaluation. Ronald Press Company: New York; 1957.

2. Spielberger CD, Gorssuch RL, Lushene PR, Vagg PR, Jacobs GA. Manual for the State-Trait Anxiety Inventory. Consulting Psychologists Press, Inc.; 1983.

3. Spielberger CD, Sydeman SJ (1994). State-Trait Anxiety Inventory and State-Trait Anger Expression Inventory. In M.E. Maruish (Ed.), The use of psychological testing for treatment planning and outcome assessment. Hillsdale, NJ: Lawrence Erlbaum Associates;1994. p. 292-321.

4. Rotter JB. Generalized expectancies of internal versus external control of reinforcements. Psychol Monogr. 1966;80:1-28.

5. John O, Donahue EM, Kentle RL. The Big Five Inventory – versions 4a and 54. Berkeley: University of California at Berkeley, Institute of Personality and Social Research; 1991.

## A56 Results of an European survey on personalized medicine addressed to directions of laboratory medicine

### Mario Pazzagli (m.pazzagli@dfc.unifi.it)^1^, Francesca Malentacchi^1^, Irene Mancini (irene.mancini@unifi.it)^1^, Ivan Brandslund (Ivan.Brandslund@rsyd.dk)^2^, Pieter Vermeersch (pieter.vermeersch@uzleuven.be)^3^, Matthias Schwab (Matthias.Schwab@ikp-stuttgart.de)^4^, Janja Marc (janja.marc@ffa.uni-lj.si)^5^, Ron H.N. van Schaik (r.vanschaik@erasmusmc.nl)^6^, Gerard Siest (gerard.siest@univ-lorraine.fr)^7^, Elvar Theodorsson (elvar.theodorsson@liu.se)^8^^,9^ Chiara Di Resta (diresta.chiara@hsr.it)^10^

#### ^1^Department of Experimental, Clinical and Biochemical Sciences, University of Florence, Viale G. Pieraccini 6, 50139 Florence, Italy; ^2^Department of Biochemistry Faculty of Health Sciences, University of Southern Denmark, Vejle Hospital, Vejle, Denmark; ^3^Clinical Department of Laboratory Medicine, University Hospitals Leuven, Leuven, Belgium; ^4^Department of Clinical Pharmacology, University Hospital Tuebingen, Tuebingen, Germany and Dr. Margarete Fischer-Bosch Institute of Clinical Pharmacology, Stuttgart, Germany; ^5^Department of Clinical Biochemistry, Faculty of Pharmacy, University of Ljubljana, Ljubljana, Slovenia; ^6^Department of Clinical Chemistry, Erasmus University Medical Centre, Rotterdam, The Netherlands; ^7^University of Lorraine, UMR ISERM U1122; IGE-PCV, Genetique Cardiovasculaire, Nancy, France; ^8^Division of Microbiology and Molecular Medicine, Department of Clinical and Experimental Medicine, Faculty of Health Sciences, Linköping University, Linköping, Sweden; ^9^Department of Clinical Chemistry, Center for Diagnostics, County Council of Östergötland, Linköping, Sweden; ^10^Genomic Unit for the Diagnosis of Human Pathologies, Vita-Salute San Raffaele University, Milan, Italy

##### **Correspondence:** Francesca Malentacchi (0039 055 2758251; francesca.malentacchi@gmail.com) – Department of Clinical and Experimental Biomedical Sciences, University of Florence, Viale G. Pieraccini 6, 50139 Florence, Italy

**Keywords:** Laboratory medicine, “-omics” technologies, Personalized medicine

**Scientific Objectives:** The implementation of personalized medicine will imply a significant increase in the number of performed screening or diagnostic tests and a larger volume of data to be gathered, analyzed, and translated into information to serve as guidance for clinical decisions. Laboratory medicine is asked to play a key role in the personalized medicine approach; however, it is not clear if this development is shared by the laboratory medicine directors and if/how some activities have been already planned at the local or national level to reach this goal.

**Technological approaches:** To investigate whether laboratory medicine is able to implement new diagnostic tools, expertise and commands, and the proper state-of-the-art knowledge about personalized medicine and laboratory medicine in Europe, the joint working group “Personalized Laboratory Medicine” of the EFLM (European Federation of Clinical Chemistry and Laboratory Medicine) and ESPT (European Society of Pharmacogenomics and Personalised Therapy) societies compiled and conducted the questionnaire “Is Laboratory Medicine ready for the era of Personalized Medicine ?”.

**Results interpretation:** Forty-eight laboratories from 18 European countries participated in this survey. The answers of the participating laboratory medicine professionals indicate that they are aware that personalized medicine can represent a new and promising health model, and that laboratory medicine should play a key role in supporting the implementation of personalized medicine in the clinical setting [1,2].

**Outlook and Expert Recommendations:** Participants think that the current organization of laboratory medicine needs additional/relevant implementations such as (i) new technological facilities in - omics; (ii) additional training for the current personnel focused on the new methodologies; (iii) incorporation in the laboratory of new competencies in data interpretation and counselling; and (iv) cooperation and collaboration among professionals of different disciplines to integrate information according to a personalized medicine approach.

**Acknowledgements**

This absract is published on behalf of the European Federation of Clinical Chemistry and Laboratory Medicine (EFLM) – European Society of Pharmacogenomics and Personalised Therapy (ESPT) Joint Working Group on Personalized Laboratory Medicine (WG-PLM).

FM, IM, MS, JM, RHNvS, GS are members of the Working Group Personalized Laboratory Medicine (WG-PLM) in the European Society of Pharmacogenomics and Personalised Therapy. A Scientific Society for Individualised medicine (ESPT).

MP, IB, PV, ET, CDR are members of the Working Group Personalized Laboratory Medicine (WG-PLM) in the European Federation of Clinical Chemistry and Laboratory Medicine (EFLM).

**References**

1. Malentacchi F, Mancini I, Brandslund I, Vermeersch P, Schwab M, Marc J, et al. Is laboratory medicine ready for the era of personalized medicine? A survey addressed to laboratory directors of hospitals/academic schools of medicine in Europe. Drug Metabol Personal Ther. 2015;30:121-8.

2. Malentacchi F, Mancini I, Brandslund I, Vermeersch P, Schwab M, Marc J, et al. Is laboratory medicine ready for the era of personalized medicine? A survey addressed to laboratory directors of hospitals/academic schools of medicine in Europe. Clin Chem Lab Med. 2015;53(7):981-8.

## A57 MCI or early dementia predictive speech based diagnosis techniques

### Matus Pleva, Jozef Juhar

#### Department of Electronics and Multimedia Communications, Faculty of Electrical Engineering and Informatics, Technical university of Kosice, Letná 9, Košice, Slovakia

##### **Correspondence:** Matus Pleva (Matus.Pleva@tuke.sk) – Department of Electronics and Multimedia Communications, Faculty of Electrical Engineering and Informatics, Technical university of Kosice, Letná 9, Košice, Slovakia

**Keywords:** HCI, Speech based diagnosis, Personalized voice analysis

**Proposed techniques:** Speech disorders are one of the most common symptoms of different neurological diseases. It is obvious that the early diagnosis of these diseases could help the patient to slower the disease progression and start an intensive therapy on time, which could also help with patient social recovery. The *predictive* techniques for early dementia or speech based diagnosis of MCI patients were introduced in recent years. We propose to evaluate the speech recordings from several simple predefined tasks with subsequent automatic analysis using already tested scenarios in [1]. The patient voice answers could be automatically analyzed using methods from [2] and [1]. The textual analysis can be evaluated using several algorithms reported in [3] or [4]. The combined textual and prosodic analysis is presented in [5].

Using these methods the MCI patients can be currently diagnosed with 20 % EER (Equal Error Rate) and AD patients with 13 % EER [1], which should be decreased during the proposed evaluation period using novel speech feature manipulation techniques invented in our laboratory and extending the speech patient DB in cooperation with our international medical partners.

Widely used mobile devices equipped with microphones can be also used for early speech-based digital diagnosis. Implementation of speech analysis algorithms with adaptation function for individual user can be a part of *personalized* digital medical assistant.

**References**

1. Satt A, Hoory R, König A, Aalten P, Robert P H. Speech-Based Automatic and Robust Detection of Very Early Dementia. Proc InterSpeech. 2014; doi:10.13140/2.1.1258.8805.

2. López-de-Ipiña K, Alonso JB, Travieso CM, Solé-Casals J, Egiraun H, Faundez-Zanuy M, et al. On the selection of non-invasive methods based on speech analysis oriented to automatic Alzheimer disease diagnosis. Sensors (Basel). 2013;13(5):6730-45.

3. Roark B, Mitchell M, Hosom J, Hollingshead K, Kaye J. Spoken Language Derived Measures for Detecting Mild Cognitive Impairment. IEEE Trans Audio Speech Lang Process 2011;19(7):2081-90.

4. Fraser KC, Meltzer JA, Graham NL, Leonard C, Hirst G, Black SE, et al. Automated classification of primary progressive aphasia subtypes from narrative speech transcripts. Cortex. 2014;55:43-60.

5. Khodabakhsh A, Yesil F, Guner E, Demiroglu C. Evaluation of linguistic and prosodic features for detection of Alzheimer’s disease in Turkish conversational speech. EURASIP J Audio Speech Music Process 2015;2015:9.

**Acknowledgements**

This publication is supported by the Project implementation: University Science Park TECHNICOM for Innovation Applications Supported by Knowledge Technology, ITMS: 26220220182, supported by the Research & Development Operational Programme funded by the ERDF.

## A58 Personalized speech based mobile application for eHealth

### Matus Pleva, Jozef Juhar

#### Department of Electronics and Multimedia Communications, Faculty of Electrical Engineering and Informatics, Technical university of Kosice, Letná 9, Košice, Slovakia

##### **Correspondence:** Matus Pleva (Matus.Pleva@tuke.sk) – Department of Electronics and Multimedia Communications, Faculty of Electrical Engineering and Informatics, Technical university of Kosice, Letná 9, Košice, Slovakia

**Keywords:** HCI, Speech based diagnosis, Personalized voice analysis

**Proposed solutions:** This proposal is a description of our planned participation as a technical partner in H2020 project named iManagePrenatal for speech/voice related human machine interaction (HCI) components. The existing server based speech recognition module will be used as starting point [1]. We aim to create a new acoustic multilingual drug name database providing speech input for speech based management of drug prescription recommendation during pregnancy [2, 3]. We also plan to create a new multilingual speech command interface providing eHealth mobile application functionalities for visually impaired end-users. The last proposal is to provide multilingualism for automatic transcription of speech search query to text for smart search in the knowledge base of the eHealth portal. The last proposal includes the design and implementation of a Natural Language Processing (NLP) Text Analyser of textual queries semantics expressed by the end-user [4]. The NLP module will benefit from semantic knowledge extracted and stored in the drug databases provided by the project partners. The database analysis should go beyond the simple syntactical parsing of grammatical structures.

The advantage of the proposed system is that the language resources used for decoding the audio stream on the server side can be modified by the provider without any changes on the mobile application, so the feedback gathered during the application lifetime can be used to improve the accuracy of the components in a very short time, and without necessary end-user application upgrade. These goals require multilingual acoustical modelling cooperating with all partners’ countries. The adaptation of the end user model could help to *personalize* the application for specific needs and speech articulation.

**References**

1. Lojka M, Ondas S, Pleva M, Juhar J. Multi-thread parallel speech recognition for mobile applications. J Electr Electronics Eng. 2014;7(1):81-6.

2. Adam MP, Polifka JE, Friedman JM. Evolving Knowledge of the Teratogenicity of Medications in Human Pregnancy. Am J Med Genet C Semin Med Genet. 2011;157C(3):175-82.

3. Hall HG, Griffiths DL, McKenna LG. The use of complementary and alternative medicine by pregnant women: A literature review. Midwifery. 2011;27(6):817-24.

4. Stas J, Juhar J, Hladek D. Classification of heterogeneous text data for robust domain-specific language modeling. EURASIP J Audio Speech Music Process. 2014;2014:14.

**Acknowledgements**

This publication is supported by the Project implementation: University Science Park TECHNICOM for Innovation Applications Supported by Knowledge Technology, ITMS: 26220220182, supported by the Research & Development Operational Programme funded by the ERDF.

## A59 Circulating tumor cell-free DNA as the biomarker in the management of cancer patients

### Jiří Polívka jr.^1,2,3^, Filip Janků^4^, Martin Pešta^5^, Jan Doležal^6^, Milena Králíčková^1,2^, Jiří Polívka^3^

#### ^1^Department of Histology and Embryology in Plzen, Charles University in Prague, Plzen, Czech Republic; ^2^Biomedical Centre, Faculty of Medicine in Plzen, Charles University in Prague, Plzen, Czech Republic; ^3^Department of Neurology, Faculty of Medicine in Plzen, Charles University in Prague and Faculty Hospital Plzen, Plzen, Czech Republic; ^4^Department of Investigational Cancer Therapeutics, The University of Texas MD Anderson Cancer Center, Houston, USA; ^5^Department of Biology, Faculty of Medicine in Plzen, Charles University in Prague and Faculty Hospital Plzen, Plzen, Czech Republic; ^6^Department of Surgery, Faculty of Medicine in Plzen, Charles University in Prague and Faculty Hospital Plzen, Plzen, Czech Republic

##### **Correspondence:** Jiří Polívka jr. (polivkajiri@gmail.com) – Department of Neurology, Faculty of Medicine in Plzen, Charles University in Prague and Faculty Hospital Plzen, Plzen, Czech Republic

**Keywords:** Circulating tumor cell-free nucleic acid, Liquid biopsy, Prediction, Targeted therapy, Biomarker, Patient management, Personalized medicine

Despite a huge progress in modern oncology, advanced cancer remains poorly treatable disease and the majority of patients with advanced tumors eventually die. In the current era of personalized medicine the optimal choice of therapy depends upon the detailed analysis of the cancer genome and identification of the targetable mutations for each individual patient. Substantial limitation of this approach stems from the considerable spatial and temporal intratumor heterogeneity of advanced disease. The direct tumor biopsy remains for a long time the standard-of-care for verification of cancer type and for analysis of targetable mutations. However, the vast temporal and spatial tumor heterogeneity would virtually require multiple biopsies from primary tumor site as well as distinct metastases at different time points. This is not generally feasible because of poor medical condition of advanced cancer patients, possible clinical risks and surgical complications and also economic considerations. To overcome these limitations there is a big effort to implement novel minimal-invasive or non-invasive methods to analyze individual patient’s tumor biology and heterogeneity. One of these approaches utilizes circulating tumor cell-free nucleic acid (cfDNA) in the blood as well as cerebral spinal fluid, urine and other body fluids of cancer patients, so-called liquid biopsy. The liquid biopsy techniques offer unique opportunity to asses and monitor complex cancer disease in individual patient in a real time. In the near future, the liquid biopsy likely becomes a standard of care for early detection of the disease, assessment of molecular heterogeneity and prediction of best targeted therapy, evaluation of treatment response, monitoring residual disease together with the early detection of recurrence or assessment of resistance to given therapy.

This work was supported by MH CZ-DRO (Faculty Hospital in Plzen-FNPl, 00669806) and the project ED2.1.00/03.0076 from European Regional Development Fund.

## A60 Complex stroke care – educational programme in Stroke Centre University Hospital Plzen

### Jiří Polívka^1^, Alena Lukešová^1^, Nina Müllerová^2^, Petr Ševčík^1^, Vladimír Rohan^1^

#### ^1^University Hospital Plzen and Faculty of Medicine in Plzen, Charles University in Prague, Department of Neurology, Plzen, Czech Republic; ^2^University Hospital Plzen, Department of Quality Assessment, Plzen, Czech Republic

##### **Correspondence:** Jiri Polivka (polivka@fnplzen.cz) – University Hospital Plzen and Faculty of Medicine in Plzen, Charles University in Prague, Department of Neurology, Plzen, Czech Republic

**Keywords:** Stroke, Educational programme, Primary and secondary prevention, Patient care

**Background:** Stroke care in Czech Republic is centralised to stroke centres. Stroke centre University Hospital Plzen provides acute stroke care for 600 000 habitants of West Bohemia. Providing and sharing information on stroke is useful tool for improving stroke care.

**Summary of work:** Optional one-day course “Complex stroke care for medical students“ in our Faculty of Medicine is additional part of standard pregraduate courses of neurology since 1999. Special educational programme “Complex stroke care for nurses and paramedics” is another activity of our stroke centre. This programme started in 2001 as two-day course, updated and certified by Ministry of Health of the Czech Republic in 2009. Duration of course is now 9 days divided in two parts. First (3 days) – includes lectures and discussions on main topics – pathophysiology of stroke, diagnostics and imaging, stroke treatment, thrombolysis, mechanical recanalisation, neurosurgery, stroke and heart, stroke and diabetes, neurointensive care, speech problems, complications of stroke, depression, primary and secondary prevention, nursing, specificity of care for stroke patients, communication with patients and caregivers, legislation and regulatory, stroke care organisation, physiotherapy and occupational therapy, social assistance, ethics, education. The second part (6 days) – practice mainly in Stroke Unit and Physiotherapy Department.

**Summary of results:** 1120 medical students completed the optional course. 493 nurses, occupational therapists and paramedics from the whole country completed the special educational programme on stroke care. The course quality evaluation (usefulness, complexity, importance in daily practice) by participants is high (9.2 out of 10 points).

**Conclusions:** Multidisciplinary educational programmes for medical students as well as for nurses and paramedics consisting of lectures, discussion and practical training are useful and bring new quality to stroke care.

This work was supported by MH CZ-DRO (Faculty Hospital in Plzen-FNPl, 00669806).

## A61 Sleep apnea and sleep fragmentation contribute to brain aging

### Kneginja Richter^1,2,3^, Lence Miloseva^3^, Günter Niklewski^1,3^

#### ^1^University Clinic for Psychiatry and Psychotherapy, Paracelsus Private Medical University, Nuremberg, Germany; ^2^Georg Simon Ohm University for applied sciences, Nuremberg, Germany; ^3^Faculty for Medical Sciences, University Goce Delcev, Stip, Macedonia

##### **Correspondence:** Kneginja Richter (Kneginja.Richter@gmx.de) – University Clinic for Psychiatry and Psychotherapy, Paracelsus Private Medical University, Nuremberg, Germany

**Keywords:** Brain aging, Sleep apnea, Sleep disturbances, Sleep fragmentation

Sleep apnea is a frequent disturbance with prevalence of 3-4 % in adult man [1, 2], and is 2-9 times more prevalent in men than women [3]. The most prominent symptoms of sleep apnoea are intermittent breaks of breathing in the night (apnea) which causes general hypoxia and daily sleepiness. Risk factors for sleep apnoea are vascular hypertonia, smoking, obesitas, Diabetes mellitus and age [4]. The consequences of sleep apnea are cardiovascular diseases including heart infarctation and brain apoplexy, as well as depression and cognitive decline. The diagnosis of sleep apnea can be made by polygraphy and/or polysomnography recording in sleep labor according to the following criteria: more than 10 apneas in one hour of sleep, each with duration longer than 10 seconds.

The reason for cognitive decline in patients with sleep apnea is the intermittent hypoxia which causes disturbances of memory, attention and learning [5-8].

According to actual studies, hypoxia causes cellular damage of left hippocampus area which is one of the key brain areas for the cognition and memory [9-11].

But not only hypoxia as result of apneas can induce cognitive decline, also the fragmentation of sleep by frequent awakening caused by intermittent apneas impairs the consolidation of the memory especially in the REM (Rapid eye movement) sleep stage of the sleep.

Sleep apnea and sleep fragmentation can be significant factors for brain aging causing severe disturbances of the cognition through hypoxia of the brain and hyperarousals (stress).

Sleep apnoea and sleep fragmentation in elderly correlates with cognitive decline both in the fluid and crystal intelligence. Those elderly having sleep apnea and frequent sleep fragmentation are on risk for cognitive decline. Healthy elderly with good sleep have good cognitive reserve and delayed brain aging.

Early prevention of sleep apnea can probably protect from early brain aging.

**References**

1. Young, T, Palta M, Dempsey J, Skatrud J, Weber S, Badr S. The occurrence of sleep-disordered breathing among middle-aged adults. N Engl J Med.1993;328(17):1230–5.

2. Shepertycky MR, Banno K, Kryger MH. Differences between men and women in the clinical presentation of patients diagnosed with obstructive sleep apnea syndrome. Sleep. 2005;28(3):309–14.

3. Bozkurt MK, Oy A, Aydin D, Bilen SH, Ertürk IO, Saydam L, et al. Gender differences in polysomnographic findings in Turkish patients with obstructive sleep apnea syndrome. Eur Arch Otorhinolaryngol. 2008;265(7):821-4.

4. Guilleminault C, Silvestri R, Mondini S, Coburn S. Aging and Sleep Apnea: Action of Benzodiazepine, Acetazolamide, Alcohol, and Sleep Deprivation in a Healthy Elderly Group. J Gerontol. 1984;39(6):655-61.

5. Wolkove N, Elkholy O, Baltzan M, Palayew M. Sleep and aging: 1. Sleepdisorders commonly found in older people. CMAJ. 2007;176(9):1299-304.

6. Spira AP, Blackwell T, Stone KL, Redline S, Cauley JA, Ancoli-Israel S, et al. Sleep-disordered breathing and cognition in older women. J Am Geriatr Soc. 2008;56(1):45-50.

7. Ancoli-Israel S, Kripke DF, Klauber MR, Mason WJ, Fell R, Kaplan O. Sleep-disordered breathing in community-dwelling elderly. Sleep. 1991;14(6):486-95.

8. Salorio CF, White DA, Piccirillo J, Duntley SP, Uhles ML. Learning, memory, and executive control in individuals with obstructive sleep apnea syndrome. J Clin Exp Neuropsychol. 2002;24(1):93-100.

9. Torelli F, Moscufo N, Garreffa G, Placidi F, Romigi A, Zannino S, et al. Cognitive profile and brain morphological changes in obstructive sleep apnea. Neuroimage. 2011;56(2):787–93.

10. Macey PM, Henderson LA, Macey KE, Alger JR, Frysinger RC, Woo MA, et al. Brain Morphology Associated with Obstructive Sleep Apnea. Am J Respir Crit Care Med. 2002;166(10):1382-7.

11. Morrell MJ, McRobbie DW, Quest RA, Cummin AR, Ghiassi R, Corfield DR. Changes in brain morphology associated with obstructive sleep apnea. Sleep Med. 2003;4(5):451-4.

## A62 Personalised approach for sleep disturbances in shift workers

### Kneginja Richter^1,2,3^, Jens Acker^4^, Guenter Niklewski^1,3^

#### ^1^Univesity Clinic for Psychiatry and Psychotherapy, Paracelsus Private Medical University, Nuremberg, Germany; ^2^Georg Simon Ohm University for applied sciences, Faculty for Social Sciences, Nuremberg, Germany; ^3^Faculty for Medical Sciences, University Goce Delcev, Stip, Macedonia; ^4^Clinic for Sleep Medicine, Bad Zurzcach, Schwitzerland

##### **Correspondence:** Kneginja Richter (Kneginja.Richter@gmx.de) – Univesity Clinic for Psychiatry and Psychotherapy, Paracelsus Private Medical University, Nuremberg, Germany

**Keywords:** Shift work, Behavioral strategy, Insomnia, Fatigue, Treatment, Prevention, Health promotion

Excessive fatigue and sleep disturbances caused by shift work can lead to reduced work performance, processing errors, and accidents at work [1, 2]. Sleep disturbances among shift workers can cause absenteeism, reduced quality-of-life, and depression and rotating shifts can be a risk factor for different somatic and psychiatric diseases as f.e. cardiovascular, gastro-intestinal, metabolic, reproductive and malignant diseases [3, 4].

There are general recommendation for some coping strategies against sleep disorders associated with shift work, such as napping and exposure to bright light [5]. Still more evidence is needed for the individual approach to chronic primary and comorbid insomnia in shift worker using cognitive-behavioral techniques. These individual and personalized coping strategies based on the sleep habits of the shift worker should be considered in the workplace health promotion programs of each work environment.

**References**

1. Richter K, Niklewski G. Health Promotion and Prevention in Companies – Economic Aspects and Prevention Strategies for Shift Work Sleep Disorders. In: Healthcare Overview: New Perspectives, Costigliola V (ed) in the Book Series „Advances in Predictive, Preventive and Personalised Medicine“, Golubnitschaja O (ed), Springer Dordrecht Heidelberg New York London, 2012, doi:10.1007/978-04-007-4602-2.

2. Bajraktarov A, Novotni A, Manusheva N, Nikovska DG, Miceva-Velickovska E, Zdraveska N, et al. Main effects of sleep disorders related to shift work-opportunities for preventive programs. EPMA J. 2011;2(4):365-70.

3. Richter K, Acker J. Kamcev N, Bajraktarov S, Piehl A, Niklewski G. Recommendations for the prevention of breast cancer in shift workers. EPMA J. 2011;2:351-6.

4. Richter K. Shift Work and Breast Cancer. EPMA J. 2011;(Suppl 1):S151-5.

5. Richter K, Acker J, Scholz F, Niklewski G. Health promotion and work: prevention of shift work disorders in companies . EPMA J. 2010;1(4):611.

## A63 Medical travel and innovative PPPM clusters: new concept of integration

### Olga Safonicheva^1^, Vincenzo Costigliola^2^

#### ^1^First Moscow State Medical University named by Sechenov, Moscow, Russia; ^2^European Association for Predictive, Preventive and Personalised Medicine, Brussels, Belgium

##### **Correspondence:** Olga Safonicheva (Safonicheva.o@mail.ru) – First Moscow State Medical University named by Sechenov, Moscow, Russia

**Keywords:** Personalized medicine, Personalized prevention, Medical health wellness tourism, Medical recreation and cluster

The lifestyle of modern citizens is changing constantly: if they want to be in the epicenter of professional, scientific and cultural events – they need to travel. From the other side, people need qualitative rest after stressful and intensive work. Up-to-day tourism is phenomena of XXI century, sphere of extremely growing industry that creates about 10 % of world gross product, attracts investments and involves millions of people from different professions and qualifications into its infrastructures. This sphere is scientifically-based industry, complicated policy of global level, dialogue of civilizations and cultures. It has a lot of directions: ecotourism, cultural, sport, special event and scientific tourism; agricultural tourism, social tourism, etc. The medical (health and wellness) travel, using the factors of nature, play an important role for keeping up healthy lifestyle, preventing diseases; activating the intrinsic bio-resources of the body and increase quality of life after or together with treatment.

The impact of travel on medicine and economic development is undeniable. Medical and health travel follow the same aim – health improvement of the person, but they need different infrastructural offers and personalized approach. Medical (rehabilitation) travel has to be included into 3-level health care network: polyclinic – hospital – health resort/rehabilitation center. In this case, the patient needs the specific personalized treatment/care in specified profile departments (respiratory, cardiovascular, musculoskeletal, neurodegenerative, etc.) and inclusion of natural aero-, hydro-mineral, hydro-thermal, climate-landscape resources. Doctors may recommend personalized accompany programs for patients after treatment. In the case of healthy travel, “practically health patient” have motivation to improve of health, quality of life and have proper leisure time as well. Resorts in this case have more possibilities for integration of natural resources with SPA-programs and personalized preventive educational programs. Today we need serious scientific-based coordination between tourism services and medical health care system.

The direction of personalized medicine is dynamically developing sphere in public health care system and medical science that enables to predict individual predisposition before onset the disease, to provide targeted preventive measures and create personalized treatment algorithms tailored to the person [1, 2]. For realization of this purpose EPMA is promoting the advancement of multidisciplinary approach, dissemination of knowledge and involve a lot of different professionals and scientists for organizing new infrastructures - innovative clusters. Innovative clusters in PPPM and Medical Travel under one umbrella have to create common advanced educational programs [3], research centers, centers for transfer of scientific technologies and presenting the best practices, co-working centers for co-using of scientific equipment [4]; public health organizations, financial institutes, centers for development. All those centers have to involve specialists from medical, economical, touristic, informatics, other spheres to coordinate activities in economics of health and market-oriented products: PPP – programs, passport of health, e-medicine projects.

Several organizations were created under EU Commission support for helping in the informational, educational, communicative development, marketing and promotion: European Cluster Policy Group, European Cluster Innovation Platform. New innovative medical and travel cluster have to create new integrated information database, supported with specific software and prepared in accordance with international Standards (IFRS 8, USALI, USFRS).

Integration of medical travel services and PPPM specialists is actual challenge for new strategies: we need the common database for better coordination PPPM programs with travel offers, for better development, for social responsibility in front of our patients and nature resources.

**References**

1. Norstedt I. Horizon 2020: European perspectives in healthcare sciences and implementation. EPMA J. 2014;5(Suppl 1):A20.

2. Golubnitschaja O, Costigliola V, EPMA. General Report and Recommendations in Preventive, Predictive and Personalized Medicine 2012: White Paper of the European Association for Predictive, Preventive and Personalised Medicine. EPMA J. 2012;1(1):14.

3. Safonicheva O. New international education project in predictive, preventive and personalized. medicine. EPMA J. 2014;5(Suppl 1):A20.

4. Safonicheva O, Martynchik S. Challenges of scientific platform for medical sciences “Preventive Protection”: technological solutions. Success Natl Sci Hist J. 2015;3:102-6.

## A64 Medical travel and women health

### Olga Safonicheva (safonicheva.o@mail.ru)

#### First Moscow State Medical University by I.M. Sechenov, Moscow, Russia

**Keywords:** Medical, Travel, Resort, Weight control, Detox therapy, Anti-aging program, Psychological relaxation, Side-effects, Indications, Contra-indications

**Actuality:** Travel for health and beauty has become one of the main trend of medical travel for women. Ecological problems and stress motivate citizens combine their vocation with health travel to eco-destinations. Resorts with natural factors (thalassotherapy, mineral-, thermal- resources) and anti-aging-, anti-stress-, functional unloading diet-, weight control, detox- programs are the most popular at the health/medical travel market.

The problem of the endo-ecological crisis, toxication of the extracellular matrix and immune deficiency are being discussed by leading specialists of different branches of medicine. Practically, we can establish the new specific epidemic of endo-ecological disease, different clinical forms of which are realizing in numerous nosologic variants. In the case of the pollution of intercellular space and poor lymph circulation, delivery of nutrients and oxygen into cells is broken. The risk of development of inflammatory, degenerative, oncological processes is raising. Accumulation of non-eliminated products in the interstitial space is supposed to become the grain for development of specific pathological processes, early aging, decreasing of a life quality and a chronic fatigue syndrome. So, medical examination, creation of the personalized profiling protocols and algorithms (indications, contra-indications) for healthy individuals and patients with endo-ecological disorders to avoid the side-effects after treatment is actual problem for medical travel, including SPA-, wellness-centers and resorts.

**New protocol of investigation** to identify the symptoms, signs and markers for poor and retrograde lymph flow may help the doctors to choose the correct health program, sequence of the procedures in the women programs.

**Clinical examination** - postural tests and kinesthetic tests for “endo-ecological crisis” are necessary for identification the role of the biomechanical disturbances in the upper aperture of the chest and myotonic “tunnel-syndromes” in the cervical spine for affecting the state of lymph circulation [1]. Test for fascia kinetics is**:** edema, congestion, increasing of tissue density, the skin fold mobility restriction in sub-clavicular and axillary areas.

**The scheme of soft-tissue correction** is aimed to remove the mechanical obstacles for the lymph outflow to the main sub-clavicular collectors, release the spasmodic muscles and activate the intersticial metabolism. Relaxation of pectoralis muscles and adductors of the shoulder for removing the compression of the lymph duct as well as relaxation of the respiratory diaphragm have to precede to all the procedures in weight control programs (classic lymphatic drainage, mud application, massage treatments, etc.) to avoid side-effects for mammary glands.

**Conclusion:** Travel for health and beauty have to be supported and guaranteed by the competent staff at the SPA-, wellness centers, resorts with personalized programs (anti-stress, anti-aging detoxification), including complementary medicine.

Endo-ecological problems and overloading of the lymphatic (immune) system are the criterions for immediate restoration of the lymph circulation in the treatment protocol.

Development of new approach to health and life-quality biomarkers (“corridor of self-regulation”) and biomarkers of predisposition to non-communicable diseases is the main task for PPPM and EPMA in the field of medical travel.

**References**

1. Safonicheva OG, Safonicheva MA, Glotov AS. The role of stress in implementation of chronic fatigue syndrome. Integrative approach to postural, cognitive disorders and cerebral metabolism. EPMA J. 2014;5(S1):А149.

## A65 Continuity of generations in the training of specialists in the field of reconstructive microsurgery

### Maxim Sautin^1^, Janna Sinelnikova^2^, Sergey Suchkov^2^

#### ^1^European Clinic of Sports Traumatology and Orthopaedics, Moscow, Russia; ^2^A.I. Evdokimov Moscow State University of Medicine & Dentistry, Moscow, Russia

##### **Correspondence:** Maxim Sautin (msautin@gmail.com) – European Clinic of Sports Traumatology and Orthopaedics, Moscow, Russia

**Keywords:** Reconstructive microsurgery, Students, Workshop, Reform of education

Currently, we frequently face a generation gap problem in education. In the majority of cases, older more experienced generation teaches young people (students) which are significantly younger. There is no doubt that it provides an excellent scientific base and forms basic thought conception grounded on a long term experience of the older generation. At the same time, it must be admitted that the less the age difference between a teacher and a student is, the fewer barriers occur between them. Moreover, it provides a proper material perception and helps to avoid inconvenience in discussion.

All these facts lead to emergence of a concept which implies gradual structure at formation of education concepts. It means that a professor, a doctor, a resident, a student and a pupil are involved in the educational process. Such a concept helps to build bridges between the older and the younger generations.

The same conception was applied in the reconstructive surgery course. For the pupil it is barely possible to define the sphere of professional interests at the young age. At the same time the sooner the pupil starts to determine his professional interests, the higher his professional results might be in the future. In case the teacher is several years older than the student. It gives an opportunity for the teacher to explain all aspects of his work easily, openly and to receive a maximal feedback.

This concept was also applied in our workgroup. All required information represented in a simple and available format by means of student’s work presentation on a school basis. The pupils can directly communicate with project participants and probably become a part of the workgroup. They receive an opportunity to work with people who have just been in a status of a pupil.

Communication of the students and postgraduates is based on the same principle.

In our opinion it is time for the education reform. The main goal of this reform is hierarchy orientation in different generation’s communication. This orientation gives grounds for the professional merits upgrade and lessening of negative impact in case of professional interest uncertainty.

## A66 Telemonitoring of stroke patients – empirical evidence of individual risk management results from an observational study in Germany

### Songül Secer^1^, Stephan von Bandemer (bandemer@iat.eu)^2^

#### ^1^Westdeutsches Zentrum für angewandte Telemedizin, Duisburg, Germany; ^2^Institute for Work and Technology, Gelsenkirchen, Germany

##### **Correspondence:** Songül Secer (Songuel.Secer@wzat.de) – Westdeutsches Zentrum für angewandte Telemedizin, Duisburg, Germany

**Keywords:** Telemedicine, Secondary prevention, Stroke, Atrial fibrillation, Hypertension, Adherence

**Scientific objectives:** Stroke is one of the most frequent causes of death and disability. 20-40 percent of patients suffer from a recurrent stroke [1]. Causes are unclear etiology (cryptogenic strokes) and a lack of adherence in the treatment of risk factors such as hypertension and atrial fibrillation. The improvement of AF detection in cryptogenic strokes and monitoring of vital parameters in order to get and keep patients within therapeutic range therefore are essential. The APOOWL project was designed to test if this can be achieved by telemonitoring.

**Technological approach:** For the detection of AF in patients with cryptogenic stroke 41 patients received a holter ECG that submitted ECGs via GSM to the telemedicine center on a daily basis. 70 patients received a blood pressure device that submitted systolic and diastolic blood pressure values to the telemedicine center twice daily. 29 patients were monitored for INR values. All data were automatically integrated into an electronic patient file and evaluated by the cardiologist of the telemedicine center.

**Results interpretation:** In 6 patients with unknown history of AF acute episodes were detected. The detection rate of 15 percent corresponds to those of trials with implantable loop Recorders. Average detection time was 50.5 days (range 9 to 112). Average blood pressure values of patients have been reduced from 136/80 to 124/73 within 9 month. The reduction by 12/7 mmHg corresponds to a risk reduction for recurrent stroke by at least 36 percent [2]. In INR management time within therapeutic range was 75.27 compared to regular care between 58 und 68 [3].

**Outlook and Expert recommendation:** Telemonitoring can significantly reduce risks of recurrent stroke. Limitations of the trial are fairly small numbers of patients and lack of control group which was due to limited time and resources. Larger randomized trials also including primary prevention are recommended.

**References**

1. Sanna T, Diener HC, Passman RS, Di Lazzaro V, Bernstein RA, Morillo CA et al. Cryptogenic stroke and underlying atrial fibrillation. N Engl J Med. 2014;370(26):2478–86. doi: 10.1056/NEJMoa1313600.

2. Law MR, Morris JK, Wald NJ. Use of blood pressure lowering drugs in the prevention of cardiovascular disease: meta-analysis of 147 randomised trials in the context of expectations from prospective epidemiological studies. BMJ. 2009;338:b1665. doi: 10.1136/bmj.b1665.

3. Ruff CT, Giugliano RP, Braunwald E, Hoffman EB, Deenadayalu N, Ezekowitz MD, et al. Comparison of the efficacy and safety of new oral anticoagulants with warfarin in patients with atrial fibrillation. A meta-analysis of randomised trials. Lancet. 2014;383(9921):955–62. doi: 10.1016/S0140-6736(13)62343-0.

## A67 Women’s increasing breast cancer risk with n-6 fatty acid intake explained by estrogen-fatty acid interactive effect on DNA damage: implications for gender-specific nutrition within personalized medicine

### Niva Shapira (nivnet@inter.net.il)^1,2^

#### ^1^Institute for Nutrition Research, Beilinson Hospital, Rabin Medical Center, Petah Tikva, Israel; ^2^Ashkelon Academic College, Ashkelon, Israel

**Keywords:** Breast cancer, Polyunsaturated fatty acids (PUFA), Gender nutrition, Women health, Personalizes medicine

Women’s increasing breast cancer rates and female-male gap in cancer incidence/mortality with western diet, specifically with n-6 polyunsaturated fatty acid (PUFA) consumption, raised questions regarding ‘gender nutrition’- related health differences with the same diet, and implications for personalized nutrition. High n-6 diets, e.g. the Israeli diet – close to the Mediteranean diet and to recommendations, save for higher n-6 (≤12%kcal) – have been associated with women ranking much worse internationally for cancer mortality, all-cancer 15^th^ vs. men’s 37^th^/44 countries and 13^th^ vs. men 2^nd^-best/22, and breast, +19.2 % above a European 27-country average vs. prostate -30.4 % below; and much smaller gender differences, i.e. in female-male life expectancy, -31.6 % vs. European average, and male:female mortality ratios for all-cancer -27.1 %.

Ethnic comparisons revealed consistently higher cancer prevalence in Israeli-Jewish than Israeli-Arab women, though Arabs’ recently increased 3.0-fold more rapidly, gradually closing the ethnic gap, concurrent with their increased n-6 intake, as corn/soy oils replaced traditionally predominant olive, while Jewish n-6 consumption declined (12 to 8.8%kcal [1995-2001]), though recent Jewish intake was still +25.5 % and n-6:n-9 monounsaturated fatty acid ratio +40.4 % higher.

Cumulative research has shown females’ greater PUFA conversion, especially n-6 linoleic-acid (LA) to arachidonic acid (ARA) and to the related inflammatory/mutagenic/carcinogenic n-6 eicosanoid cascade. Estrogen's mechanistic/molecular oxidative interactions with ARA, showed further effect on DNA-adduct formation, depurination and damage. This is beyond estrogenic effects through hormonal-metabolic cascades, suggesting causal relationships between high n-6 intake, estrogen, and breast cancer, which could exacerbate other risks, including genetic predisposition, i.e. BRCA and Her2, obesity, metabolic syndrome, pre-/diabetes, and the effect of life event-related predisposition – including early menarche, late childbearing and breastfeeding – emphasizing the importance of gender-specific nutrition in primary and secondary cancer preventive/supportive strategies.

## A68 Cytobacterioscopy of the gingival crevicular fluid as a method for preventive diagnosis of periodontal diseases

### Aleksandr Shcherbakov, Anatoly A. Kunin, Natalia S. Moiseeva

#### Voronezh N.N. Burdenko State Medical University, Voronezh, Russia

##### **Correspondence:** Natalia S. Moiseeva (natazarova@yandex.ru) – Voronezh N.N. Burdenko State Medical University, Voronezh, Russia

**Keywords:** Periodontal diseases, Different markers, Preventive therapy

The recent epidemiological findings reveal a high prevalence of periodontal diseases. Thus, 60-70 % of individuals aged 20 already have gingivitis and periodontitis, and the prevalence of the diseases, especially in the case of the latter, reaches 100 % in 35-45-year-old adults. Most periodontal diseases are first diagnosed using different visual, instrumental or radiological procedures. A number of adverse events occurring in the course of periodontal disease development, such as osseous tissue destruction, are extremely difficult to treat, which, in turn, highlights the importance of periodontal diseases prevention. Given the significance of early detection of such-like diseases for further effective treatment, it is impossible not to note a high diagnostic potential of the gingival crevicular fluid (GCF) in detection of various periodontal diseases. Our investigation has summed up the available data on using the GCF markers to detect periodontal diseases at early stages.

We examined 120 individuals of both sexes aged 18 to 25 and analyzed the cytobacterioscopic, hygienic and clinical findings as well as different GCF markers. The results have shown that the early stages of the inflammatory process are characterized by the growth in the number of cocci forming a kind of clumps, the formation of pseudomycelium of yeast cells chains up to 8-10 per high power field as well as not quite mature epithelial cells with a nucleocytoplasmic ratio of 1:2. The analysis of the enzymes that may be found in the gingival crevicular fluid detected that levels of Alkaline Phosphatase (ALP), the enzyme involved in periodontal tissues destruction, vary greatly in healthy individuals, patients with gingivitis and patients with periodontitis. It should be also noted that an increase in the enzyme levels is associated with the activity of bacteria with a highly active Alkaline Phosphatase (such as Prevotella intermedia, Streptococcus sanguis, Porhyromonas gingivalis) and the increase in the number of polymorphonuclear leukocytes. This brings us to the conclusion about the priority of bacterioscopic and cytological changes in the gingival crevicular fluid as the earliest markers indicating periodontal abnormalities, which makes it possible to suggest high efficiency of the cytobacterioscopic investigation carried out to detect the initial stages of periodontal diseases according to Prof. A.A. Kunin’s approach.

## A69 Use of the pre-cured composite for preventive re-invasions in the dental hard tissues

### Bogdan R. Shumilovich, Zhanna Lipkind, Yulia Vorobieva, Dmitry A. Kunin, Anastasiia V. Sudareva

#### Voronezh State Medical University named after N.N. Burdenko, Postgraduate Dentistry Department, Voronezh, Russia

##### **Correspondence:** Bogdan R. Shumilovich (bogdanshum@gmail.com) – Voronezh State Medical University named after N.N. Burdenko, Postgraduate Dentistry Department, Voronezh, Russia

**Keywords:** Preventive medicine, Dentistry, Aesthetics, Composite, Componeer, Nano-hybrid, Pre-polymerized form, Benefits, Personalized treatment, Quality control

Aesthetic aspects of dental treatments create the emerging field for both producers and clinicians in view of permanently increasing patient requests [1, 2]. Dental caries is still the most prevalent disease as it affects up to 100% of adults. By now, all methods of the treatment of caries are highly invasive. They include the invasive removal of tooth substance with the subsequent restoring of the tooth using the direct or indirect methods. The basic material for the direct restorations is composite. However, the composite restoration besides indisputable advantages has a significant drawback – the low service life and the need for replacement of the restoration. Clinical application of the pre-polymerised forms of composite was the qualitative breakthrough in this issue [3,4].

In this study, 274 restorations were carried out. 130 restorations were carried out by using the standard form of composite and 144 restorations – by using the pre-polymerised form of composite. The following personality parameters of each restoration were taken into account in this research: Ryge criteria, hygienic index, index of the hard tissues destruction, intensity of the caries, the age, the vitality of the teeth, and the number of previous invasions.

During the analysis of the results of statistic proceeding by the method of cluster analysis the following data were discovered: the quality of treatment immediately after the restoration in the group of pre-polymerised composite was 5.52 times (p ≤ 0.05) higher than with the standard forms, and after 12 months of treatment – 6.45 times higher (p ≤ 0.05). The results of statistical processing conducted by using the associative neural network ("artificial" intelligence) have shown that the accuracy of matching the predicted and clinical results was 80.01% in both groups of patients. The programme predicted the condition of the restorations after 12 months individually for each restoration and compared the results of prediction with objective data obtained experimentally.

High accuracy of forecasting can help the practitioner during the standard check-up procedure after 3-4 weeks of the treatment to predict the condition of the restoration after 12 months and make the correction of the restoration with minimally invasion for preventing the excessive loss of dental hard tissue and re-psychological trauma of the patients.

**References**

1. Ruscher G. Direct Restoration of Lower Anterior with COMPONEER by Coltene/Whaledent. User Report – COMPONEER, 2011.

2. Besek JM. Asthetische Frontzahnkorrektur (Restauration von Verfarbten, Erodierten und Abradierten Zahnen). Dental Praxis 2011;7-8:5-13.

3. Besek JM. The great leap forward in front-tooth restoration. User Report – COMPONEER, 2011.

4. Shumilovich BR. Clinical Experience with a System of Direct Componeer (Coltene/Whaledent, Switzerland) Composite Veneers. Work difficulties and ways of overcoming them. J Health Sci 2014;2:604-11.

## A70 National eHealth system – platform for preventive, predictive and personalized diabetes care

### Ivica Smokovski^1^, Tatjana Milenkovic^2^

#### ^1^National Diabetes Committee and University Clinic of Emergency Internal Medicine and Toxicology, Skopje, Republic of Macedonia; ^2^National Diabetes Committee and University Clinic of Endocrinology, Diabetes and Metabolic Disorders, Skopje, Republic of Macedonia

##### **Correspondence:** Ivica Smokovski (ivica.smokovski@gmail.com) – National Diabetes Committee and University Clinic of Emergency Internal Medicine and Toxicology, Skopje, Republic of Macedonia

**Keywords:** National eHealth System, Type 2 diabetes, Republic of Macedonia

National eHealth System, covering all citizens and all healthcare levels in Republic of Macedonia, was introduced in July 2013, has been internationally recognized System for successful reduction of waiting times and instrumental in the management of national healthcare resources.

For the first time, National Diabetes Committee, formed in February 2015 according to the Law on healthcare and being overall responsible for the diabetes care in the country, was able to derive exact figures on the national diabetes prevalence from the System, instead of extrapolations used before, serving as a basis for development of strategies for prediction and prevention of diabetic complications, as well as for personalized diabetes care.

Number of diabetes cases identified through the National eHealth System in June 2015 was 84,568 (4.02 % of total population), 36,119 males (3.42 % of total male population) and 48,449 females (4.61 % of total female population). Age stratified diabetes prevalence was as follows: less than 20 years – 549 cases (0.11 % of respective population), 20-39 years – 3,202 (0.49 %), 40-59 years – 26,561 (4.58 %), 60-79 years – 48,470 (14.57 %), 80 years or more – 5,786 (12.96 %).

Addition of parameters for metabolic control and diabetic complications in the System is under way, further facilitating the modeling of diabetes treatment, metabolic control and the outcomes. Inclusion of pre-diabetes patients (IGT and IFG) is also planned, thus providing opportunity to also focus healthcare activities for prevention of progression into overt type 2 diabetes.

## A71 The common energy levels of Prof. Szent-Györgyi, the intrinsic chemistry of melanin, and the muscle physiopathology: Implications in the context of preventive, predictive, and personalized medicine

### Arturo Solís-Herrera^1^, María del Carmen Arias-Esparza^1^, Sergey Suchkov^2^

#### ^1^Human Photosynthesis Study Center, Av. Aguascalientes Norte 607, Pulgas Pandas Sur, Aguascalientes, México; ^2^I.M. Sechenov Firts Moscow State Medical University and A.I. Konstantinov Moscow State Medical & Dental University, Moscow, Russia

##### **Correspondence:** Arturo Solís-Herrera (comagua2000@yahoo.com) – Human Photosynthesis Study Center, Av. Aguascalientes Norte 607, Pulgas Pandas Sur, Aguascalientes, México

**Keywords:** Muscle diseases, ATP, Myosin, Energy, Melanin, Water dissociation, Preventive medicine

**Scientific objectives:** The impairment of the muscle functions by diseases not usually confined to muscular tissue, are a frequent cause of consultation. Weakness, pain, cramps, rigidity, loss of muscular control, myoclonus (twitching, spasming) and myalgia (muscle pain) are common symptoms of muscle disease. The main sequences of events during muscle contraction are the binding of ATP to the S1 unit (the catalytic unit of myosin), and the immediate reaction producing ADP and Pi provides the free energy to move the S1 unit into the strained position. ADP and Pi remains attached to the S1. Myosin is an enzyme, specifically an ATPase [1]. Myosin S1 works like other enzymes but the conformational change is magnified. Shortening requires ATP hydrolysis and myosin binding to actin is essential for rapid ATP hydrolysis.

However, this happy and simple explanation is contradicted by some interesting observations: Relaxed muscle has low ATPase activity as if myosin does not bind to actin. Relaxed muscle is easily extensible (low stiffness) as if myosin does not bind to actin. Furthermore, neither myosin nor actin is contractile [2]. The free energy of the system drops in contraction. The relaxed state is the high-energy metastable state, and the contracted state the low energy stable state [2].

By other side, ATPase activity is bound up with myosin, but only a very small fraction of myosin molecules are endowed with such activity. Thereby question remains how the energy liberated by a molecule can be communicated to a great number of similar molecules. The common energy levels give an easy answer [3].

The unraveling of the intrinsic property of melanin to transform light energy into chemical energy through the water dissociation [4] fits very well with the common energy levels of Prof. Szent-Gyorgyi.

**Results interpretation:** Relaxed muscle is a molecular state ordered than a contracted muscle; therefore more order requires more energy. This is almost impossible to explain if we thought that glucose and ATP is therefore a source of energy. The finding that melanin transforms light energy into chemical energy by means of the dissociation of the water molecule, releasing hydrogen diatomic and high-energy electrons, breaks the paradigm and lays the biochemical foundation of the concept of the Prof. Szent-Györgyi about common energy levels.

**Outlook and Expert recommendations:** Understand that the relaxed muscle is a state of energy higher that a contracted muscle opens a new scenario in the study, diagnosis, and treatment of muscle disorders. It is shocking to understand that a contracted muscle represents one energy level less than a relaxed muscle. Understanding the true nature of muscle means to better understand health and disease of the same, which allows a better medicine preventive, predictive and personalized.

**References**

1. Engelhardt WA, Ljubimowa MN. Myosine and Adenosinetriphosphatase. Nature. 1939;144:668-9.

2. Szent-Gyorgyi A. Free-energy relations and contraction of actomyosin. Muscular Contraction. Acad. Press, New York, 1947.

3. Szent-Gyorgyi A. Towards a new biochemistry? Science 1940;93(2426):609-11.

4. Solís-Herrera A, Arias-Esparza MC, Solís-Arias RI, Solis Arias P, Solis Arias MP. The unexpected capacity of melanin to dissociate the water molecule fills the gap between life before and after ATP. Biomed Res. 2010;21(2):224-6.

## A72 Plurality and individuality of hepatocellular carcinoma: PPPM perspectives

### Krishna Chander Sridhar, Olga Golubnitschaja

#### Department of Radiology, Univeristy of Bonn, Bonn, Germany

##### **Correspondence:** Olga Golubnitschaja (olga.golubnitschaja@ukb.uni-bonn.de) – Department of Radiology, Univeristy of Bonn, Bonn, Germany

**Keywords:** Predictive preventive personalized medicine, Innovative screening methods, Patient-specific therapy, Multilevel diagnostics, Molecular markers, CTCs and CNAPS, Stratification

**Fig. 4 (abstract A72).**
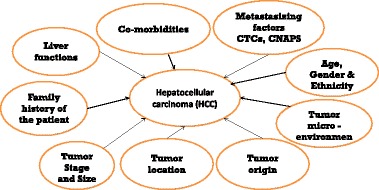
Factors to be considered for individualized patient profiles providing personalized therapy in HCC patient cohort

Hepatocellular Carcinoma (HCC) may arise due to various risk factors such as genetic predisposition, chronic hepatic infections, alcohol abuse, hemochromatosis, cirrhosis, obesity, fatty liver disease, etc. [1, 2]. Low mean 5-year survival rate (15 %) and high treatment costs imply the need for more effective treatments tailored to the person [3]. The predictive, preventive and personalized medicine (PPPM) aims at paradigm change in management of HCC from delayed to advanced approaches creating innovative screening programs, early/predictive diagnostics and targeted therapy [1]. For that, better patient stratification is needed utilizing multilevel diagnostics. Molecular mechanisms which underlie tumor manifestation, progression and aggressiveness may vary substantially in HCC depending on a number of factors such as localization of the primary tumor, tumor’s characteristics / sub-type, family history, life-style and co-morbidities. Consequently, a creation of individualized patient profiles is of particular importance in patient stratification, predictive/early diagnostics and targeted treatments of HCC (see Fig. 4). Further, highly sensitive and specific biomarker-panels play a crucial role in sub-categorization of HCC, e.g. to distinguish between local and distanced metastatic activities, where the latter are functionally linked to circulating entities such as circulating tumour cells (CTC) and circulating nucleic acids in plasma and serum (CNAPS) [4].

The integrative PPPM-strategies advancing HCC management (both curative and palliative) may have great impacts on improved screening of individuals at risk, life-quality of targeted patient cohorts, better healthcare economy and benefits to diagnostic and pharmaceutical industry.

**References**

1. Berliner L, Lemke HU (eds.). An Information Technology Framework for Predictive, Preventive and Personalized Medicine: A Use-Case with Hepatocellular Carcinoma. In: Advances in Predictive, Preventive and Personalised Medicine, Vol 8in Cham: Springer International Publishing; 2015

2. Fox RK. Surveillance for Hepatocellular Carcinoma. Hepatitis C Online, http://www.hepatitisc.uw.edu/go/evaluation-staging-monitoring/surveillance-hepatocellular-carcinoma/core-concept/all (2013) Accessed 15 Aug 2015.

3. Thein HH, Isaranuwatchai W, Campitelli MA, Feld JJ, Yoshida E, Sherman M, et al. Healthcare costs associated with hepatocellular carcinoma: a population-based study. Hepatology 2013;58(4):1375-84.

4. Zhao YJ, Ju Q, Li GC. Tumor markers for hepatocellular carcinoma. Mol Clin Oncol. 2013;1(4):593-598.

## A73 Strategic aspects of higher medical education reforms to secure newer educational platforms for getting biopharma professionals matures

### Maria Studneva^1^, Sihong Song^4^, James Creeden^5^, Мark Мandrik^1^, Sergey Suchkov^1-3,6,7,8^

#### ^1^I.M. Sechenov First Moscow State Medical University (PMGMU), Moscow, Russia; ^2^A.I. Evdokimov Moscow State University of Medicine & Dentistry (MGMSU), Moscow, Russia; ^3^European Association for Predictive, Preventive and Personalised Medicine (EPMA), Brussels, Belgium; ^4^Department of Pharmaceutics, University of Florida College of Pharmacy, Gainesville, FL, USA; ^5^Roche Diagnostics Ltd., Rotkreuz, Switzerland, and Roche Diagnostics Division, Basel, Switzerland; ^6^UCC (United Cultural Convention), Cambridge, UK; ^7^AMEE, Dundee, UK; ^8^New York Academy of Sciences, NY, USA

##### **Correspondence:** Maria Studneva (maria.studneva@gmail.com) – I.M. Sechenov First Moscow State Medical University (PMGMU), Moscow, Russia

**Keywords:** Systems biology, Predictive preventive personalized medicine (PPPM), Lifelong (continuous) education, Interactive training method, Translational medicine, High tech

**Background:** This research elucidates the creation of new trends in medicine and biopharmacy using the latest achievements in basic sciences and high technology areas, as well as the development of a concept and thus the strategy for deeply-set transformations in the field of healthcare and medical education [1].

**Materials and Methods:** The strategy for innovative development in Russia is based on implementation of intellectual potential of the domestic personnel resources into the practice and economy and setting high requirements to the quality of the professional graduate and post-graduate education [2]. An innovative model for the development of the healthcare system provides for close cooperation between the healthcare system, medical science, government and business community, planning of scientific medical research depending on the needs of healthcare service, active introduction of the research outcome into medical practice, as well as targeted training of specialists capable to ensure implementation of the achievements [3].

**Results:** The project of modernization of the Russian medical education system spells out four priority areas:

(i). change over to an ongoing professional education;
(ii). improvement of quality of vocational education;
(iii). ensuring investment attractiveness of education;
(iv). reform of general (secondary-level) education [1].

Within the framework of the Project we consider fundamental principles to getting the pre-university, university, postgraduate and basic medical training programs restructured and the development of fundamentally new generation interdisciplinary programs, focused on training and retraining of specialists in the PPPM-related areas and also the main issues arising from the implementation of the reform. In implementing the principle of continuity of an ongoing education a model of multi-stage training of a specialist in the PPPM-related areas is being built, which is characterized by a stepwise process of individual development going over (*personalized maturation of the professional*), while information is learned, from one level of an ongoing training to another one [4].

**Conclusions:** Implementation of the reforms of the higher medical education will enable to achieve and maintain the quality of training of professionals up to the worldwide standards as applicable to Hi-Tech Leves, as well as their academic, professional and inter-regional mobility.

**References**

1. Strategy of Development of Medical Science in RF for a Period till 2025. 2013. http://www.biometrica.tomsk.ru/SRMN_2025.pdf. Accessed 10 May 2015

2. Egorov NS, Oleskin AV, Samuilov VD. Biotechnology: educational aid for higher educational institutions. Book 1: Problems and Perspectives. Moscow: Vysshaya Shkola; 1987.

3. Pshenichnikova AB. Preparation of Scientific Research Personnel in Accordance with Magister Program 550822 Molecular and Cellular Biological Technology. Moscow; 2003.

4. Sapunov MB, Fedorov IB. Higher Professional Education – Synthesis of Theory and Practice. Moscow: Bauman MSTU; 2009

## A74 Overview of the strategies and activities of the European Federation of Clinical Chemistry and Laboratory Medicine, (EFLM)

### Elvar Theodorsson, EFLM

#### IKE/Klinisk kemi, Lab1, Hus 420, Plan 11, Universitetssjukhuset, SE-581 85 Linköping, Sweden

##### **Correspondence:** Elvar Theodorsson (Tel: +46(0)101033295 or +46(0)13286720; elvar.theodorsson@liu.se) – IKE/Klinisk kemi, Lab1, Hus 420, Plan 11, Universitetssjukhuset, SE-581 85 Linköping, Sweden

**Keywords:** Laboratory medicine, Clinical chemistry, Education, Health care, Patient care, Quality management, Accreditation

The European Federation of Clinical Chemistry and Laboratory Medicine (EFLM) connects National Societies of Clinical Chemistry and Laboratory Medicine and creates a platform for all European “Specialists in Laboratory Medicine”. EFLM provides European leadership in Clinical Chemistry and Laboratory Medicine to national professional societies, the diagnostic industry and to governmental and non-governmental organizations in order to serve the public interest in health care. EFLM currently comprises the national societies of 40 countries and represents the International Federation of Clinical Chemistry and Laboratory Medicine (IFCC) in Europe.

The *vision and mission* of EFLM is to enhance patient care and improve outcomes by promoting and improving the scientific, professional and clinical aspects of clinical chemistry and laboratory medicine and to ensure effective representation of laboratory medicine both at European Union level and to other pan-European and sub-regional bodies.

The main *activities* of EFLM relate to education, research, development of the profession, requirements for competence, quality and accreditation of laboratories, organisation of congresses, and publications.

The *operational structure* of EFLM consists of an Executive Board (EB) and five Committees (C)

The *Communications Committee* is responsible for communicating EFLM’s activities and results to its membership, related partner organizations and the public via EFLM’s website and Newsletter.

The *Education and Training Committee* has general responsibility for the postgraduate training aspects of the work of EFLM in the field of Clinical Chemistry and Laboratory Medicine focused on laboratory medicine professionals, health care professionals and patients, in liaison with the Education and Management Division of IFCC, and also with UEMS (European Union of Medical Specialists). The Committee organizes regional and sub-regional conferences, workshops and postgraduate continuing education courses in association with relevant national societies.

The *Profession Committee* is responsible for matters of professional regulation and certification (via the EC4 Register of European Specialists in Clinical Chemistry and in Laboratory Medicine), and the promotion of the profession in Europe at government level, and to patients and clinical users. It liaises with CEPLIS (European Council of the Liberal Professions) and with the European Commission and Parliament on professional matters. It takes the lead in developing pan-European professional and ethical standards. It also liaises with UEMS (European Union of Medical Specialists) on the roles and responsibilities of medical and scientific practitioners of the discipline.

The *Quality and Regulations Committee* supports the establishment of effective accreditation schemes and quality management systems in all European countries and liaises with ISO, CEN and the European Accreditation body (EA).

The *Science Committee* focuses on promotion of research that translates the scientific results of laboratory medicine to clinical applications and improves patient outcomes through the appropriate use and interpretation of laboratory data in clinical practice.

The committees carry out their tasks via Working Groups (WG) and Task Finish Groups (TFG).

## A75 New spectroscopic techniques for point of care label free diagnostics

### Syed A. M. Tofail (tofail.syed@ul.ie)

#### Department of Physics and Energy, and Materials and Surface Science Institute (MSSI), University of Limerick, Ireland

**Keywords:** Personalised medicine, Spectroscopy, Spectro-microscopy, Microwave, Infrared, Biomarkers

Mid infrared and microwave radiation based spectroscopy and spectro-microscopy tools techniques can detect and/or image characteristic information of biomolecules and structures without the use of any fluorescent marker or label. This approach, when fully developed, can be of huge benefit for both *in vitro* and *in vivo* diagnostics. At the beginning, we show how microwave spectroscopy can be used for detecting specific binding of proteins. We will present the case of streptavidin binding to biotinylated protein A. The method of detection was then used to monitor the adsorption of two other proteins: cytochrome c and glucose oxidase on the surface of a specifically designed microwave sensor. The response of the sensor was also tested on different substrate materials such as smooth, conductive (gold) and on rough, insulating (hydroxyapatite) surfaces [1]. The success of detection of protein binding on these substrates will facilitate the use of simple surfaces for cheap and fast detection of bioanalytes. This is in contrast to current commercially available techniques such as surface plasmon resonance (SPR), which requires expensive sensor chip and elaborate sample preparation. We will also discuss how high resolution infrared spectro-microscopy can be used for fast imaging of proteins, cells and tissues [2-4]. High brightness picosecond fibre lasers coupled with a synchronously pumped (SP)-optical parametric oscillator (OPO) allows very fast imaging of disease related positive and negative biomarkers in the mid infrared (2.5 - 14 micron wavelength, 4000-700 cm^-1^ frequency). When fully developed, these techniques can potentially revolutionise diagnosis at hospital outpatient services and at the General Practitioners.

**References**

1. Salazar-Alvarez M, Korostynska O, Mason A, Al-Shamma'a A, Cooney JC, Magner E, et al. Label free detection of specific protein binding using a microwave sensor. RSC Analyst.2014;139:5307.

2. Daly S, Mouras R, Silien C, Tofail SAM. Label free Infra-Red nanoscopy: Impact on biology and medical devices. In: Electrically active materials for medical devices. Bauer J, Tofail SAM, editors, London: World Scientific-Imperial College Press. 2016. (in press).

3. Pita I, Hendaoui N, Liu N, Kumbham M, Tofail SAM, Peremans A, et al. High resolution imaging with differential infrared absorption micro-spectroscopy. Optics Express. 2013;21:25632.

4. Silien C, Liu N, Hendaoui N, Tofail SAM Peremans A. A framework for far-field infrared absorption microscopy beyond the diffraction limit. Optics Express. 2012;20:29694.

## A76 Tumor markers for personalized medicine and oncology - the role of laboratory medicine

### Ondřej Topolčan^1^, Judita Kinkorová^1^, Ondřej Fiala^1^, Marie Karlíková^1^, Šárka Svobodová^1,2^, Radek Kučera^1^, Radka Fuchsová^1^, Vladislav Třeška^1^, Václav Šimánek^1^, Ladislav Pecen^1^, Jan Šoupal^2^, Štěpán Svačina^2^

#### ^1^Faculty Hospital in Pilsen and Faculty of Medicine in Pilsen, Charles University Prague, Pilsen, Czech Republic; ^2^General University Hospital and 1^st^ Faculty of Medicine in Prague, Prague, Charles University Prague, Czech Republic

##### **Correspondence:** Ondřej Topolčan (TOPOLCAN@fnplzen.cz) – Faculty Hospital in Pilsen and Faculty of Medicine in Pilsen, Charles University Prague, Pilsen, Czech Republic

**Keywords:** Tumor marker, Laboratory medicine, Personalized medicine, Cancer, Prediction, Prognosis

**Background**: Tumor markers have been currently used only with limited indication for the clinical routine practice.

**Study Aims:** The main aim of our laboratory is to promote the use of tumor markers for optimization of the diagnostic process and for therapy choice and therapy monitoring.

**Methods and Materials / Patients**: Results of the serum tumor markers from 2 000 patients monitored in the Faculty Hospital in Pilsen have been retrospectively evaluated. The following markers have been evaluated: CEA, AFP, mucin biomarkers cytokeratin soluble fragments and proliferative tumor markers. Markers were assessed during the primary diagnosis and during the follow-up. Serum markers were always correlated with clinical status and with imaging techniques. The following cancers were evaluated: lung, breast, colorectal and prostate cancer.

**Results:** Optimal algorithms were proposed pro for diagnostic optimization a for therapy effect prediction and for prognosis estimation in relationship to overall survival and disease free interval. Computer software is used for marker evaluation. Typical case reports will be also demonstrated.

**Conclusions:** Proposed multidisciplinary algorithm represents a typical example for personalized approach to the cancer patient. Clinical and economical benefit of these proposed algorithms is significant.

*Supported by Ministry of Health, Czech Republic - conceptual development of research organization (Faculty Hospital in Pilsen - FNPl, 00669806).*

## A77 Modern medical terminology as a driver of the global educational reforms

### Evgeniya Tretyak^1^, Maria Studneva (maria.studneva@gmail.com)^2^, Sergey Suchkov (ssuchkov57@gmail.com)^2,3^

#### ^1^Clinical Hospital 86, Federal Medical Biological Agency of Russia, Moscow, Russia; ^2^I.M. Sechenov First Moscow State Medical University, Trubetskaya Str. 8-2, Moscow, 119991, Russia; ^3^A.I. Evdokimov Moscow State University of Medicine and Dentistry, Delegatskaya Str. 20/1, Moscow, 127473, Russia

##### **Correspondence:** Evgeniya Tretyak (tretyak_eugenia@mail.ru) – Clinical Hospital 86, Federal Medical Biological Agency of Russia, Moscow, Russia

**Keywords:** Modern medical terminology, Encyclopedic dictionary, Paradigm shift, Educational reform

Predictive, preventive and personalized medicine (PPPM) meets the current need in the development of innovative approaches to the training of interdisciplinary medical specialists which requires educational reform which paves the way to the creation of innovative infrastructure. The starting point is the restructuring of terminological base as the understanding of modern medicine is impossible without understanding the terminology.

**Scientific objectives:** Innovations in science and advanced technologies are accompanied by the burst of novel terms. They undergo numerous modifications, improve and become more complicated for being understood. The extent of this renewal is not in line with mental status of specialists thus obstructing the understanding of current progress. This determines the need of the adequate representation in ethnical languages.

In Russian scientific community, English glossaries are amongst the main sources to renew medical vocabulary (more than 90 % of scientific datasets is published in English). Regular medical dictionaries are unable to cover the spectrum of conceptual terminology as vocabulary capacity is significantly limited.

A similar situation is observed in the field of textbook preparing and writing on novel hi-tech medical disciplines including PPPM. The principles and technological armamentarium of this interdisciplinary global model are an enigma for the medical community. This requires the development of novel vocabularies and formal dictionaries which incorporate conceptual terminology, explanations and illustrations.

**Technological approaches:** We attempt to create an English-Russian Series of Encyclopedic Dictionaries regarding PPPM and related and interdisciplinary areas to secure the understanding and comprehending of terms and their combinations.

**Results interpretation:** Modern medical terminology (MMT) puts forward PPPM strategies. It will serve as an impulse for further advancements by connecting scientists all across the world and creating an environment for information exchange, generation of new ideas and acceleration of applications. MMT uncovers medical innovations and potentially changes the way treatments are discovered and used. It propagates PPPM concept among healthcare professionals, governmental institutions, educators, and public. It creates a consolidated platform and provides implementation of the achievements for an effective advancing of healthcare.

**Expert recommendations:** MMT creates new measures to conduct important information for all PPPM relevant persons and shifts the paradigm from delayed reactive care to predictive and preventive medical care.

## A78 Juvenile hypertension; the relevance of novel predictive, preventive and personalized assessment of its determinants

### Francesca M. Trovato^1^, Giuseppe Fabio Martines^1^, Daniela Brischetto^1^, Daniela Catalano^1^, Giuseppe Musumeci^2^, Guglielmo M. Trovato^1^

#### ^1^Department of Clinical and Experimental Medicine, University Hospital of Catania, Catania, Italy; ^2^Department of Bio-Medical Sciences, Human Anatomy and Histology Section, School of Medicine, University of Catania, Catania, Italy

##### **Correspondence:** Guglielmo M. Trovato (guglielmotrovato@unict.it) – Department of Clinical and Experimental Medicine, University Hospital of Catania, Catania, Italy

**Keywords:** Environmental risk factors, Behavioral factors, Nutrition, Arterial hypertension, Predictive preventive personalized medicine, Obesity, Salt intake

Arterial hypertension can have its origins in juvenile behaviors and factors. The most relevant are familiar and environmental risk factors, overweight-obesity, and salt intake. Since several other environmental and behavioral factors can interfere we challenged if overweight-obesity, smoking, alcohol, sleep deprivation, coffee and salt intake are associated with arterial hypertension.

**Methods:** A group of consecutive youngster were enrolled and assessed by clinical visit and interview, measuring blood pressure by an electronic oscillometric device in two different positions, sitting and standing, and then, 5 minutes after standing and one week apart. Body weight and height were measured, and BMI calculated; salt intake was categorized as <3.0 g/day, >3.0 < 6.0 g./day and ≥6.0 g/day. Cigarette smoking, alcohol habits (at least twice a week >20 g/day), habitual use coffee and caffeine beverages use (any quantity), and sleep deprivation (defined by the 25^th^ lower percentile of average of hours of sleep in the week of observation) were recorded. Hypertension was defined as systolic BP ≥ 140 mmHg and /or diastolic BP ≥ 90 mmHg. Odds ratio was calculated as the hazard of the factors vs. hypertension.

**Results:** 833 youngsters, age 23.40 ± 5.39 years 1200 were 14-20 years old (adolescents) age 18.31 ± 1.96 years, 1282 were 21-28 years old age 23.57 ± 2.23 years and 546 were 29-35 years old age 32.33 ± 2.17 years were enrolled; men were 1167, women were 1666. The most relevant risk factor for higher blood pressure is overweigh-obesity (OR 3.815; 95 % CI 2.329-6.249), cigarette smoking (OR 1.949; 95 % CI 1.235-2.415), coffee and caffeine beverages use (OR 1.727; 95 % CI 1.235-2.415), alcohol habits (OR 1.462; 95 % CI 1.093-1.956), sleep deprivation (OR 1.441; 95 % CI 1.077-1.930), and salt use above 6 g/day (OR 1.423; 95 % CI 1.063-1.904). By MLR these factors explain the level of systolic blood pressure.

**Conclusion:** Overweight-obesity is still the most relevant risk factor of arterial hypertension also in youngsters. The concurrent, less relevant effectors, apart cigarette smoking, alcohol, salt intake and caffeine, can be many, including sleep deprivation, which is emerging as an indicator of environmental and behavioural health unfavourable effects. A comprehensive articulated predictive, preventive and personalized medicine approach should be further investigated.

## A79 Proteomarkers Biotech

### George Th. Tsangaris^1,2^, Athanasios K. Anagnostopoulos^1,2^

#### ^1^Proteomarkers Biotech P.C., Athens, Greece; ^2^Proteomics Research Unit, Biomedical research Foundation of the Academy of Athens, Athens, Greece

##### **Correspondence:** George Th. Tsangaris (gthtsangaris@bioacademy.gr; gthtsangaris@proteomarkers.com) – Proteomics Research Unit, Biomedical research Foundation of the Academy of Athens, Athens, Greece

**Keywords:** Proteomics, Mass spectrometry, Non-invasive prenatal diagnosis, Biomarkers, Pregnancy complications, Down syndrome, Diagnostic kit

Proteomarkers Biotech (PMB) is an innovative biotechnology-oriented company specialized in the non-invasive prenatal test of fetal health and pregnancy complications. The main goal of the company is the commercial exploitation of the state-of-the-art technologies of proteomics and mass spectrometry, which nowadays used on the biomarker identification and validation [1-3]. PMB will develop, produced and promote to the market innovative diagnostic kits in competitive prices, targeting a dominant position in the local and international marker.

The produced kits of PMB concern with the detection and quantitation of proteins (biomarkers) in the maternal peripheral blood and they suggested a possible embryonal abnormality (Down syndrome, Turner syndrome, Klinefelter syndrome, etc) or the possibility of development pathological situations during pregnancy such as pre-eclampsia, premature delivery, intra-uterus infections etc. Through these kits of prenatal test, PMB introduce a series of innovations and improvements in technological and financial level, as well as in the time of result receipt [4, 5]. Namely:

1. Reliability: High (>95 %) for all pregnancies independently of the risk
2. Sampling: peripheral blood
3. Low sample volume: 10 μl
4. Technology: Mass spectrometry (high sensitivity and specificity)
5. Single tube diagnosis of multiple disorders (embryonic abnormalities with simultaneous existence of pregnancy complications)
6. Fast (next day result)
7. Early application in pregnancy (week 10^th^-12^th^)
8. Low cost: at least 30 % lower compared to current methodologies

Potential customers of PMB products are all pregnant women who want fast, low cost and with high accuracy to determine whether their fetus suffering from a chromosomal abnormality or if they are at risk of developing complications during pregnancy. PMB will supply kit with diagnostic laboratories with MS who will perform these tests.

The main objective of PMB is the rapid establishment in Greek market as first step of its development and a-proof-of-concept at a market level its rapid expansion into the European and international market. It is mentioned that PMB has close relationships with companies and entities providing health services both in Greece and abroad.

**References**

1. Kolialexi A, Tsangaris GT, Papantoniou N, Anagnostopoulos AK, Vougas K, Bagiokos V, et al. Application of proteomics for the identification of differentially expressed protein markers for Down syndrome in maternal plasma. Prenat Diagn. 2008;28(8):691-8.

2. Heywood W, Wang D, Madgett TE, Avent ND, Eaton S, Chitty LS, et al. The development of a peptide SRM-based tandem mass spectrometry assay for prenatal screening of Down syndrome. J Proteomics. 2012;75(11):3248-57.

3. Kolialexi A, Anagnostopoulos AK, Tounta G, Antsaklis A, Mavrou A, Tsangaris GT. Biomarker development for non-invasive prenatal diagnosis of fetal aneuploidies: predictive reliability and potential clinical application. EPMA J. 2011;2(2):157-61.

4. Anagnostopoulos A, Tsangaris GT. Serum amyloid-p (SAP), a potential biomarker for Down syndrome fetuses prevention in maternal plasma. EPMA J. 2014;5(Suppl 1):A98.

5. Kolialexi A, Anagnostopoulos AK, Papantoniou N, Vougas K, Antsaklis A, Fountoulakis M, et al. Potential biomarkers for Turner in maternal plasma: possibility for noninvasive prenatal diagnosis. J Proteome Res. 2010;9(10):5164-70.

## A80 Proteomics and mass spectrometry based non-invasive prenatal testing of fetal health and pregnancy complications

### George Th. Tsangaris^1,2^, Athanasios K. Anagnostopoulos^1,2^

#### ^1^Proteomarkers Biotech P.C., Athens, Greece; ^2^Proteomics Research Unit, Biomedical research Foundation of the Academy of Athens, Athens, Greece

##### **Correspondence:** George Th. Tsangaris (gthtsangaris@bioacademy.gr; gthtsangaris@proteomarkers.com) – Proteomics Research Unit, Biomedical research Foundation of the Academy of Athens, Athens, Greece

**Keywords:** Proteomics, Mass spectrometry, Non-invasive prenatal diagnosis, Biomarkers, Pregnancy complications, Fetal chromosomal abnormalities, Down syndrome, Turner syndrome, Prediction, Serum amyloid-p, SAP

In the context of human reproduction research, it is widely accepted that the “holy grail” of fetal diagnostics, is the discovery of biomarkers present in the individuals’ serum/plasma that could accurately predict the occurrence of fetal abnormalities and/or pregnancy complications. Maternal blood is the biological fluid currently attracting attention of researchers, as it aspires to attribute evolution in non-invasive prenatal testing (NIPT), thus improving our understanding over the feto-maternal pathologies. Nowadays the application of state-of-the-art technologies of proteomics and mass spectrometry, have been extensively used for biomarker identification on the first trimester of pregnancy producing results concerning a variety of fetus abnormalities (Down Syndrome, Turner Syndrome, Klinefelter syndrome *etc.*) and pregnancy complications (preeclampsia, intra-uterus infections, IUGR *etc.*). Down syndrome (DS) is the most common chromosomal abnormality in pregnancy. Application of proteomics for the identification of potential biomarkers for the NIPT of DS has revealed that the protein Serum amyloid-p (SAP) could act as a reliable biomarker for DS fetuses at the 10^th^-12^th^ and 15^th^-16^th^ week of pregnancy [1, 2]. Mass spectrometric quantitative analysis of SAP in maternal plasma indicated that at the 10^th^-12^th^ week of pregnancy SAP was increased by a mean of 20 % in DS pregnancies compared to controls, while at the 15^th^-16^th^ week of pregnancy SAP was increased by a mean of 40 % [2, 3]. Related to Turner syndrome (TS) proteomic analysis of maternal plasma revealed that 9 proteins were significantly increased in the plasma of women carrying TS fetuses, whereas 3 were found to be decreased [4]. Furthermore, 3 proteins were found to be up-regulated in samples obtained from pregnancies with Klinefelter syndrome foetuses, whereas 4 proteins were down-regulated when compared to proteins detected in samples from normal foetuses. Related to the prevention of pregnancy complications, a number of proteins were found up-regulated in the maternal plasma at the 10th-12th in pregnancies which developed preeclampsia later in pregnancy. Proteomics offers a useful approach for the identification of biomarker, for prediction of fetal abnormalities and prevention of pregnancy complications, while the quantitative measurement of these biomarkers by mass spectrometry in maternal plasma will provide a reliable, multiplex, faster and cheaper screening NIPT approach [5].

References

1. Kolialexi A, Tsangaris GT, Papantoniou N, Anagnostopoulos AK, Vougas K, Bagiokos V, et al. Application of proteomics for the identification of differentially expressed protein markers for Down syndrome in maternal plasma. Prenat Diagn 2008;28(8):691-8.

2. Anagnostopoulos A, Tsangaris GT. Serum amyloid-p (SAP), a potential biomarker for Down syndrome fetuses prevention in maternal plasma. EPMA J. 2014;5(Suppl 1):A98.

3. Heywood W, Wang D, Madgett TE, Avent ND, Eaton S, Chitty LS, et al. The development of a peptide SRM-based tandem mass spectrometry assay for prenatal screening of Down syndrome. J Proteomics 2012;75(11):3248-3257.

4. Kolialexi A, Anagnostopoulos AK, Papantoniou N, Vougas K, Antsaklis A, Fountoulakis M, et al. Potential biomarkers for Turner in maternal plasma: possibility for noninvasive prenatal diagnosis. J Proteome Res. 20109(10):5164-70.

5. Kolialexi A, Anagnostopoulos AK, Tounta G, Antsaklis A, Mavrou A, Tsangaris GT. Biomarker development for non-invasive prenatal diagnosis of fetal aneuploidies: predictive reliability and potential clinical application. EPMA J. 2011;2(2):157-61.

## A81 Integrated ecosystem for an integrated care model for heart failure patients including related comorbidities (ZENITH)

### José Verdú^1^, German Gutiérrez^1^, Jordi Rovira^2^, Marta Martinez^2^, Lutz Fleischhacker^3^, Donna Green^4^, Arthur Garson^4^, Elena Tamburini^5^, Stefano Cuomo^5^, Juan Martinez-Leon^6^, Teresa Abrisqueta^6^, Hans-Peter Brunner-La Rocca^7^, Tiny Jaarsma^8^, Teresa Arredondo^9^, Cecilia Vera^9^, Giuseppe Fico^9^, Olga Golubnitschaja^10^, Fernando Arribas^11^, Martina Onderco^12^, Isabel Vara ^12^, on behalf of ZENITH consortium

#### ^1^Medtronic Iberica S.A., Madrid, Spain; ^2^Telefonica, Madrid, Spain; ^3^Fleischhacker, Schwerte, Germany; ^4^Grand-Aides, Houston, Texas, USA; ^5^I+ SRL, Firenze, Italy; ^6^Hospital G.U. Valencia, Valencia, Spain; ^7^Maastricht U. Medical Centre, Maastricht, The Netherlands; ^8^Linkopings Universitet, Linkopings, Sweden; ^9^Universidad Politecnica de Madrid, Madrid, Spain; ^10^European Association for Predictive, Preventive and Personalised Medicine, Brussels, Belgium; ^11^Hospital U. 12 de Octubre, Madrid, Spain; ^12^Elsevier, Amsterdam, The Netherlands

##### **Correspondence:** José Verdú (jose.verdu@medtronic.com) – Medtronic Iberica S.A., Madrid, Spain

**Keywords:** Heart failure, Co-morbidities, Integrated ICT platform, New patients' services, Personalization of services, Social actor, Standarized clinical assessment, Security & data privacy, 3 pilots

**Goal**

ZENITH aims to design and validate a new integrated ecosystem for an integrated care for heart failure (HF) patients including related co-morbidities, defining a new organizational model with the ability to improve quality of care for patients and to support the sustainability of the healthcare systems enabling continuity of care.

**Scientific objectives:**

ZENITH pursues the following objectives:

1) Stratification model and categorization of patients.

2) Personalization of the clinical interventions for HF patients dealing with their co-morbidities simultaneously.

3) Development of a unique ICT platform to be used by all the stakeholders.

4) Design of patient-oriented services according to the categorization of patients.

5) Use of the social workers into the integrated ecosystem.

6) Define a new organizational model to provide a complete communication and coordination between GPs, specialist, patient and carers.

7) Improve the evidence on health outcomes, QoL and care efficiency.

**Technological approach**

ZENITH is based in one ICT platform capable of collecting data from different centres (primary care, hospitals, outpatient clinics and patients’ home) and different stakeholders (general practitioners, specialists, nurses, social workers, patients and relatives). Different sources provide information to the ICT platform: typical parameters (i.e. blood pressure, weight, heart rate), implanted cardiac devices information, long-term subcutaneous recorders (24hx7dx365d), medium-term wearable recorders, hospital information systems and primary care data.

**Impacts**

The achievable impacts thanks to the use of the integrated ecosystem model proposed in ZENITH are:

1) Reduction in: i) all cause heart failure 30-day readmissions, ii) walk-in clinic visits in primary care, iii) the emergency department visits, and iv) days spent in care institutions.

2) Increase medication adherence at 1 year.

3) Improved cooperation between primary care and specialists and the interaction between patients, their carers and the social carer.

4) Reinforce primary care and specialists’ medical knowledge with respect to co-morbidities and poly-pharmacy.

5) Use an ICT system with all data available fully integrated, well organized, with clear information and reports to: i) help clinicians to make decisions with a global view of the HF and co-morbidities, ii) help patients to be remotely completely monitored with better QoL and less decompensations, iii) make sustainable the existing healthcare model, and iv) strengthen European industrial position in ICT products and services with new business areas and IPRs.

## A82 Predictive, preventive and personalized medicine in diabetes onset and complication (MOSAIC project)

### José Verdú^1^, Francesco Sambo^2^, Barbara Di Camillo^2^, Claudio Cobelli^2^, Andrea Facchinetti^2^, Giuseppe Fico^3^, Riccardo Bellazzi^4^, Lucia Sacchi^4^, Arianna Dagliati^4^, Daniele Segnani^5^, Valentina Tibollo^5^, Manuel Ottaviano^6^, Rafael Gabriel^6^, Leif Groop^7^, Jacqueline Postma^7^, Antonio Martinez^8^, Liisa Hakaste^9^, Tiinamaija Tuomi^9^, Konstantia Zarkogianni^10^, on behalf of MOSAIC consortium

#### ^1^Medtronic Iberica S.A., Madrid, Spain; ^2^Universita Degli Studi di Padova, Padova, Italy; ^3^Universidad Politecnica de Madrid, Madrid, Spain; ^4^Universita Degli Studi di Pavia, Pavia, Italy; ^5^Fondazione Salvatore Maugeri Clinica del Lavoro e Della Riabilitazione, Pavia, Italy; ^6^Asociacion Espanola para el desarrollo de La Epidemiologia Clinica, Madrid, Spain; ^7^Lunds Universitet, Lund, Sweden; ^8^Soluciones Tecnologicas para la Salud y el Bienestar (TSB), Valencia, Spain; ^9^Samfundet Folkhalsan I Svenska Finland RF, Helsingfors, Finland; ^10^National Technical University of Athens, Athens, Greece

##### **Correspondence:** José Verdú (coordinator@mosaicproject.eu) – Medtronic Iberica S.A., Madrid, Spain

**Keywords:** T2DM risk stratification, T2DM prediction of complication, Decision support system

The global prevalence of diabetes is 9 % of the world’s population; TD2M represents the 90 % of the cases [1]. The TD2M’s condition is the main risk factor for death and no-fatal complications; it causes a large burden to patients, families and the healthcare system. To reduce the long terms effects of this pandemic disease a strategy based on predictive, preventive and personalized medicine (PPPM) is required: develop risk calculation tools for diabetes onset and complications and develop early screening and intervention programs.

This abstract describes the achievements of the MOSAIC project that developed mathematical models to improve the current standards and practice for diagnosis of T2DM and also pre-diabetic states (IGT and IFT) as well prognostic tools to assess the risk of complications in diagnosed cases. The models have been trained using a large European datasets.

This research is based on three pillars: Create models to discover new indicators for onset and evolution of the disease, stratify the risks at population level and integrate the tools into the existing decision support systems of hospitals, clinical centres and health agencies.

The prediction of diabetes evolution is based on a Cox survival model and provides the curve of patient’s risks from 2 to 12 next years. The model was assessed with the Areas Under the ROC Curve (AUCs) trough 100 internal train/test splits and the external validation obtaining, in average AUC = 0.90, which outperforms the scores obtained by the “state of the art” models such as FINDRISC and FRAMINGHAM (AUC = 0.77) [2, 3].

The complications models exploit Logistic Regression (LR) and Naïve Bayes to predict the onset of Retinopathy, Neuropathy or Nephropathy, at 3, 5 and 7 years from the first visit at the hospital. The predictive power of our approach is estimated with a leave-one-out cross-validation, on witch AUC varied between 0.832 (LR, Retinopathy) and 0.647 (LR, Neuropathy) for 3 years horizon.

The predictive models have been integrated in a Web based platform that connects to the clinical data-warehouse of the clinics to be used in the following contexts: screening and monitoring of population (health agency), diagnostic and risk assessment (primary care), and evolution and management of the disease (secondary care).

**References**

1. WHO (World Health Organization), Global status report on noncommunicable diseases 2014, WHO. Available at: http://www.who.int/nmh/publications/ncd-status-report-2014/en/ Accessed 10 Jun 2015.

2. Lindström J, Tuomilehto J. The Diabetes Risk Score: a practical tool to predict type 2 diabetes risk. Diabetes Care. 2003;26:725–31.

3. Matthews DR, Hosker JP, Rudenski AS, Naylor BA, Treacher DF, Turner RC. Homeostasis model assessment: insulin resistance and beta-cell function from fasting plasma glucose and insulin concentrations in man. Diabetologia. 1985;28(7):412–9.

## A83 Possibilities for personalized therapy of diabetes using *in vitro* screening of insulin and oral hypoglycemic agents

### Igor Volchek^1^, Nina Pototskaya^2^, Andrey Petrov^1^

#### ^1^DiscoveryMed Ltd, St. Petersburg, Russia; ^2^Belarus State Medical University, Minsk, Belarus

##### **Correspondence:** Igor Volchek (ivolchek@discoverymed.ru) – DiscoveryMed Ltd, St. Petersburg, Russia

**Keywords:** Diabetes, Insulin, Oral hypoglycemic agents, Personalized medicine, Drugs screening

Currently, the important role of antioxidant system violations, including the thiol-disulfide (SH/SS) equilibrium of blood in the pathogenesis of diabetes is proved. We have developed an original method for screening drugs, based on the study of their *in vitro* effects on SH/SS blood ratio, where whole blood with anticoagulant (EDTA) was incubated in thermostat (37 °C) in the presence of drugs for 1 h, the control samples was incubated with saline [1]. Determination of SH-and SS-groups in hemolisate was done by spectrophotometry. In processing the data performance SH-groups and SH/SS ratio in the control and experimental samples were compared. In controlled clinical trials in patients with endometriosis it was demonstrated that using the proposed method for companion diagnostics have improved the effectiveness of personalized hormone treatment 2 times in comparison with standard one [2]. In studying the effect of various drugs and forms of insulin and oral hypoglycemic agents (Diabeton, Glibenclamide, Glurenorm, Metformin) in different doses on the SH/SS ratio of the blood of patients with diabetes has been shown that the response of each patient was purely personal [3]. For each patient, it was possible to select the optimal drug and dose, which usually do not coincide with therapeutic ones, appointed on the basis of indicators of blood glucose levels. The method can be used for personalized therapy of diabetes and allows evaluating the dynamics of the sensitivity to the drugs during treatment as well as for patient-based drug discovery. The method has a huge market potential and is currently used in laboratories in Russia and the United States. Controlled clinical trials of this technique for patients with diabetes are necessary.

**References**

1. Volchek I. Method for screening drug preparations. EP 1, 182, 455; RU 2,150,700; US 6,627,452.

2. Volchek I, Petrov A. Personalized therapy of genital endometriosis and cancer using drugs screening. Terra Medica. 2012; 2: 15 – 23.

3. Volchek I. Possibility of using the screening of insulin and oral hypoglycemic agents for patients-based drug discovery. BIT’s 3^rd^ Annual World Congress of Diabetes-2014, Haikou, China. p. 141

## A84 The innovative technology for personalized therapy of human diseases based on *in vitro* drug screening

### Igor Volchek^1^, Nadezhda Pototskaya^2^, Andrey Petrov^1^

#### ^1^DiscoveryMed Ltd, St. Petersburg, Russia; ^2^Belarus State Medical University, Minsk, Belarus

##### **Correspondence:** Igor Volchek (ivolchek@discoverymed.ru) – DiscoveryMed Ltd, St. Petersburg, Russia

**Keywords:** Personalized therapy, Thiol-disulfide (SH/SS) system, *In vitro* drug screening, Chronic hepatitis C, Chronic bronchitis, Endometriosis, Non-small cell lung cancer

The original method for drug screening based on it in *vitro* effect on blood thiol-disulfide (SH/SS) ratio was proposed for personalized therapy [1]. The study involved 325 patients with chronic hepatitis C (HCV), chronic bronchitis, endometriosis and non-small cell lung cancer (NSCLC). The controlled trials shown 3-fold increase of HCV viral response rate after personalized therapy with interferon (IFN) compared to standard (74.2 % vs. 25.0 %) [2], 2-fold increase efficiency of personalized hormonal therapy of endometriosis compared to standard (85.4 % vs. 42.5 %) and 2-fold increase frequency of partial remission rate of NSCLC after personalized cytostatic therapy in comparison with standard one (36 % vs.17 %) [3, 4]. The sensitivity of the method was 89.2 – 89.8 %, and its specificity – 94.6 %. The incidence of adverse effects of IFN was decreased 6 times compared to standard therapy (12 % vs. 72 %). At chronic bronchitis the incidence of unsatisfactory clinical results or progression was three times less (11 % vs.33 %), the incidence of unsatisfactory bacteriological results was 4 times rarely (7 % vs. 28 %), and the rate of side effects was more than two times less (10.5 % vs. 22.8 %) after personalized versus standard therapy by the same antibiotics [3, 4]. Thus, proposed method of screening drugs using SH/SS-test can be used to personalize therapy of human diseases by antibiotic, hormonal, antiviral, cytostatic and immune preparations to improve efficiency and overcome resistance to drugs, search the best drugs, their doses and combinations, reducing the frequency of side effects and complications.

**References**

1. Volchek I. Method for screening drug preparations. EP 1, 182, 455; RU 2,150,700; US 6,627,452.

2. Volchek I, Sologub T, Nowicky JW, Grigoryeva T, Belozyorova L, Belopolskaya M, et al. Preliminary results of individual therapy of chronic hepatitis C by ukrain and interferon-alpha. Drugs Exptl Clin Res. 2000;26:261-6.

3. Volchek I, Petrov A. The possibility for personalized therapy of human diseases: twelve years practice. Terra Medica. 2010;3:3-11.

4. Volchek I, Petrov A. Personalized therapy of genital endometriosis and cancer using drugs screening. Terra Medica. 2012;2:15-23.

## A85 Bone destruction and temporomandibular joint: predictive markers, pathogenetic aspects and quality of life

### Ülle Voog-Oras^1^, Oksana Jagur (Oksana.Ivask@kliinikum.ee)^2^, Edvitar Leibur (Edvitar.Leibur@kliinikum.ee)^3^, Priit Niibo (Priit.Niibo@kliinikum.ee)^1^, Triin Jagomägi (Triin.Jagomagi@ortodontia.ee)^1^, Minh Son Nguyen (minhson1883@gmail.com)^1^, Chris Pruunsild (Chris.Pruunsild@kliinikum.ee)^4^, Dagmar Piikov (Dagmar.Piikov@kliinikum.ee)^1^, Mare Saag (Mare.Saag@kliinikum.ee)^1^

#### ^1^University of Tartu, Department of Stomatology, Tartu, Estonia; ^2^Clinic of Dentistry, Department of Maxillofacial Surgery, Tartu, Estonia; ^3^University of Tartu, Department of Internal Medicine, Tartu, Estonia; ^4^University of Tartu, Department of Pediatrics, Tartu, Estonia

##### **Correspondence:** Ülle Voog-Oras (ylle.Voog@kliinikum.ee) – University of Tartu, Department of Stomatology, Tartu, Estonia

**Keywords:** Arthroscopy, Bone changes, Bone markers, Predictive preventive personalized medicine, TMJ

**Scientific objectives:** The main aims were to investigate the impact of pain in the temporomandibular joint (TMJ) and masticatory muscles on daily living; to investigate the progression of radiographic signs of bone destruction in the TMJ of patients with rheumatoid arthritis (RA) and wether this progression is related to the presence and levels in the blood of inflammatory mediators and markers; to relate the clinical findings to disease activity in juvenile idiopathic arthritis (JIA) patients; to elucidate the effect and efficacy of various TMJ treatment modalities.

**Technological approaches**. During the development of predictive, preventive, and personalized medicine many new and sensitive methods for earlier detection of bone destruction has been used in the management of TMJ disorders [1].

**Results interpretation:** A population based study showed a 47 % rate of TMJ clinical involvement and indicated that TMJ pain/discomfort exerts a negative influence on ADL and is related to the biochemical markers of bone turnover and the 25(OH)D level [2]. In malocclusion patients, TMJ dysfunction suggests the need for orthodontic treatment [3]. In RA patients, the electromyographic activity of masticatory muscles during TMJ movement was higher than in healthy subjects. A long term (7-8 yrs) investigation suggests that high levels of plasma concentration of the tumor necrosis factor, of erythrocyte sedimentation rate and of thrombocyte particle concentration predict progression of erosions and bone loss in the TMJ of patients with RA, while serum concentrations of serotonin seem to have an indirect effect. A long-term (5 yrs) study showed an increased frequency of fibrillations and fibrous adhesions as pathological findings following TMJ arthroscopy. Arthroscopic treatment of TMJ disorders is conducive to favourable long-term results with regard to increasing the maximum interincisal opening, reducing pain and eliminating TMJ dysfunction [4].

**Outlook and Expert recommendations:** The conduct of long-term studies represents an important contribution to predictive and preventive medicine. The assessment of TMJ pain, the biochemical markers of bone metabolism, mediators and treatment results helps to monitor the disease process. Interdisciplinary approach is important for the selection of correct personalized treatment modality and hence a better quality of life.

**References**

1. Schierbeck H, Pullerits R, Pruunsild C, Fischer M, Holzinger D, Laestadius Å, et al. HMGB1 levels are increased in patients with juvenile idiopathic arthritis, correlate with early onset of disease, and are independent of disease duration. J Rheumatol. 2013;40(9):1604-13.

2. Jagur O, Kull M, Leibur E, Kallikorm R, Loorits D, Lember M, et al. Relationship between radiographic changes in the temporomandibular joint and bone mineral density: a population based study. Stomatologija. 2011;13(2):42-8.

3. Nguyen SM, Nguyen MK, Saag M, Jagomagi T. The Need for Orthodontic Treatment among Vietnamese School Children and Young Adults. Int J Dent. 2014;2014:132301.

4. Leibur E, Jagur O, Müürsepp P, Veede L, Voog-Oras U. Long-term evaluation of arthroscopic surgery with lysis and lavage of temporomandibular joint disorders. J Craniomaxillofac Surg. 2010;38(8):615-20.

## A86 Sub-optimal health management – global vision for concepts in medical travel

### Wei Wang (wei.wang@ecu.edu.au)^1,2,3^

#### ^1^School of Medical Science, Edith Cowan University, Perth, Australia; ^2^Municipal Key Laboratory of Clinical Epidemiology, Capital Medical University, Beijing, China; ^3^Global Health Epidemiology Reference Group (GHERG), Edinburgh, UK

**Keywords:** Essential medicine, Essential diagnostics, Suboptimal health status, Preventive predictive personalized medicine, Medical Travel

The health professionals recognize the importance of ensuring people to have both adequate and reliable access to essential medicines and diagnosis while traveling. Health authorities need to know what to prioritize and ensure that the exiting basic health care system is able to adequately serve the needs of the traveling people. The history of travel medicines is relatively recent. The Faculty of Travel Medicine, Australasian College of Tropical Medicine was established in 2000 as the first faculty of Travel Medicine globally [1].

Although the history of Essential Medicine (EM) is recent considering that aspirin introduced in 1897 is the first synthetic pharmaceutical, and is the first EM aiming to provide and satisfy the health care needs of the majority, the concept of EM has been already recognized globally [2]. In 1977, the WHO launched the “Model List of EM”, and identified 208 individual medicines which could provide safe, cost-effective and time-efficient treatments for common diseases [2].

With the arrival of Big Data and many Biomarker claims, along with EM, developing Essential Diagnostics (ED) will be critical for Preventive, Predictive and Personalized Medicine (PPPM) [3]. Developing such a Model List of ED would provide an opportunity to harness the advanced diagnostic technology and link it with health policies, accountable to global communities [2, 3].

Suboptimal Health Status (SHS) is a pre-clinical physical status between health and disease, and is characterized by the perception of health complaints, general weakness, and low energy. It is regarded as a subclinical, reversible stage of chronic disease [3-5]. SHS is also the one of the most common subjective, self-measurable health phenotypes during and after people’s traveling.

Author proposes how we combine the merits of EM, ED, and SHS to apply them in PPPM practice.

**References**

1. The Selection and Use of Essential Medicines - WHO Technical Report Series, No. 914. http://apps.who.int/medicinedocs/en/d/Js4875e/5.2.html Accessed 20 Jun 2015

2. Dove ES, Barlas IÖ, Birch K, Boehme C, Borda-Rodriguez A, Byne WM et al. An Appeal to the Global Health Community for a Tripartite Innovation: An “Essential Diagnostics List”, “Health in All Policies” and “See-Through 21st Century Science and Ethics”. OMICS 2015;19(8):435-42.

3. Wang W, Russell A, Yan Y; Global Health Epidemiology Reference Group (GHERG). Traditional Chinese medicine and new concepts of predictive, preventive and personalized medicine in diagnosis and treatment of suboptimal health. EPMA J. 2014;5:4. doi:10.1186/1878-5085-5-4.

4. Yan YX, Dong J, Liu YQ, Yang XH, Li M, Shia G, et al. Association of suboptimal health status and cardiovascular risk factors in urban Chinese workers. J Urban Health. 2012;89(2):329-38.

5. Yan YX, Liu YQ, Li M, Hu PF, Guo AM, Yang XH, et al. Development and evaluation of a questionnaire for measuring suboptimal health status in urban Chinese. J Epidemiol. 2009;19(6):333-41.

## A87 Sub-optimal health management: synergic PPPM-TCAM approach

### Wei Wang (wei.wang@ecu.edu.au)^1,2,3^

#### ^1^School of Medical Science, Edith Cowan University, Perth, Australia; ^2^Municipal Key Laboratory of Clinical Epidemiology, Capital Medical University, Beijing, China; ^3^Global Health Epidemiology Reference Group (GHERG), Edinburgh, UK

**Keywords:** Predictive preventive personalized medicine, Sub-optimal health, Stress

A full characterization of consistent phenotypes which defines the general population is the basic step to individual difference normalization in predictive, preventive and personalized medicine (PPPM). Self-claimed normal healthy status might not represent the real healthy condition due to the facts that asymptomatic subjects may carry chronic diseases at their early stage, e.g. atherosclerosis, diabetes mellitus and hypertension. Currently, treatments for chronic diseases are implemented after clinical diagnosis, which is a very much delayed-approach from the perspective of PPPM [1]. This abstract examples the suboptimal health status (SHS) which is a new PPPM challenge in a cohort of people with ambiguous health complaints, e.g. general weakness, chronic fatigue syndrome, and myalgic encephalomyelitis [1-4]. An example of using Copenhagen Psychosocial Questionnaire (COPSOQ) in combination with Suboptimal Questionairee-25 (SHSQ-25) to assess job-related psychosocial stress will be reported. The mean value of the five scales of COPSOQ and distribution of plasma cortisol and mRNA expression of GRa/GRb between the high and low levels of SHS groups were compared using multiple linear regression analysis. We have identified 3 factors that are predictive of SHS, i.e. 1) demands at work, 2) interpersonal relations and leadership, and 3) insecurity at work. Higher levels of plasma cortisol and GRb/GRa mRNA ratio were observed in the high SHS-score group [2]. SHS is correlated with decreased mRNA expression of GRa [2] and also n-glycan profiling [5]. The study confirmed the association between chronic psychosocial stress and SHS, demonstrating that improving psychosocial work environment may reduce SHS and prevent chronic diseases with the PPPM approach.

**References**

1. Wang W, Russell A, Yan Y; Global Health Epidemiology Reference Group (GHERG). Traditional Chinese medicine and new concepts of predictive, preventive and personalized medicine in diagnosis and treatment of suboptimal health. EPMA J. 2014;5:4. doi:10.1186/1878-5085-5-4.

2. Yan YX, Dong J, Liu YQ, Zhang J, Song MS, He Y, et al. Association of suboptimal health status with psychosocial stress, plasma cortisol and mRNA expression of glucocorticoid receptor in lymphocyte. Stress. 2015;18(1):29-34.

3. Yan YX, Dong J, Liu YQ, Yang XH, Li M, Shia G, et al. Association of suboptimal health status and cardiovascular risk factors in urban Chinese workers. J Urban Health. 2012;89(2):329-38.

4. Yan YX, Liu YQ, Li M, Hu PF, Guo AM, Yang XH, et al. Development and evaluation of a questionnaire for measuring suboptimal health status in urban Chinese. J Epidemiol. 2009;19(6):333-41.

5. Lu JP, Knežević A, Wang YX, Rudan I, Campbell H, Zou ZK, et al. Screening novel biomarkers for metabolic syndrome by profiling human plasma N-glycans in Chinese Han and Croatian populations. J Proteome Res. 2011;10(11):4959-69.

## A88 Innovative technologies for minimally invasive diagnostics

### Andreas Weinhäusel, Walter Pulverer, Matthias Wielscher, Manuela Hofner, Christa Noehammer, Regina Soldo, Peter Hettegger, Istvan Gyurjan, Ronald Kulovics, Silvia Schönthaler, Gabriel Beikircher, Albert Kriegner; Stephan Pabinger, Klemens Vierlinger

#### Molecular Diagnostics, AIT- Austrian Institute of Technology GmbH, Vienna, Austria

##### **Correspondence:** Andreas Weinhäusel (andreas.weinhaeusel@ait.ac.at) – Molecular Diagnostics, AIT- Austrian Institute of Technology GmbH, Vienna, Austria

**Keywords:** Biomarker development, Minimally invasive diagnostics, DNA methylation, Autoantibody, Multiplexing

**Scientific objectives:** An estimated 2.7 million new cancer cases and 7.6 million cancer related deaths were reported worldwide in 2008 and incidences are increasing. It is well accepted that early cancer diagnosis can improve survival, thus there is a great need and anticipation to identify novel biomarkers for cancer diagnosis at the earliest possible stage, which can ideally be integrated in minimally invasive diagnostic assays. Epigenetic changes are a hallmark of cancer can be used as markers for detection of circulating tumor DNA in serum/plasma as well in saliva samples. In addition cancer onset and progression produces mutated or aberrantly expressed proteins generally also termed as tumor associated antigens (TAAs) which are able to act as antigens and evoke an immune response which results in the production of autoantibodies. These autoantibodies and the early DNA methylation changes during neoplastic transformation are able to be detected months or years before the clinical diagnosis of cancer and can therefore be used as biomarkers for the early diagnosis of cancer.

**Technological approaches:** We have setup genome- and immunome-wide biomarker-discovery as well a targeted technologies using multiplexed technologies for validation of these markers. Combining the relevant technologies we call these MethPipe and PepPipe for enabling us streamlined biomarker-development for DNA-methylation and Antibody-based diagnostics.

**Results interpretation:** Plasma derived cell free DNA methylation maker qualification for early diagnosis of lung cancer has been conducted successfully enabling AUC = 0,85-0,92. Similarly the serum-autoantibody based biomarker discovery enables high correct classification (AUC >0,9) for distinguishing cases vs control for the big 4 cancer entities. Thus as exemplified both cell free DNA-methylation analyses and antibody-profiling technologies have a high potential for minimally invasive diagnostics.

**Outlook and Expert recommendations:** These strategies for enabling *minimally invasive* testing of patients are well suited for disease biomarker definition – and will be useful for enabling personalized medicine by qualification of diagnostic, prognostic, and predictive biomarkers.

**Acknowledgements**

This work was supported by grants from “OeNB Jubiläumsfonds”, Life Science Krems, TECNET, the Vienna Science and Technology fund WWTF, and EU FP7 and H2020 funds (RESOLVE and ULTRAPLACAD).

## A89 Rare disease diobanks for personalized medicine

### Ayşe Yüzbaşıoğlu^1,2^, Meral Özgüç^1,2^

#### ^1^Hacettepe University Center for Biobanking and Genomics, DNA/Cell Bank for Rare Diseases, Ankara, Turkey; ^2^Department of Medical Biology, Hacettepe University, Ankara, Turkey

##### **Correspondence:** Meral Özgüç (meralozguc51@gmail.com) – Hacettepe University Center for Biobanking and Genomics, DNA/Cell Bank for Rare Disease, Ankara, Turkey

**Keywords:** Rare diseases, Biobanking, Networking, Targeted therapies

In the EU, rare diseases (RDs) are defined as life threatening and debilitating diseases with a prevalance of less than 5 per 10.000 persons. Even if the number of individuals with a specific disease is low, there are about 8.000 RDs that affects millions of individuals worldwide. Unfortunately for health care systems, RDs are “orphan” diseases and there is still a lack of capacity building for timely diagnosis, management and treatment. Thus national and international collaborations are crucial to reach an adequate number of patients for biomedical research and clinical trials [1]. In this respect biobanks are valuable infrastructures where patient samples and associated clinical and personal data can be stored with proper governance covering quality assurance of sample storage and ethical guidelines for acquisition of and access to samples and data [2].

Since more than 80 % of RDs are monogenic, identification of new disease genes and their functional annotation to understand the disease pathogenesis, will lead to the design and development of new targeted/personalized therapies relying on accurate stratification of patients based on “omics” data sets [3]. Here biobanks play an important role in securing different biological samples from the same individual. Moreover banking samples from patients with a wide spectrum of phenotypes provide insights into the genetic and other modifiers of the disease phenotype and this is an added value for designing a personalized approach for treatment. Today there are different RDs consortia such as RD-Connect, IRDIRC, EuroBiobank, RDCRN through these cooperations, harmonization of ethical guidelines and SOPs will become available all to the benefit of the RDs patients and their individualized treatments in the near future [4].

**References**

1. International Rare Diseases Research Consortium. http://www.irdirc.org/ Accessed 10 Jan 2015.

2. Yüzbaşıoğlu A, Özgüç M. Biobanking: sample acquisition and quality assurance for 'omics' research. N Biotechnol. 2013;30(3):339-342

3. di Donato J-H. Biobanking for Rare Diseases – Impact on Personalised Medicine. In: Özgüç M, editor. Rare Diseases: Integrative PPPM Approach as the Medicine of the Future. Springer: 2015, pp. 23-31

4. RD-CONNECT | An integrated platform connecting databases, registries, biobanks and clinical bioinformatics for rare disease research. http://www.rd-connect.eu/ Accessed 10 Jan 2015

